# Senescence‐Associated Loss of α‐Synuclein in Brain Microvascular Endothelium Compromises Tight Junctions and Disrupts Blood–Brain Barrier Integrity

**DOI:** 10.1111/acel.70770

**Published:** 2026-11-09

**Authors:** Ziwei Wang, Yoichi Takami, Cheng Wang, Yoshitaka Kase, Hiroshi Koriyama, Jittoku Ihara, Yu Guo, Weidong Liu, Nanxiang Yin, Lisha Yi, Daisuke Nawa, Hikari Takeshita, Shuko Takeda, Akitoshi Hara, Toshimasa Takahashi, Munehisa Shimamura, Hironori Nakagami, Yasufumi Kaneda, Koichi Yamamoto

**Affiliations:** ^1^ Department of Geriatric and General Medicine The University of Osaka Graduate School of Medicine Osaka Japan; ^2^ Division of Geroscience, International Center for Brain Science (ICBS) Fujita Health University Toyoake‐shi Aichi Japan; ^3^ Department of Geriatric Medicine, Graduate School of Medicine The University of Tokyo Tokyo Japan; ^4^ Department of Clinical Gene Therapy The University of Osaka Graduate School of Medicine Osaka Japan; ^5^ Osaka Psychiatric Research Center Osaka Psychiatric Medical Center Osaka Japan; ^6^ Department of Medicine University of Toronto Toronto Ontario Canada; ^7^ Department of Gene & Stem Cell Regenerative Therapy The University of Osaka Graduate School of Medicine Faculty of Medicine Osaka Japan; ^8^ Department of Neurology The University of Osaka Graduate School of Medicine Osaka Japan; ^9^ Department of Health Development and Medicine The University of Osaka Graduate School of Medicine Osaka Japan; ^10^ Center for Infectious Disease Education and Research The University of Osaka Osaka Japan

**Keywords:** blood–brain barrier, brain microvascular endothelium, cerebral small vessel disease, endothelial senescence, tight junction, α‐synuclein, β‐site amyloid precursor protein cleaving enzyme 1

## Abstract

Blood–brain barrier (BBB) dysfunction is an early feature of brain aging, but the endothelial mechanisms preserving barrier resilience remain unclear. Although we have reported α‐synuclein (SNCA) supports vascular endothelial homeostasis, its role in the brain microvascular endothelium is unknown. Because SNCA loss suppresses eNOS/NO signaling and promotes endothelial β‐site amyloid precursor protein cleaving enzyme 1 (BACE1) induction, we investigated whether senescence‐associated SNCA decline drives BACE1‐dependent BBB injury. Replicative senescence in human brain microvascular endothelial cells (HBMECs) reduced SNCA expression. siRNA‐mediated SNCA depletion induced a senescence‐like state with increased β‐galactosidase activity, CDKN2A/p16 and TP53/p53 induction, and decreased SIRT1, eNOS, and glypican‐1. SNCA‐deficient HBMECs showed impaired proliferation and migration, loss of occludin and ZO‐1, increased permeability, and elevated BACE1 expression and activity; BACE1 co‐silencing restored junctional proteins and normalized permeability. Transcriptomic and proteomic profiling excluded most classical BACE1‐regulatory mechanisms and instead implicated coordinated disruption of the NO–sGC–cGMP axis, consistent with the partial rescue by the NO donor SNAP. Amyloid‐β42 oligomers sensitized SNCA‐deficient monolayers to barrier injury at concentrations that left control monolayers unaffected. Global SNCA‐knockout mice exhibited multiregional BBB leakage, reduced occludin, ZO‐1, and claudin‐5, and p53 immunoreactivity confined to CD31‐positive microvessels. This phenotype was present in young adulthood and was reproduced in aged wild‐type mice, which showed reduced SNCA in isolated brain microvessels. These findings identify senescence‐associated endothelial SNCA loss as a candidate contributor to brain microvascular barrier vulnerability, describing an endothelial stress pathway in which SNCA insufficiency is associated with BACE1‐dependent tight junction destabilization in the aging brain.

## Graphical abstract

Decline of SNCA in brain microvascular endothelium during vascular aging induces a senescence‐like stress state with p16/p53 activation, impaired SIRT1‐eNOS signaling, and reduced repair capacity. This is accompanied by BACE1 upregulation, tight‐junction disruption, BBB leakage, and Aβ42 vulnerability; global SNCA‐knockout mice show p53 accumulation confined to CD31‐positive microvessels and multiregional barrier failure.

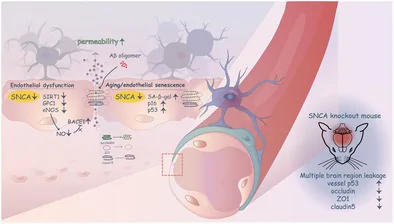

## 1 Introduction

Late‐life dementia commonly reflects a convergence of neurodegenerative and cerebrovascular pathology. In older adults, the vascular contribution to cognitive impairment often arises not from carotid or major intracranial arteries, but from penetrating arterioles, capillaries, and venules, as in cerebral small vessel disease, where aging and risk factors such as hypertension and diabetes promote arteriolosclerosis, microhemorrhage, endothelial dysfunction, and blood–brain barrier (BBB) disruption (Mogi et al. 2026; Santisteban et al. 2023). Alzheimer's disease shares many risk factors, and amyloid β (Aβ) accumulates in cerebral vessels as well as parenchyma, where cerebral amyloid angiopathy influences disease progression (Mogi et al. 2026; Sweeney et al. 2018; Zlokovic et al. 2020). Cerebrovascular dysfunction is thus a major driver of late‐life dementia, and molecules linking aging and vascular stress to brain microvascular endothelial dysfunction may be attractive therapeutic targets (Luissint et al. 2012; Sweeney et al. 2018).

α‐Synuclein (SNCA) is best known for its pathogenic role in synucleinopathies such as Parkinson's disease, whereas its physiological function outside the nervous system remains less defined. We previously showed that SNCA is expressed in and secreted by vascular endothelial cells, declines with cellular and organismal aging, and that circulating SNCA decreases with age‐related vascular dysfunction, diabetes, and insulin resistance (Rodriguez‐Araujo et al. 2015; Takami et al. 2022). Monomeric SNCA exerts endothelial‐protective effects via Akt–eNOS signaling, loss of which may contribute to hypertension and insulin resistance (Rodriguez‐Araujo et al. 2013; Takami et al. 2022), and restrains Weibel–Palade body exocytosis (Kim et al. 2010). Whether this age‐associated endothelial decline is relevant to brain microvascular pathology, potentially bridging lifestyle‐related vascular disorders and dementia‐related cerebrovascular injury, has not been established.

β‐Site APP cleaving enzyme 1 (BACE1) is a candidate mediator of SNCA loss in brain microvascular endothelium: endothelial SNCA loss suppresses Akt–eNOS signaling and weakens nitric oxide (NO)‐dependent vascular homeostasis (Takami et al. 2022); replicative senescence of HBMECs itself increases BACE1 (Sun et al. 2018); and loss of endothelial NO signaling increases BACE1 and amyloidogenic processing in cerebral microvessels, while BACE1 inactivation enhances eNOS/glypican‐1 (GPC1) and excessive BACE1 disrupts tight junctions by cleaving occludin (Austin et al. 2013, 2010; He et al. 2022; Katusic et al. 2024; Zhou et al. 2022), together prioritizing BACE1 as a downstream effector of SNCA deficiency.

We hypothesized that loss of endogenous SNCA in brain microvascular endothelial cells drives a senescence‐associated stress state converging on BACE1 induction, junction disorganization, and BBB leakage. We investigated whether SNCA, an aging‐associated endothelial molecule, contributes to brain microvascular endothelial dysfunction and BBB injury, processes implicated in the cerebrovascular component of late‐life cognitive impairment, and whether it may represent a shared molecular substrate linking these processes to lifestyle‐related disorders such as hypertension and diabetes. We combined replicative senescence and siRNA loss‐of‐function studies in HBMECs with mechanistic rescue experiments and in vivo analyzes of SNCA‐deficient mice, and this may inform future studies addressing whether the endothelial SNCA–BACE1 axis can be targeted to preserve BBB integrity in the aging brain.

## 2 Materials and Methods

### 2.1 Cell Culture and Induction of Replicative Senescence

Primary HBMECs (Cell Systems, Kirkland, WA, USA, Cat. No. ACBRI 376) were cultured in EGM‐2 BulletKit (Lonza, Walkersville, MD, USA; Cat. No. CC‐3162), comprising EBM‐2 with 2% FBS and SingleQuots (FGF, VEGF, IGF, EGF, ascorbic acid, hydrocortisone, gentamicin/amphotericin B, heparin), at 37°C/5% CO_2_. Cells between passages 5–25 were serially passaged to induce replicative senescence; early‐passage cultures served as young controls and late‐passage cultures as senescent cells. Normal human astrocytes (NHA; Lonza; Cat. No. CC‐2565) were cultured in AGM BulletKit (Cat. No. CC‐3186, with ABM Cat. No. CC‐3187 + SingleQuots Cat. No. CC‐4123). Primary human brain pericytes (Cell Systems; Cat. No. ACBRI 498) were cultured in Complete Classic Medium with Serum and CultureBoost (Cat. No. 4Z0‐500); these cells were used for the comparison in Figure S5, at passage 3 (< 12 cumulative population doublings).

### 2.2 siRNA‐Mediated Gene Knockdown and Plasmid‐Mediated BACE1 Overexpression

Cells at passages 5–8 were used for siRNA experiments. HBMECs were transfected with siRNAs targeting human SNCA (siSNCA, Santa Cruz Biotechnology, Dallas, TX, USA; Cat. No. sc‐29619) and/or BACE1 (siBACE1, Cat. No. sc‐372245) or a non‐targeting control (scramble, Cat. No. sc‐37007) using Lipofectamine RNAiMAX (Thermo Fisher Scientific; Cat. No. 13778150) per the manufacturer's instructions; final concentration was 50 nM (single) or 25 nM each (co‐knockdown). Cells were harvested 72 h post‐transfection. siRNA details are in Table S1. For BACE1 overexpression, HBMECs were transfected with 1.5 μg pcDNA3.1‐Zeo_BACE1 plasmid or empty vector using Lipofectamine 3000 (Cat. No. L3000015); medium was replaced at 6 h, and protein/RNA were harvested at 48 h total. Plasmids are listed in Table S2.

### 2.3 Preparation of Oligomeric Amyloid β42 (Aβ42) and Thioflavin T Assay

Amyloid β‐protein (1–42) (Peptide Institute, Ibaraki, Osaka, Japan; Cat. No. 4349‐v) was prepared per the manufacturer's instructions; oligomers were generated by incubating monomeric Aβ42 at 37°C for 8–10 h, confirmed by thioflavin‐T (Thermo Scientific Chemicals; Cat. No. 211760050) fluorescence. Concentrations are monomer‐equivalent, not molar oligomer‐particle concentration; because Aβ42 adsorbs to plasticware and serum proteins, free concentration reaching cells is expected to be lower than nominal. The 1 and 5 μM experiments and DMSO controls used a single batch, distinct from the 10 μM batch; all comparisons were made within a batch. HBMEC monolayers were treated with Aβ42 oligomers (1, 5, or 10 μM; vehicle: DMSO, FUJIFILM Wako, Osaka, Japan; Cat. No. 043‐07216).

### 2.4 In Situ Staining and Quantitative Analysis of SA‐β‐Gal Activity

Senescence was assessed by in situ staining for SA‐β‐gal using a senescence detection kit (BioVision; Cat. No. K320‐250). Quantitative SA‐β‐gal activity used the 96‐well cellular senescence assay kit (Cell Biolabs; Cat. No. CBA‐231).

### 2.5 Quantitative Reverse Transcription‐PCR (qRT‐PCR)

Total RNA was extracted with Sepasol‐RNA I Super G (Nacalai Tesque; Cat. No. 09379‐84); cDNA was synthesized with the ReverTra Ace qPCR kit (Toyobo; Cat. No. FSQ‐101). qRT‐PCR ran on an Applied Biosystems 7900HT system using SYBR Green master mix (MedChemExpress; Cat. No. HY‐K0501): 95°C/30 s, then 40 cycles of 95°C/5 s and 60°C/40 s, followed by melting‐curve analysis. Expression was analyzed by the ΔΔCt method, normalized to GAPDH. Primers are listed in Table S3.

### 2.6 Western Blotting

Lysates were prepared in M‐PER (Thermo Fisher Scientific; Cat. No. 78501) with protease inhibitor (Roche; Cat. No. 11873580001) and PhosSTOP (Roche; Cat. No. 04906837001), clarified at 16,000 × g/15 min/4°C. Protein was quantified with the DC assay (Bio‐Rad; Cat. No. 5000116). Equal protein was separated by 5%–20% Bullet PAGE Plus gels (Nacalai Tesque) and transferred to 0.45‐μm PVDF (Invitrogen; Cat. No. LC2005). Membranes were blocked (5% nonfat milk), probed with primary antibodies overnight at 4°C (Table S4), then HRP‐conjugated secondary antibodies (Cytiva). Signals were detected with an ImageQuant LAS 4000 mini using Chemi‐Lumi One Super (Nacalai Tesque) and quantified in ImageJ, normalized to loading control. Full unedited membranes are provided as a separate Supporting Information S1 labeled by panel.

### 2.7 Cell Proliferation and Migration Assays

Viability was assessed by MTS assay (Promega; Cat. G3582) at 490 nm. Transwell migration used 24‐well inserts with 8.0‐μm PET membranes (Millipore; Cat. No. PTEP24H48): apical medium was EBM‐2/0.5% FBS, basolateral EBM‐2/10% FBS; after 24 h, migrated cells were fixed (4% PFA), stained with DAPI, and counted (BZ‐X800L, Keyence). Scratch assays on confluent monolayers used a sterile pipette tip; images at 0, 24, 48 h were quantified in ImageJ as percent migrated area relative to time 0.

### 2.8 Cell Immunofluorescence and Junctional Continuity Analysis

HBMECs were fixed (4% paraformaldehyde), permeabilized (0.1% Triton X‐100), and blocked with 5% goat serum, then incubated with primary antibodies overnight at 4°C and fluorophore‐conjugated secondary antibodies for 1 h. Nuclei were counterstained with DAPI; images were acquired on a confocal microscope (FLUOVIEW FV4000, Evident). Junctional continuity was quantified blindly from line‐based measurements along cell borders (ImageJ/FIJI). Antibodies are in Table S4.

### 2.9 BACE1 Activity Assay

BACE1 activity was measured with a fluorometric beta‐secretase assay kit (BioVision; Cat. No. K360‐100), normalized to total protein.

### 2.10 Endothelial Permeability Assay

Paracellular permeability was assessed on 24‐well transwell inserts with 0.4‐μm PET membranes (Corning; Cat. No. 3470). At 48 h post‐transfection, FITC‐dextran (40 kDa, 0.5 mg/mL; Thermo Fisher Scientific; Cat. No. D1845) was added apically; basolateral samples were collected after 30 min–6 h and fluorescence read at 485/520 nm. Main‐figure data are expressed relative to control.

### 2.11 SNAP Rescue Assay

Scramble‐ or SNCA siRNA‐transfected HBMECs were treated at 48 h with the NO donor SNAP (200 μM; Sigma‐Aldrich; Cat. No. N3398) for 8 h; untreated cells served as controls. Protein was collected per Section 2.6.

### 2.12 RNA Sequencing and Transcriptomic Analysis

HBMECs transfected with scramble or SNCA siRNA (*n* = 3 independent transfections/condition) were harvested at 48 h. Libraries (Rhelixa, Tokyo) used the NEBNext Poly(A) Isolation Module and Ultra II Directional RNA Library Prep Kit, sequenced on an Illumina NovaSeq X Plus (150‐bp paired‐end, ~40 M reads/sample). Reads were quality‐trimmed (FastQC/Trimmomatic), aligned to GRCh38/hg38 with HISAT2, and counted with featureCounts; TPM/FPKM were derived from counts. Differential expression (scramble vs. SNCA‐silenced) used DESeq2 (*p*_adj_ < 0.05, |log2FC| > 1). PCA was performed on the log2‐transformed, DESeq2‐normalized, expression‐filtered count matrix, gene‐wise mean‐centered across six libraries without unit‐variance scaling.

Eight mechanism modules were defined a priori by literature‐guided curation of pathways with established potential to regulate BACE1: M1, ubiquitin–proteasome degradation; M2, translation/integrated stress response; M3, BACE1‐AS mRNA stability; M4, classical BACE1 promoter factors; M5, oxidative stress/NRF2–ARE; M6, ER stress/unfolded protein response; M7, the BACE1 genomic locus; M8, SIRT1/eNOS/NO–sGC–cGMP signaling (full membership in Figure 4L). Only module members with finite adjusted *p* values and log2FC contributed to module calculations; undetected/untested members were shown in gray but excluded. Module median fold change was 2^(median log2FC)^; a two‐sided Mann–Whitney *U* test compared module‐member log2FC distributions against all other tested genes. Module *p* values (Figure 4K) are nominal, not adjusted across modules.

### 2.13 Deep Data‐Independent Acquisition (DIA) Proteomic Analysis

HBMECs transfected as in Section 2.2 were harvested at 48 h; one pooled sample/condition (*n* = 1/group) was analyzed by Eurofins Genomics (Kazusa, Chiba) via DIA. Proteins were extracted (75 mM Tris–HCl pH 8.0, 4% SDS, 10 mM NaCl, 2 mM EDTA, 10 mM methionine, 5 mM DTT, 10% ethanol, 95°C/15 min, sonication); concentration was measured (ProteoAnalyzer, Agilent). Samples underwent SP3 preparation (Sera‐Mag SpeedBeads, Cytiva), trypsin/Lys‐C digestion (Promega, 37°C/18 h), TCEP/2‐chloroacetamide reduction‐alkylation, and GL‐Tip SDB desalting. Peptide concentration was measured by fluorometric assay. Peptides were separated on a 75 μm × 250 mm C18 column (Vanquish Neo, 44‐min gradient, 50°C) and analyzed on an Orbitrap Exploris 480 (positive ESI). DIA used a full MS1 scan (m/z 500–740, resolution 30,000) and 30 MS2 windows of 8.0 Th (m/z 200–1800, resolution 30,000, NCE 26%). Data were searched with DIA‐NN against a predicted spectral library from human UniProtKB/Swiss‐Prot (UP000005640; trypsin, ≤ 1 missed cleavage, N‐terminal Met excision, fixed Cys carbamidomethylation, peptide length 7–45, precursor charge 2–4). MS1/MS2 accuracy was 8 ppm; precursor/protein FDR were controlled at 1%; match‐between‐runs, QuantUMS quantification and retention‐time‐dependent normalization were enabled. With one pooled sample/group, protein‐level changes are reported as magnitudes without a *p* value.

### 2.14 Animals

Male WT C57BL/6J and SNCA‐knockout (KO, C57BL/6 background, Jackson Laboratory) mice were housed at 20°C–22°C, 12‐h light/dark, with free access to standard diet (Oriental Yeast, Osaka). Protocols were approved by the Ethical Committee for Animal Experiments of the University of Osaka Graduate School of Medicine (04‐093‐003) per NIH guidelines. Two ages of WT and SNCA KO mice were studied: young adult (9 weeks, *n* = 6/genotype/age) and mature adult (12 months, *n* = 6/genotype/age). To define the barrier phenotype of normal aging, young (8‐week) and aged (80‐week) male WT mice were compared (*n* = 7/group).

### 2.15 BBB Permeability Assay in Animals

BBB permeability was assessed by FITC‐dextran extravasation (adapted from Natarajan et al. 2017). Anesthetized mice were perfused via the left ventricle with 1 mL D‐PBS(−) then 40‐kDa FITC‐dextran (10 mg/mL, 1 mL/min, 2 mL total) while draining via a right‐atrial incision. Brains were fixed (4% PFA, 3 days, 4°C), cryoprotected (glycerol), frozen in 2‐methylbutane, and sectioned coronally (50 μm). All images were processed with a single blinded ImageJ macro (fixed threshold, fixed sections/fields, no manual ROI). High‐intensity FITC‐dextran signal was segmented as the vessel mask; residual signal outside this mask was scored as parenchymal extravasation and normalized to vessel area per field (cortex, hippocampus, striatum). The vessel mask derived from the tracer channel itself, not CD31/isolectin B4. Twelve‐month cohort sections had degraded after > 8 months storage and could not be re‐stained for a vessel marker.

### 2.16 Animal Brain Tissue Analysis

Sections were equilibrated at 4°C, washed thrice in PBST (0.05% Tween‐20), and antigen‐retrieved (pH 9, 10× concentrate; Dako/Agilent; Cat. No. S2367, 90°C/20 min). Residual aldehydes were quenched with 100 mM glycine, permeabilized (0.1% Triton X‐100), and blocked (5% goat serum, 1% BSA). Primary antibodies were applied for 48 h/4°C, secondary for 1–2 h/room temperature, and nuclei counterstained with DAPI. CD31 was co‐stained with occludin, ZO‐1, claudin‐5, or p53. Identical confocal settings were used across each experiment. Antibodies (Table S4) were diluted in a background‐reducing diluent (Dako; Cat. No. S3022). Brain lysates for immunoblotting were prepared as for cultured cells.

### 2.17 Endogenous IgG Extravasation Immunofluorescence and Quantification

Endogenous plasma‐derived IgG extravasation provided a tracer‐independent leakage index. Sections (Sections 2.15, 2.16) were labeled with anti‐CD31 and Alexa Fluor 647‐conjugated anti‐mouse IgG (Table S4). FLUOVIEW FV4000 images (40× objective) used identical IgG‐channel settings across genotypes and ages. A custom, automated ImageJ/FIJI macro (no manual ROI) analyzed 9‐week and 12‐month WT/SNCA KO and 8‐ and 80‐week WT mice.

The CD31 channel was smoothed (Gaussian *σ* = 1 px) and Otsu‐thresholded; objects < 5 μm^2^ were discarded and lumens filled. The mask was expanded by 10 μm to define a perivascular annulus, with distal parenchyma beyond. Annular mean IgG intensity, excluding the CD31‐positive mask, was normalized to control means (WT or young WT).

### 2.18 Isolation of Brain Microvessel and Parenchymal Fractions

Microvessel and parenchymal fractions were separated by dextran density centrifugation (capillary depletion), on ice/4°C throughout. Tissue was homogenized (4 vol HEPES‐buffered saline, pH 7.4; 20 Dounce strokes); an equal volume of ice‐cold 26% dextran (64–76 kDa) was added and mixed, then centrifuged (15,800 × g, 10 min, 4°C), yielding a parenchymal supernatant and a microvessel pellet (clarified at 1000–3000 × g if turbid). The pellet was washed 1–2× by re‐centrifugation to remove residual dextran. Separation efficiency was verified in each cohort by CD31 immunoblotting, enriched in microvessels and lowest in parenchyma (Figures S9 and S10). Both fractions were analyzed by Western blotting (Section 2.6).

### 2.19 Statistical Analysis

Values are mean ± SEM. Two‐group comparisons used unpaired two‐tailed Student's *t*‐test; two‐factor comparisons used two‐way ANOVA or mixed‐effects models, with Šídák's or Tukey's multiple‐comparison tests as indicated (GraphPad Prism 10). Significance was set at *p* < 0.05. For in vitro data, *n* denotes technical replicates unless independent experiments (*N*) are stated, in which replicate values were averaged per experiment; for in vivo data, *n* denotes individual animals. Image‐based quantification was blinded.

## 3 Results

### 3.1 Replicative Senescence Downregulates SNCA in HBMECs

To model brain endothelial aging, we serially passaged primary HBMECs until late‐passage cultures showed enlarged morphology and growth arrest. Replicative senescence was indicated by increased CDKN2A (p16INK4a) and CDKN1A (p21), together with enhanced SA‐β‐gal staining (Figure 1A,B). Consistent with our previous findings in large‐vessel endothelial cells (Takami et al. 2022), senescent HBMECs showed markedly reduced SNCA, with mRNA and protein decreasing by 48.4% and 41.2%, respectively, versus early‐passage controls (Figure 1B,C). Among the principal cells of the neurovascular unit, endogenous SNCA was readily detected in HBMECs but not in astrocytes or pericytes under equal loading (Figure S5).

**FIGURE 1:**
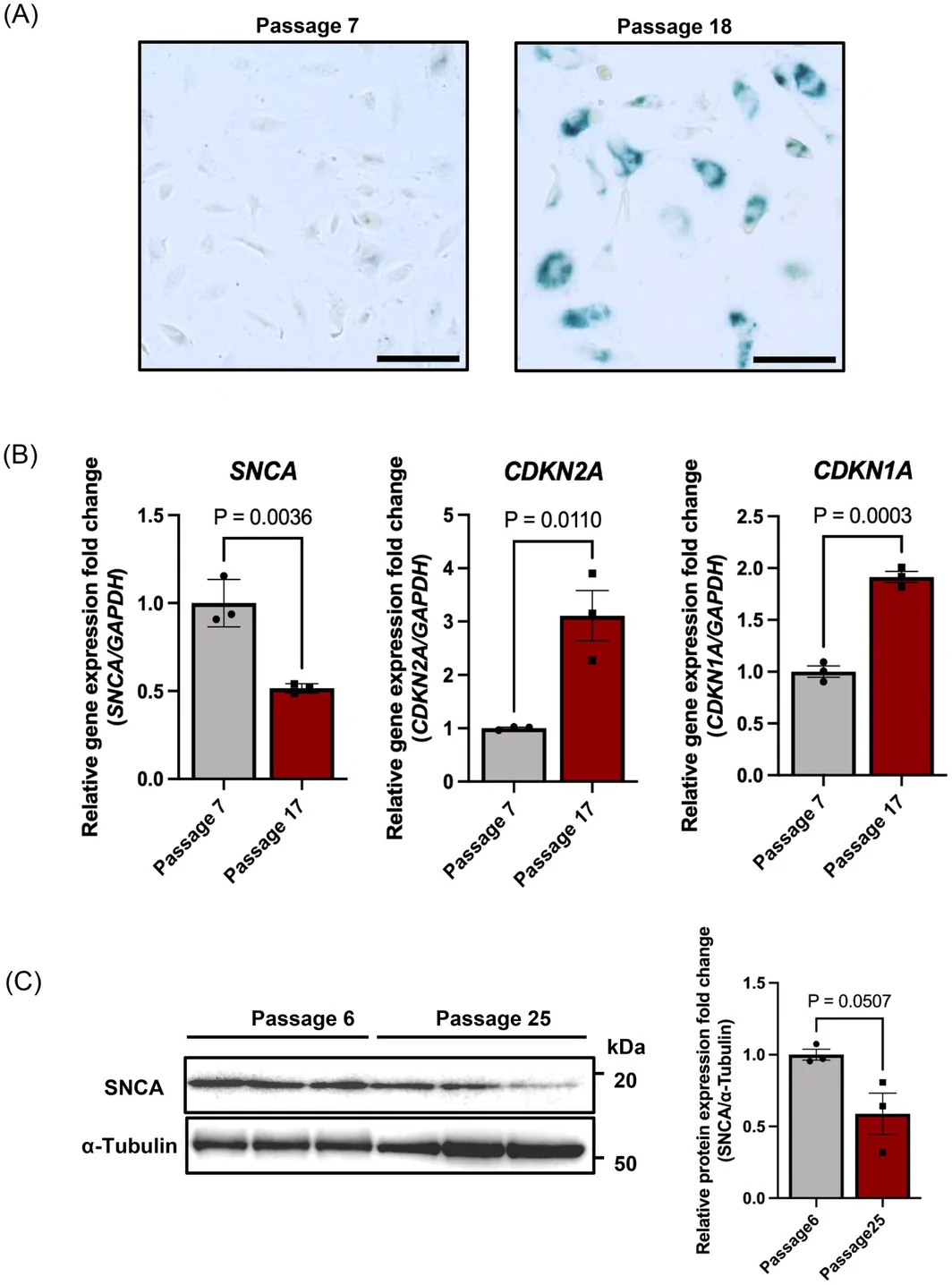
Replicative senescence downregulates SNCA in human brain microvascular endothelial cells. (A) SA‐β‐gal staining in young (passage 7) and senescent (passage 18) HBMECs (scale bar, 100 μm; *n* = 6/group). (B) qRT‐PCR of SNCA, CDKN2A (p16^INK4a^), and CDKN1A (p21) mRNA in young (passage 7) and senescent (passage 17) HBMECs, markers of replicative senescence, normalized to GAPDH (*n* = 3/group). (C) Western blot of SNCA in young (passage 6) and senescent (passage 25) HBMECs; α‐tubulin loading control (left), with quantification (right; *n* = 3/group). Data are mean ± SEM; symbols indicate independent technical replicates. Significance: Unpaired two‐tailed Student's *t*‐test.

### 3.2 siRNA‐Mediated SNCA Silencing Induced a Senescence‐Like Phenotype and Impaired Endothelial Homeostasis in HBMECs

To test whether reduced SNCA was sufficient to drive this phenotype, we silenced SNCA using siRNA. SNCA depletion increased SA‐β‐gal staining and activity (Figure 2A,B), with increased CDKN2A (p16) and TP53 (p53) transcripts, while CDKN1A (p21) did not increase (Figure 2C). SNCA knockdown also suppressed homeostatic pathways central to vascular longevity: SIRT1 decreased by 65.4% and eNOS by 33.5% (Figure 2D), and glypican‐1 (GPC1), which promotes eNOS signaling and endothelial growth (Bartosch et al. 2021; Colin‐Pierre et al. 2021; Ebong et al. 2014), decreased by 49.6% (Figure 2E). Thus, SNCA loss shifts brain endothelial cells from a homeostatic toward a dysfunctional senescence‐like state.

**FIGURE 2:**
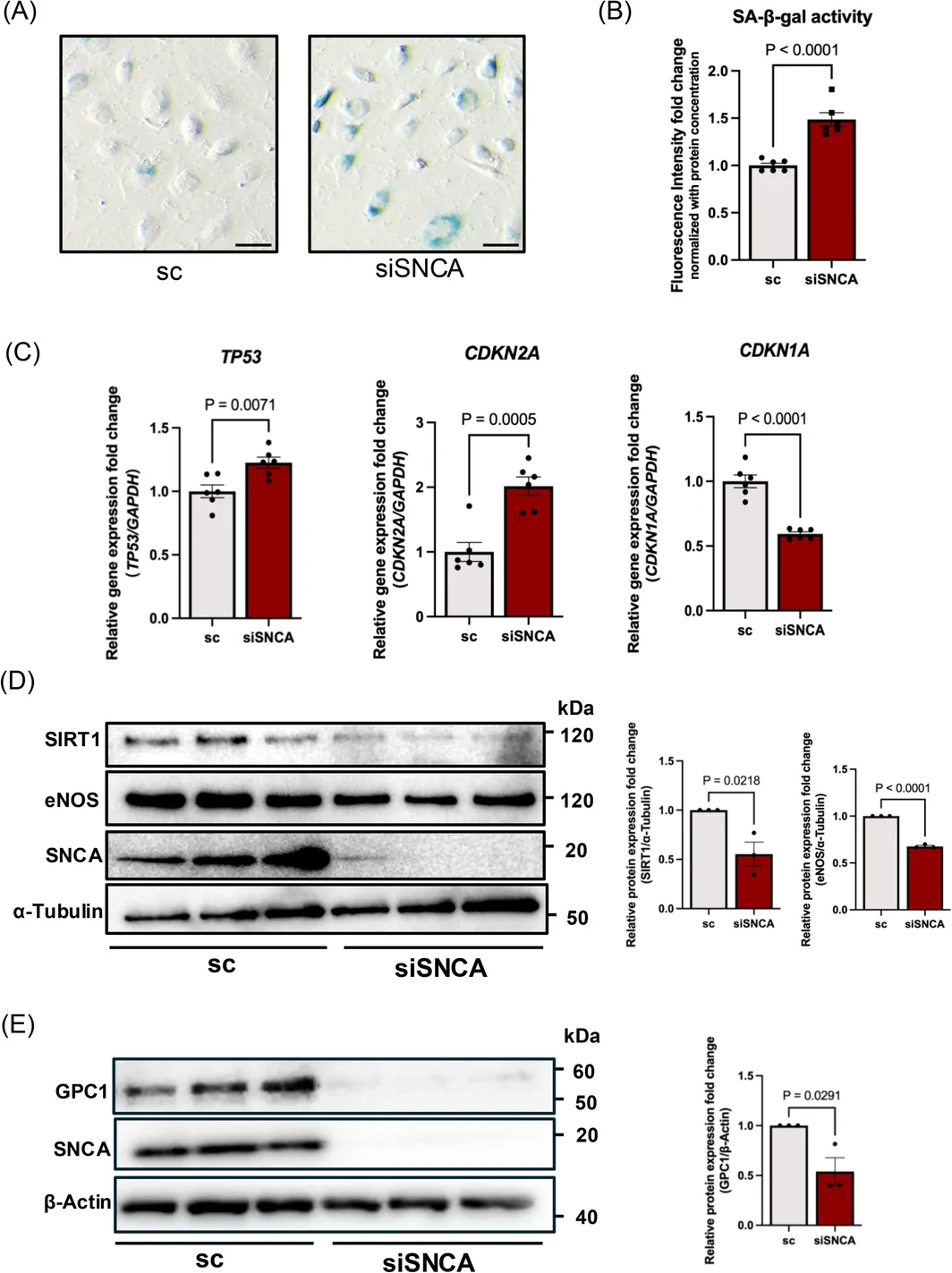
siRNA‐mediated SNCA silencing induced a senescence‐like phenotype and impaired endothelial homeostasis in HBMECs. (A) SA‐β‐gal staining in scramble (sc)‐ or SNCA siRNA (siSNCA)‐transfected HBMECs (scale bar, 100 μm; *n* = 6/group). (B) SA‐β‐gal activity, normalized to total protein (*n* = 6/group). (C) qRT‐PCR of TP53 (p53), CDKN2A (p16), and CDKN1A (p21) mRNA, normalized to GAPDH (*n* = 6/group). (D) Western blot of SIRT1, eNOS, and SNCA (left; α‐tubulin reference), with fold‐change quantification from three independent experiments (*N* = 3; right). (E) Western blot of GPC1 and SNCA (left; β‐Actin reference), with quantification (*N* = 3; right). Bars are mean ± SEM; for (B, C), symbols indicate technical replicates; for D–E, means of independent experiments. Significance: Unpaired two‐tailed *t*‐test. HBMECs were used between passages 5–9.

### 3.3 Endothelial SNCA Depletion Impairs Proliferation and Migration

SNCA knockdown reduced HBMEC viability/proliferation (MTS assay; Figure 3A), decreased transwell migration by 26.4% (Figure 3B), and delayed scratch‐wound closure significantly by 48 h (Figure 3C), indicating compromised reparative competence needed to restore monolayer integrity after injury.

**FIGURE 3:**
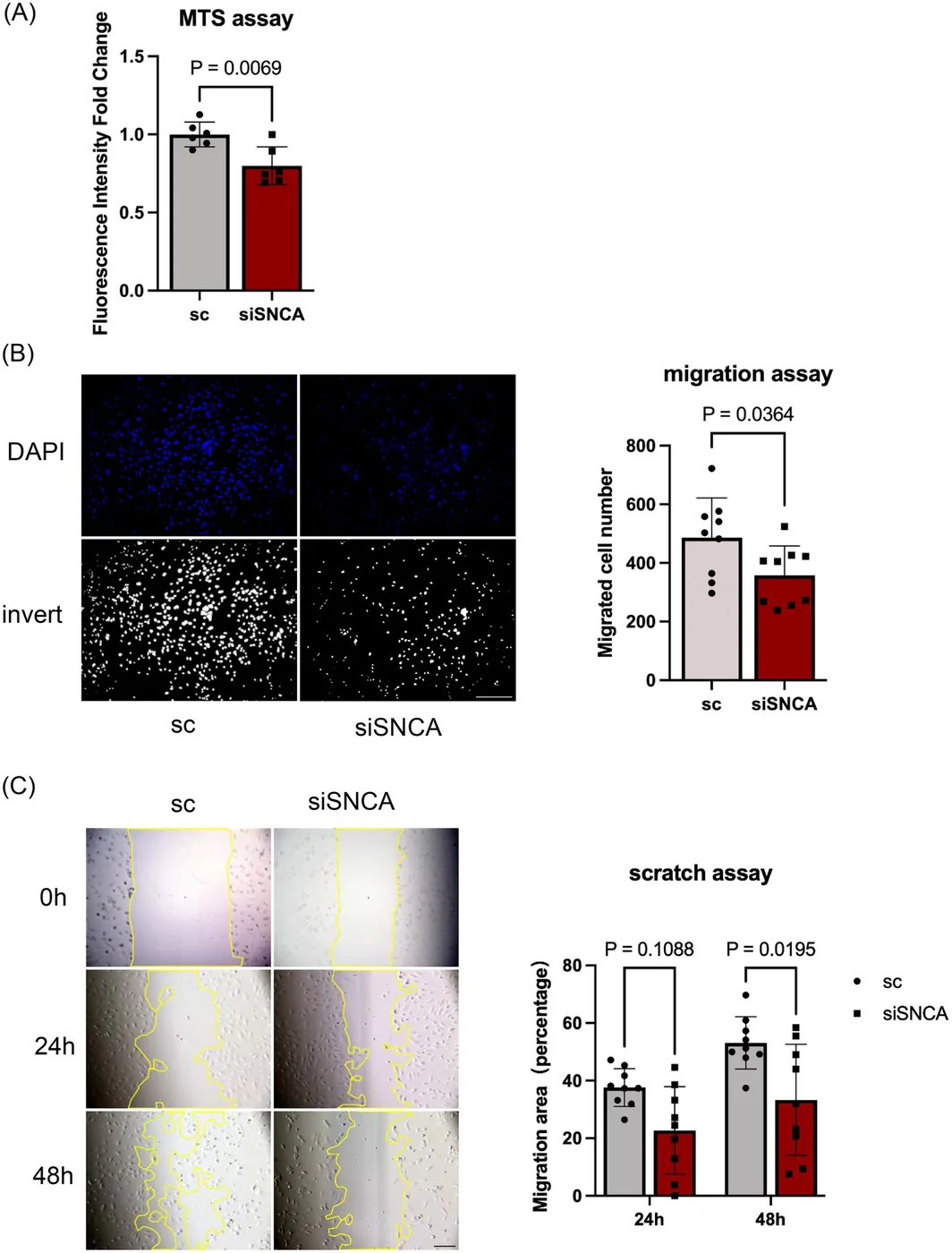
Endothelial SNCA depletion impairs proliferation and migration. (A) MTS assay of sc‐ or siSNCA‐transfected HBMECs (*n* = 6/group). (B) Transwell migration assay; representative DAPI‐stained images (scale bar, 250 μm; *n* = 9/group), with quantification (right). (C) Scratch wound‐healing assay at 0, 24, and 48 h (wound borders outlined in yellow), with quantification of migrated area (right; scale bar, 250 μm; *n* = 9/group). Bars are mean ± SEM; symbols indicate technical replicates. HBMECs were used between passages 5–9. Significance: Unpaired two‐tailed Student's *t*‐test.

### 3.4 SNCA Knockdown Disrupts Junctional Integrity and Increases Permeability

Immunoblotting showed that SNCA knockdown reduced ZO‐1 and occludin, while claudin‐5 was unchanged (Figure 4A,G). At the transcript level, OCLN (occludin) decreased whereas TJP1 (ZO‐1) and CLDN5 (claudin‐5) increased (Figure 4B), indicating the junctional phenotype is not explained by simple transcriptional suppression; occludin showed the most consistent change. Immunofluorescence revealed reduced junctional fluorescence intensity and continuity of occludin, increased heterogeneity, and elevated junctional‐to‐cytoplasmic intensity ratio (JCIR), indicating redistribution into the cytoplasm (Figure 4H), consistent with patterns reported in senescent (Y. Yamazaki et al. 2016) and oxidatively stressed (Lum and Roebuck 2001) endothelium. These changes were accompanied by increased FITC‐dextran permeability (Figure 4C), showing that SNCA loss is sufficient to destabilize junctions and impair barrier function.

**FIGURE 4:**
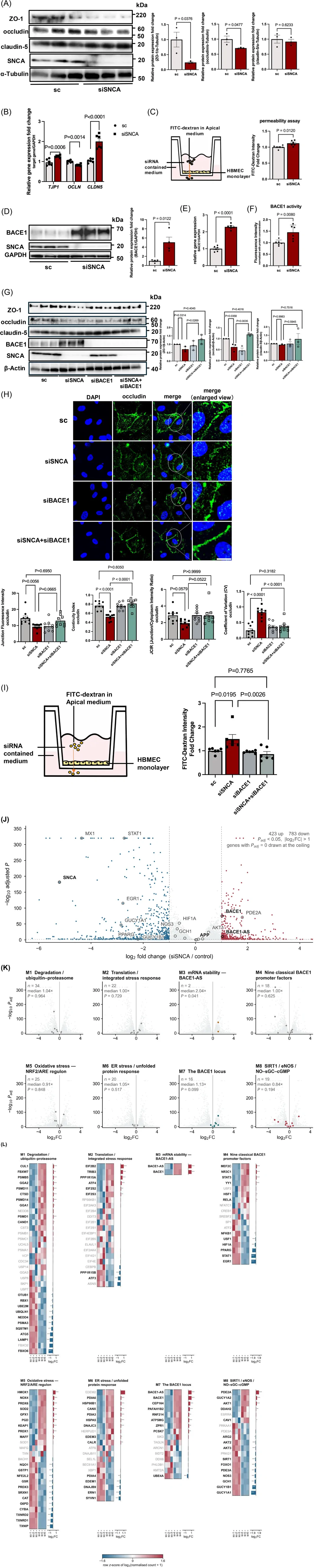
SNCA loss disrupts tight junction organization and increases BBB permeability via BACE1 upregulation. (A) Immunoblot of ZO‐1, occludin, claudin‐5, and SNCA in sc‐ or siSNCA‐transfected HBMECs (left; α‐tubulin loading control), with quantification (right; *n* = 3/condition). (B) qRT‐PCR of TJP1, OCLN, and CLDN5, normalized to GAPDH (*n* = 6/group). (C) Transwell FITC‐dextran (40 kDa) permeability quantification (*n* = 6/group). (D) Western blot of BACE1 and SNCA (left; GAPDH loading control), with quantification (right; *n* = 3/condition). (E) qRT‐PCR of BACE1 mRNA (*n* = 6/group). (F) BACE1 activity relative to scramble control (*n* = 6/group). (G) Western blot of ZO‐1, occludin, claudin‐5, BACE1, and SNCA in sc, siSNCA, siBACE1, or siSNCA + siBACE1 conditions (left; β‐actin loading control), with quantification (right; *n* = 3 independent experiments/condition). (H) Immunofluorescence of occludin under the four conditions above (scale bar, 10 μm; *n* = 9/group), with junctional fluorescence intensity, continuity index, junctional‐to‐cytoplasmic intensity ratio (JCIR), and coefficient of variation (CV) quantified below; lower continuity index/JCIR with higher CV indicate discontinuity and fragmentation. (I) Transwell FITC‐dextran permeability under the four conditions (*n* = 6/group). (J) Volcano plot of the HBMEC transcriptome after SNCA silencing (siSNCA vs. sc, *n* = 3/group; dataset quality in Figure S3); dashed lines mark significance thresholds (*p*_adj_ < 0.05, |log2FC| > 1); downregulated (blue, 783 genes) and upregulated (red, 423 genes) transcripts are colored, with selected transcripts circled (SNCA, BACE1, BACE1‐AS, APP, NOS3, AKT1, HIF1A, GCH1, GUCY1A1, GUCY1B1, PDE2A, PPARG, EGR1, STAT1, MX1). (K) The same plot with each of the eight predefined mechanism modules highlighted: M1, ubiquitin–proteasome degradation; M2, translation/integrated stress response; M3, BACE1‐AS mRNA stability; M4, nine classical BACE1 promoter factors; M5, oxidative stress/NRF2–ARE; M6, ER stress/unfolded protein response; M7, BACE1 locus; M8, SIRT1/eNOS/NO–sGC–cGMP signaling, showing genes tested (*n*), module median fold change, and *p* value. (L) Heatmaps of M1–M8 member genes; color encodes row z‐score of log2 (normalized count +1) per replicate (sc‐1–3, si‐4–6), with log2 fold change per gene; black names were detected/tested, gray names did not meet criteria. Asterisks denote adjusted *p* value (*p_adj_ < 0.05, **p_adj_ < 0.01, ***p_adj_ < 0.001). Bars are mean ± SEM; in (A–F, H–I) symbols indicate technical replicates; in (G) means of independent experiments. HBMECs were used between passages 5–9. Significance: Unpaired two‐tailed Student's *t*‐test (A–F); two‐way ANOVA/mixed‐effects model with SNCA and BACE1 knockdown as fixed factors, testing the SNCA × BACE1 interaction, with Šídák‐corrected comparisons (G–I).

### 3.5 Endothelial BACE1 Is Upregulated by SNCA Loss and Mediates Tight Junction Failure

Given links between endothelial BACE1, NO‐sensitive dysfunction, and occludin cleavage in cerebral small vessels (Katusic et al. 2024; Zhou et al. 2022), we examined BACE1 downstream of SNCA depletion. SNCA knockdown increased BACE1 protein 5.06‐fold, mRNA 2.29‐fold, and activity 1.45‐fold (Figure 4D–F). Co‐silencing BACE1 with SNCA restored ZO‐1 and occludin, improved occludin organization, and normalized permeability (Figure 4G–I), identifying BACE1 upregulation as a key downstream effector of the SNCA‐loss junctional phenotype.

Because BACE1 has been reported to cleave occludin and generate C‐terminal fragments (Zhou et al. 2022), we examined whether reduced full‐length occludin was accompanied by detectable cleavage products. A C‐terminal occludin antibody confirmed the reduction in full‐length occludin after SNCA knockdown; the immunoblot covered a broad molecular‐weight range, but no discrete lower‐molecular‐weight fragments were detected (Figure S2A). BACE1 overexpression similarly reduced full‐length occludin without detectable fragments (Figure S2C). SNCA knockdown decreased occludin mRNA while increasing BACE1 (Figure S2B), whereas BACE1 overexpression increased occludin mRNA (Figure S2D); thus, although both perturbations reduced full‐length occludin protein, their effects on occludin mRNA differed.

Since SNCA silencing reduced eNOS, we investigated impaired NO signaling as a contributor to BACE1 induction. The NO donor SNAP partially attenuated BACE1 upregulation after SNCA silencing (Figure S1), supporting reduced NO bioavailability as a contributor, though unlikely the sole mechanism.

### 3.6 Transcriptomic and Proteomic Profiling Implicates Disruption of the NO–sGC–cGMP Axis in BACE1 Induction

To determine how SNCA loss induces BACE1, we profiled the transcriptome and proteome of scramble‐control and SNCA‐silenced HBMECs (*n* = 3/group) and systematically tested eight predefined mechanisms (Figures 4J–L and S4). PCA separated the three scramble from three SNCA‐silenced libraries along PC1 (70.1% of variance; Figure S3).

BACE1 mRNA rose 2.03‐fold by RNA sequencing versus 5.06‐fold for protein (Figure 4D); because protein exceeded transcript induction, we examined both layers. APP mRNA was unchanged (Figure 4J), indicating a BACE1‐selective response. The ubiquitin–proteasome machinery, GGA3, integrated stress response, oxidative‐stress, and unfolded‐protein‐response arms were unchanged, and none of the nine classical BACE1 promoter factors was activated by its own target‐gene output. BACE1‐AS rose in parallel with BACE1, but genome‐wide sense–antisense concordance is common in this dataset, so this alone does not establish a stabilizing role (Figure 4K,L). BACE1 itself lay at the 99.1st percentile of expressed genes, while its 25 genomic neighbors (±1.5 Mb) changed only 1.09‐fold median, indicating locus‐specific rather than regional induction.

Across the 8241 genes quantified on both platforms, transcript–protein correlation was modest (*r* = 0.68; Figure S4A); restricting to the 331 of 1206 significantly changed transcripts also quantified in the proteome raised this to *r* = 0.88, with 96% concordant direction. At the module level, six of eight predefined mechanisms showed near‐zero median fold change on both platforms (Figure S4B), an orthogonal line of evidence against their involvement.

What changed coherently was the NO–soluble guanylyl cyclase (sGC)–cGMP axis at three sequential points: NO synthesis (NOS3, 0.62‐fold; GCH1, 0.59‐fold), NO reception (GUCY1A1, 0.14‐fold; GUCY1B1, 0.22‐fold), and cGMP removal (PDE2A, 3.47‐fold), with SIRT1 reduced (0.76‐fold). All 12 significant, proteome‐quantifiable members of this axis changed concordantly across platforms (*r* = 0.918; Figure S4A), exceeding the genome‐wide concordance (*r* = 0.88).

### 3.7 SNCA Deficiency Sensitizes the Endothelial Barrier to Amyloid‐β42 (Aβ42) Oligomers

Given BACE1's role in both junction disruption and Aβ generation from APP, and its frequent coexistence with amyloid stress in aging/Alzheimer's pathology, we asked whether barrier vulnerability in SNCA‐deficient endothelium is exacerbated by soluble amyloid. SNCA knockdown alone reduced occludin and ZO‐1; added Aβ42 oligomer (10 μM) caused greater loss of occludin, ZO‐1, and claudin‐5 than either insult alone (Figure 5A). Permeability assays corroborated this: Aβ42 modestly increased flux in control monolayers but substantially more in SNCA‐deficient monolayers (Figure 5B), indicating SNCA loss both impairs baseline integrity and lowers the threshold for Aβ‐mediated injury.

**FIGURE 5:**
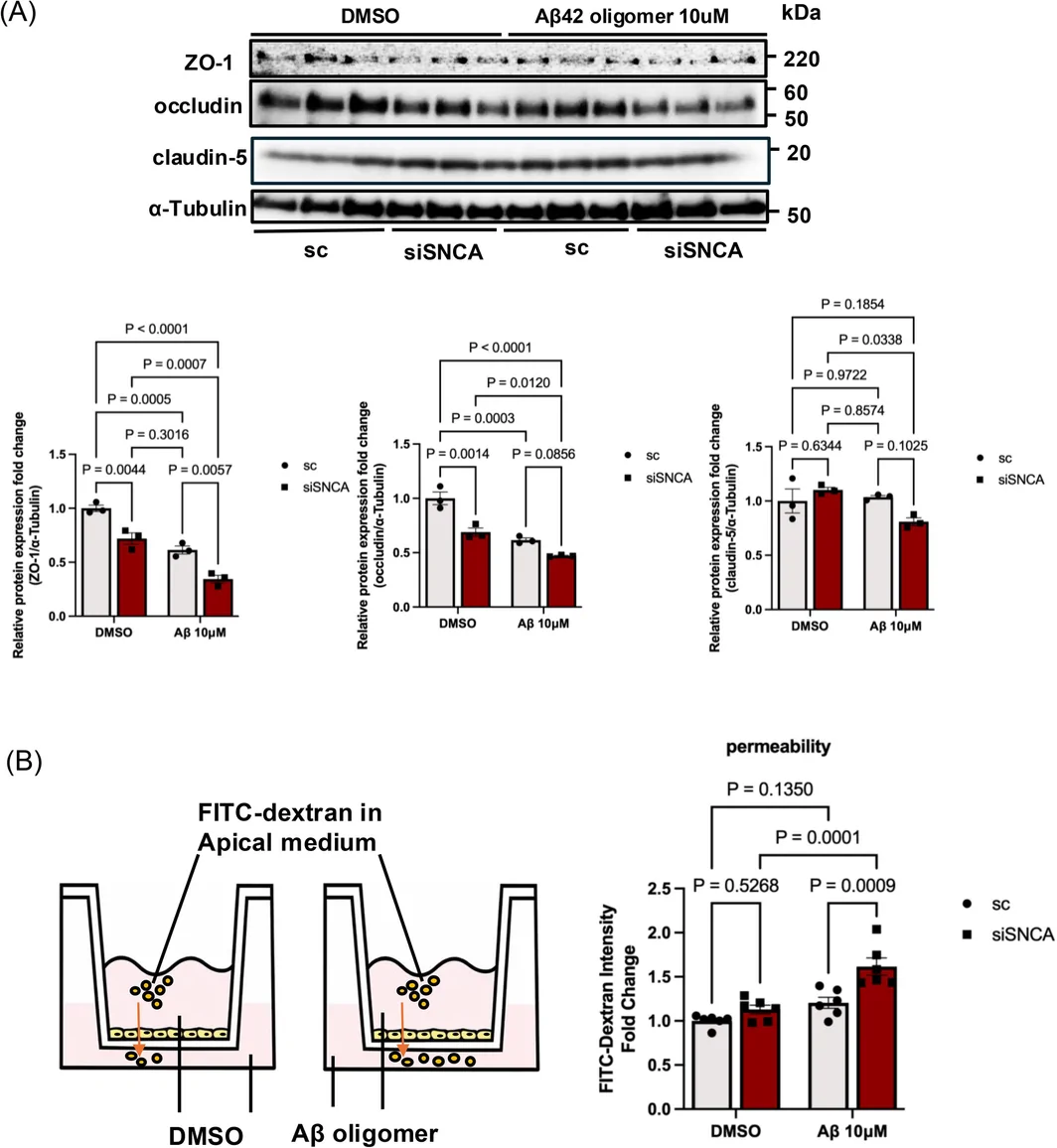
SNCA deficiency sensitizes the endothelial barrier to Aβ42 oligomers. (A) Western blot of ZO‐1, occludin, claudin‐5, and SNCA in sc‐ or siSNCA‐transfected HBMECs treated with vehicle (DMSO) or Aβ42 oligomers (10 μM) for 24 h (upper; α‐tubulin loading control), with quantification below (*n* = 3/condition). (B) Transwell FITC‐dextran permeability under vehicle or Aβ42 (*n* = 6/group). Bars are mean ± SEM; symbols indicate technical replicates. HBMECs were used between passages 5–8. Significance: Two‐way ANOVA with Tukey's multiple‐comparisons test.

To test lower concentrations, the experiment was repeated at 1 and 5 μM. At 1 μM, Aβ42 did not significantly alter junction proteins or flux in control monolayers; in SNCA‐silenced monolayers, ZO‐1 decreased and flux increased numerically, but within‐genotype comparisons were not significant (*p* = 0.0577, *p* = 0.0705). Although permeability was higher in Aβ42‐challenged siSNCA than challenged control cells (*p* = 0.0032), baseline permeability was also higher after SNCA silencing (*p* = 0.0360), and neither ZO‐1 nor permeability showed a significant SNCA × Aβ42 interaction (*p* = 0.1805, *p* = 0.4651); thus the 1 μM data indicate trends but do not establish sensitization (Figure S7). At 5 μM, Aβ42 significantly reduced occludin and increased flux in control monolayers; SNCA silencing further exacerbated flux, with only a non‐significant downward trend in occludin, and claudin‐5 unchanged (Figure S8). Barrier injury was fully established at 5 μM, whereas at 1 μM the direction was consistent with sensitization without statistical confirmation.

### 3.8 In Vivo Global SNCA Knockout Disrupts Cerebrovascular Endothelial Junction Integrity and Increases Multiregional BBB Leakage

Global SNCA KO mice showed significantly increased FITC‐dextran extravasation in cortex and hippocampus, with concordant changes in striatum (Figure 6A). Cortical CD31‐positive microvessels showed reduced occludin, ZO‐1, and claudin‐5 (Figure 6C), with concordant whole‐brain immunoblotting (Figure 6D). Increased endogenous perivascular IgG in all three regions of 12‐month‐old SNCA KO versus WT mice independently corroborated leakage (Figure 6B).

**FIGURE 6:**
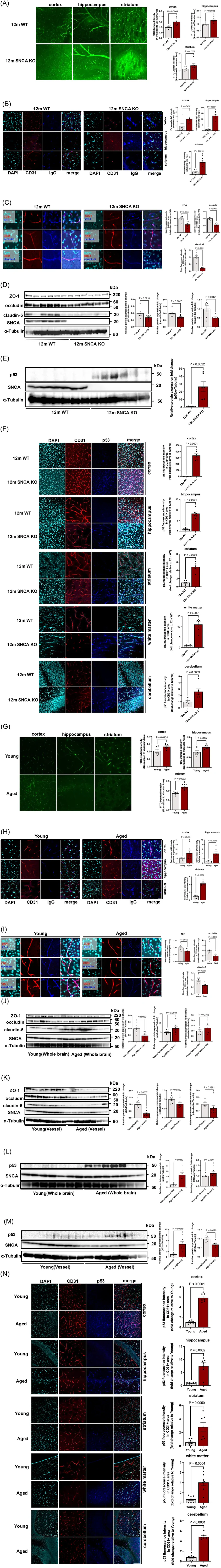
Global SNCA knockout disrupts cerebrovascular endothelial junctional integrity and enhances BBB leakage and endothelial p53 signaling in vivo, a phenotype reproduced in aged wild‐type mice. (A) FITC‐dextran (40 kDa) extravasation in cortex, hippocampus, and striatum of 12‐month‐old WT and SNCA KO mice (scale bar, 100 μm; *n* = 6/group); signal outside the tracer‐derived vessel mask was scored as parenchymal leakage and normalized to vessel area per field via a blinded ImageJ macro. (B) Immunofluorescence of DAPI, CD31, endogenous IgG, and merge in the same regions/mice (*n* = 6/group); mean IgG intensity within the 10‐μm perivascular annulus (CD31‐mask excluded), relative to the WT mean. (C) Immunofluorescence of DAPI, CD31, and the indicated tight junction protein in cortical microvessels (*n* = 6/group), with mean gray‐value quantification within CD31‐positive areas. (D) Immunoblot of ZO‐1, occludin, claudin‐5, and SNCA in whole‐brain lysates (α‐tubulin control; *n* = 6), with quantification. (E) Western blot of p53 and SNCA in whole‐brain lysates (*n* = 6), with p53 quantification. (F) Immunofluorescence of DAPI, CD31, and p53 in cortex, hippocampus, striatum, white matter, and cerebellum (*n* = 6/group), with p53 quantification within CD31‐positive areas. (G) FITC‐dextran BBB permeability in young and aged WT mice, quantified as in (A). (H) Immunofluorescence of DAPI, CD31, endogenous IgG, and merge in young and aged mice, with perivascular IgG quantification relative to the young mean. (I) Immunofluorescence of ZO‐1, occludin, and claudin‐5 with CD31 and DAPI in cortex of young and aged mice, quantified as fold change relative to young mice. (J, K) Western blot of ZO‐1, occludin, claudin‐5, and SNCA in whole‐brain lysates (J) and isolated brain microvessel fractions (K; enrichment in Figure S9) of young and aged mice, with quantification. (L, M) Western blot of p53 and SNCA in whole‐brain lysates (L) and isolated microvessel fractions (M) of young and aged mice, with quantification. (N) Immunofluorescence of DAPI, CD31, p53, and merge in cortex, hippocampus, striatum, white matter, and cerebellum of young and aged mice, with p53 quantification within CD31‐positive areas, as fold change relative to young mice. Bars are mean ± SEM; symbols indicate individual biological replicates. Twelve‐month‐old WT and SNCA KO mice were used for panels (A–F); young (8‐week) and aged (80‐week) male WT mice (*n* = 7/group) for panels (G–N). Significance: Unpaired two‐tailed Student's *t*‐test (A–N).

We next asked when this phenotype first appears. In 9‐week‐old mice, SNCA KO animals already showed increased perivascular IgG extravasation in cortex, hippocampus, and striatum, with reduced ZO‐1, occludin, and claudin‐5 in cortical microvessels (Figure S6A,B). In isolated microvessel fractions, ZO‐1 was significantly reduced, with occludin and claudin‐5 changing concordantly without reaching significance, and none differed in whole‐brain lysate (Figure S6C,D), consistent with an endothelium‐restricted change diluted in whole brain. CD31 immunoblotting confirmed microvessel enrichment in both age cohorts (Figures S9 and S10). BBB leakage in SNCA‐deficient mice therefore begins in young adulthood. The same readouts changed concordantly in aged versus young WT mice, with increased FITC‐dextran/IgG extravasation and tight junction loss (Figure 6G–K); critically, SNCA itself was reduced in isolated microvessels of aged mice while unchanged in whole‐brain lysate (Figure 6J,K), showing that age‐associated endothelial SNCA decline occurs in vivo and is masked by neuronal SNCA in whole brain. Thus, the barrier phenotype of young SNCA‐null mice reproduces that of normal aging.

### 3.9 SNCA Deficiency Induces Multiregional p53 Responses Localized to CD31‐Positive Microvessels In Vivo

We next asked whether the BBB phenotype was accompanied by a cerebrovascular stress response. Whole‐brain immunoblotting showed markedly increased p53 in SNCA KO versus WT brain (Figure 6E). Double immunofluorescence for p53 and CD31 showed virtually undetectable co‐localization in WT mice, but robust p53 within CD31‐positive vessels across cortex, hippocampus, striatum, white matter, and cerebellum in SNCA KO mice (Figure 6F), with little non‐vascular staining, indicating an endothelial‐restricted stress response spanning multiple vascular territories. This response tracked with the barrier phenotype across ages: in 9‐week‐old SNCA KO mice, p53 was already increased in whole‐brain lysates and isolated microvessels, confined to CD31‐positive profiles, but significant in only three of five regions (cortex, hippocampus, white matter), extending to all five by 12 months (Figure S6E–G); an equivalent endothelial p53 induction was observed in aged versus young WT mice (Figure 6L–N).

## 4 Discussion

Our findings associate endothelial SNCA loss with BACE1‐related barrier dysfunction and increased endothelial p53 in vivo, supporting a stress‐associated microvascular BBB phenotype.

We observed a consistent decrease in SNCA mRNA and protein in replicatively senescent HBMECs, extending our previous findings in large‐vessel endothelium (Takami et al. 2022). Independent studies report inverse associations between circulating SNCA, age, and metabolic stress (Koehler et al. 2015; Rodriguez‐Araujo et al. 2015); since SNCA can be secreted (Emmanouilidou et al. 2010; Takami et al. 2022), endothelial cells may partially contribute to the circulating pool, supporting reduced endothelial SNCA as part of the stress‐associated endothelial transition accompanying senescence and BBB vulnerability.

Based on this decline, siSNCA induced a senescence‐like phenotype marked by increased SA‐β‐gal, CDKN2A (p16) and TP53 (p53) induction, and reduced SIRT1, eNOS, and GPC1, alongside reduced proliferative/migratory competence relevant to barrier maintenance, since endothelial regeneration is required for vascular repair (Evans et al. 2021) and BBB integrity depends on dynamic tight‐junction regulation (Luissint et al. 2012; Stamatovic et al. 2016). These findings support the interpretation that SNCA deficiency weakens endothelial monolayer resilience and predisposes the barrier to paracellular dysfunction.

Our data indicate an inverse relationship between endothelial protective pathways and BACE1 induction. Loss of SIRT1 and eNOS is a recognized feature of senescent/dysfunctional endothelium (Matsushita et al. 2001; Mattagajasingh et al. 2007); reduced NO signaling has been linked to increased BACE1 (Austin et al. 2010), while NO supplementation suppresses APP/BACE1 in the cerebral microcirculation (Austin et al. 2013), consistent with the partial SNAP attenuation observed here (Figure S1). This supports impaired NO signaling as mechanistically relevant; the transcriptomic/proteomic analyzes below explain why rescue was incomplete—the pathway is interrupted not only at NO synthesis but also at NO reception and cGMP hydrolysis. Replicative senescence itself increases BACE1 and amyloidogenic APP processing in HBMECs (Sun et al. 2018), and reduced GPC1 is mechanistically relevant since GPC1 supports eNOS signaling (Bartosch et al. 2021; Ebong et al. 2014), with BACE1 inactivation increasing both GPC1 and eNOS in cerebral microvessels (He et al. 2022); excessive endothelial BACE1 also reduces eNOS activity and disrupts occludin‐dependent barrier integrity in hypertensive human brain microvessels (He et al. 2022; Katusic et al. 2024). Collectively, SNCA loss promotes a dysfunctional senescence‐like state partly by suppressing SIRT1‐eNOS‐NO and GPC1‐dependent signaling, favoring BACE1 upregulation.

Building on the transcriptomic/proteomic data (Section 3.6), multi‐omic profiling argued against most classically proposed BACE1‐raising mechanisms—proteasomal/lysosomal degradation, integrated‐stress‐response translation, and the nine canonical promoter factors—and instead identified a locus‐specific transcriptional increase in BACE1 coupled with a coordinated, three‐point interruption of the NO–sGC–cGMP axis (synthesis, reception, cGMP clearance). This explains the incomplete SNAP rescue: exogenous NO reaches a system where its receptor, soluble guanylyl cyclase, is itself reduced, while cGMP is hydrolyzed faster by PDE2A, so restoring only the first of three interrupted steps would not normalize BACE1 fully.

BACE1 thus provides a plausible mechanistic link between SNCA deficiency and tight junction failure. BACE1 can directly cleave occludin and compromise barrier integrity in small cerebral vessels (Katusic et al. 2024; Zhou et al. 2022). In our model, full‐length occludin fell after both SNCA silencing and BACE1 overexpression, without a resolved cleavage fragment in either condition (Figure S2), so cleavage remains the most parsimonious explanation rather than a direct demonstration. SNCA silencing lowered occludin as both protein and OCLN mRNA (Figure 4A,B; Figure S2A,B), whereas BACE1 overexpression lowered occludin protein while OCLN mRNA rose slightly (Figure S2C,D), consistent with a compensatory transcriptional response; elevated BACE1 therefore accounts for part, not all, of the occludin loss after SNCA silencing. This axis has also been implicated in human cerebral amyloid angiopathy (Cheng et al. 2014). Most importantly, BACE1 co‐silencing rescued occludin and ZO‐1, placing BACE1 functionally downstream of SNCA loss as a critical executor of barrier damage rather than a passive bystander.

Occludin was the most consistently affected junctional component, whereas ZO‐1 changes were modest and claudin‐5 protein was comparatively preserved despite increased CLDN5 transcript—a pattern more consistent with post‐transcriptional injury than purely transcriptional suppression. This may explain the apparent divergence between acute HBMEC depletion and chronic in vivo deficiency: within the intact neurovascular unit, endothelial cells face astrocytic, pericytic, inflammatory, and hemodynamic signals absent in monoculture, so sustained deficiency may require broader junctional remodeling.

Our data further indicate that loss of physiological endothelial SNCA increases BBB susceptibility to amyloid stress, distinct from prior work linking injury to aggregated/fibrillar SNCA or synucleinopathy‐associated BBB alterations (Hourfar et al. 2023; Kuan et al. 2016; Lau et al. 2024). Concentration‐response experiments did not establish sensitization at 1 μM, while at 5 μM Aβ42 significantly increased permeability in control monolayers with a further significant increase after SNCA silencing (Figures S7 and S8); the Aβ42 concentrations used (1–10 μM, monomer‐equivalent) exceed free Aβ42 in human brain interstitial fluid (Herukka et al. 2015) and were chosen to compress a chronic, locally concentrated in vivo exposure into an acute challenge, since brain endothelium in Alzheimer's disease is exposed to a locally concentrated amyloid pool, with water‐soluble Aβ42 in frontal cortex reaching ~1000‐fold above amyloid‐free brain (Tabaton and Piccini 2005) and many cortical microvessels carrying Aβ42 deposits (Thomas et al. 2000). These data suggest SNCA loss may lower the threshold for Aβ42‐induced injury; given progressive Aβ42 accumulation in the aging/AD brain and the role of BBB failure in amplifying neuroinflammatory injury (Katusic et al. 2024; Mogi et al. 2026), age‐related SNCA decline may contribute to amyloid‐associated barrier dysfunction. Late‐life barrier dysfunction may thus reflect not only toxic gain‐of‐function of aggregated α‐synuclein, but also insufficiency of endogenous endothelial SNCA with aging.

The senescence program accompanying SNCA deficiency appears non‐canonical: SA‐β‐gal activity and CDKN2A (p16)/TP53 (p53) rose, while CDKN1A (p21) decreased, suggesting a p53‐associated stress trajectory not dominated by the classical p53‐p21 arrest axis—plausible given that p21 is highly context‐ and time‐dependent and can dissociate from p16‐linked programs (Lau et al. 2024; Saul et al. 2025). SNCA and p53 signaling are bidirectionally linked, with SNCA modulating p53‐dependent responses and p53 transactivating the SNCA promoter (Alves Da Costa et al. 2002; Duplan et al. 2016), supporting a non‐canonical senescence‐like phenotype characterized by prominent p16 induction and a p53‐associated stress response.

The in vivo data substantially strengthen this interpretation. Global SNCA KO mice showed increased BBB permeability and loss of occludin, ZO‐1, and claudin‐5; p53/CD31 co‐localization, virtually undetectable in WT, became prominent across cortex, hippocampus, striatum, white matter, and cerebellum, remaining confined to CD31‐merged structures with little parenchymal signal, a vessel‐restricted pattern more consistent with a primarily endothelial than a diffuse neuronal stress response, linking the cultured‐HBMEC p53 response to the brain microvasculature in vivo, without excluding contributions from neurons, astrocytes, pericytes, or peripheral tissues. These data place the cerebrovascular endothelium among the principal stress sites in vivo, refining links between aging, brain endothelial senescence, and p16‐associated programs (Novo et al. 2024; Yamazaki et al. 2016) and recent evidence that senescent cerebral endothelial cells drive BBB disruption, microvascular rarefaction, and cerebral small vessel disease‐related phenotypes (Csik et al. 2025; Kawatani et al. 2025).

Two further observations refine the temporal interpretation. First, injected‐tracer leakage was reproduced by endogenous IgG extravasation in the same mice (Figure 6B), indicating the defect is not tracer‐specific. Second, the phenotype is already established in young adulthood: 9‐week‐old SNCA KO mice showed perivascular IgG extravasation, junctional protein loss, a significant ZO‐1 reduction in isolated microvessels, and endothelial p53 induction in three of five regions, extending to all five by 12 months (Figure S6), matching the pattern of aged‐versus‐young WT mice (Figure 6G–N). Removing SNCA in a young animal thus reproduces the barrier phenotype of normal aging, supporting a contributory rather than merely correlative role.

Because in vivo experiments used global SNCA KO mice, we considered which compartment likely drives this phenotype. SNCA expression was essentially endothelial‐restricted among neurovascular‐unit cells, undetectable in astrocytes or pericytes (Figure S5). Neurons express SNCA abundantly, and an indirect neuronal influence cannot be formally excluded, but several considerations argue against it as the principal driver: the SNCA–eNOS–BACE1–occludin axis, including BACE1‐rescue, is fully reconstituted in pure HBMEC monoculture absent neurons, mural cells, or flow; neurovascular‐coupling deficits described in synucleinopathy derive from α‐synuclein overexpression (gain‐of‐function) models (Biju et al. 2020; Lungu et al. 2025) and have not been reported in Snca‐null mice; Snca‐null mice are viable and fertile with only subtle synaptic changes (Abeliovich et al. 2000; Chandra et al. 2004), and even triple‐synuclein‐knockout mice retain largely normal neuronal function (Greten‐Harrison et al. 2010); and a coupling‐dependent, hypoperfusion‐driven mechanism would predict a watershed‐weighted regional pattern, whereas we observed uniform endothelial p53 induction across all five regions, more consistent with a cell‐autonomous, pan‐vascular process. A systemic contribution via SNCA‐dependent hypertension (Takami et al. 2022) likewise cannot be excluded, though hypertensive injury is typically watershed‐predominant.

These findings may be relevant to the vascular contribution to late‐life cognitive impairment, although that link remains indirect here. Much of the vascular burden underlying cognitive impairment in older adults occurs at the level of small arteries, arterioles, capillaries, and the BBB rather than large vessels (De Silva and Faraci 2020; Mogi et al. 2026), and BBB/neurovascular dysfunction is increasingly recognized as a major amplifier of dementia progression (Sweeney et al. 2018). Together with prior evidence that age‐related endothelial SNCA decline contributes to peripheral hypertension and insulin resistance (Rodriguez‐Araujo et al. 2013, 2015; Takami et al. 2022), these findings suggest SNCA may be a shared molecular substrate linking lifestyle‐related vascular risk to dementia‐related cerebrovascular injury.

Our study had several limitations. siRNA‐induced off‐target effects and transfection stress cannot be fully excluded, though BACE1 co‐silencing rescue supports biological relevance; a gain‐of‐function SNCA rescue was not performed given its aggregation‐prone toxicity. No discrete occludin cleavage fragment was detected, though BACE1 silencing and overexpression changed occludin abundance in opposite directions. In vivo experiments used systemic SNCA KO mice, so the BBB phenotype cannot be attributed exclusively to endothelial SNCA deficiency, although CD31‐merged p53 data are consistent with the cerebrovascular endothelium as a major locus of stress, since SNCA expression is essentially endothelial‐restricted among neurovascular‐unit cells (Figure S5), and several lines of evidence argue against neuronal, astrocytic, pericytic and systemic compartments as the principal driver. We did not assess cognitive/behavioral outcomes; our data support a role for SNCA deficiency in BBB dysfunction but do not establish that endothelial SNCA loss causes cognitive impairment, requiring future endothelium‐restricted deletion (e.g., AAV‐BR1; Snca^fl/fl^; Cdh5‐CreERT2) with adequately powered behavioral testing. The transcriptomic/proteomic data implicating NO–sGC–cGMP disruption (Section 3.6) are correlative and cross‐sectional, and causality with the broader phenotype could not be established. The in vivo permeability data are image‐based rather than homogenate‐based, since fixation followed tracer perfusion directly without a tracer‐free wash step.

Overall, our study supports the idea that age‐related loss of function of endogenous SNCA in brain microvascular endothelial cells results in a non‐canonical senescence‐like endothelial state characterized by prominent p16 induction, p53‐associated stress response, BACE1 upregulation, and downstream occludin‐centered junctional injury, thereby increasing the susceptibility to paracellular BBB leakage across brain regions. In particular, the newly identified pathophysiological role of SNCA in the brain microvascular endothelium suggests that SNCA may constitute a common molecular substrate linking aging, lifestyle‐related diseases, such as hypertension and diabetes, cerebral small‐vessel pathology, and the vascular components of Alzheimer's disease in older adults. In other words, an age‐related decline in endothelial SNCA level may contribute to vascular risk states and to neurovascular injury of the type associated with dementia. Endothelial SNCA therefore warrants further investigation as a potential target for preserving BBB integrity in cerebral small vessel disease and in the vascular component of Alzheimer's disease, which commonly coexist in older adults. As cognitive outcomes were not examined in the present study, this remains a hypothesis to be tested.

## Author Contributions

Z.W. and Y.T. conceived and designed the study. Z.W., Y.T., and C.W. performed the experiments and acquired data. Z.W., Y.T., C.W., and H.K. analyzed and interpreted the data. J.I., Y.G., W.L., N.Y., L.Y., D.N., and H.T. participated in the data analysis and discussion. Y. Kase supported both the execution of the RNA‐sequencing and proteomic analyzes and the interpretation of the resulting data. Z.W. and Y.T. drafted the manuscript. Y.T., K.Y., and H.K. critically revised the manuscript for intellectual content. S.T., A.H., T.T., M.S., H.N., Y. Kaneda, and K.Y. supervised the study and provided the guidelines. All authors provided final approval for the version to be published, participated sufficiently in the work to take public responsibility for appropriate portions of the content, and agreed to be accountable for all aspects of the study.

## Funding

This work was supported by the JSPS KAKENHI (Grant Number: 24K10551) (Y.T.), Eli Lilly Japan Innovation Research Grant 2024 (Y.T.) and Fujita Mind‐Brain Research & Innovation Center for Drug Generation (Fujita Mind‐BRIDGe) of Japan's Peak Research Universities (J‐PEAKS) Program (JPJS00420240019) funded by the Japan Society for the Promotion of Science (JSPS) (Y.K.).

## Conflicts of Interest

The authors declare no conflicts of interest.

## Supporting information


**Data S1:** acel70770‐sup‐0001‐Supinfo1.pdf.

**Table S1:** siRNAs used in this study.
**Table S2:** Plasmid used in this study.
**Table S3:** Primers used in this study.
**Table S4:** Antibodies used in this study.

**Figure S1:** S‐nitroso‐N‐acetyl‐DL‐penicillamine (SNAP) partially attenuates the BACE1 upregulation induced by SNCA silencing in HBMECs. Upper panel: western blot analysis of BACE1 and SNCA in human brain microvascular endothelial cells (HBMECs) transfected with scramble control siRNA (sc) or SNCA siRNA (siSNCA), with or without the nitric oxide donor SNAP (200 μM) (*n* = 3 per condition). α‐Tubulin was used as the loading control. Representative immunoblots are shown. Lower panel: corresponding densitometric analysis of the western blot shown in the upper panel. Bars represent mean ± SEM. Individual symbols indicate independent technical replicates. All HBMECs were used between passages 5 and 8. Statistical analysis was performed using two‐way ANOVA followed by Tukey's multiple‐comparisons test.
**Figure S2:** SNCA silencing and BACE1 overexpression both reduce full‐length occludin in HBMECs without a detectable cleavage fragment. (A) Left: western blot of occludin, detected with a C‐terminal antibody, in human brain microvascular endothelial cells transfected with scramble control siRNA (sc) or SNCA siRNA (siSNCA), together with BACE1, SNCA and α‐Tubulin from the same experiment (*n* = 3 per condition). The occludin membrane is shown over its full molecular weight range (20–220 kDa) so that the region below full‐length occludin can be inspected; α‐Tubulin was used as the loading control. N.S., non‐specific bands. Right: densitometric quantification of occludin normalized to α‐Tubulin and expressed as fold change relative to the scramble control. Bars represent mean ± SEM; individual symbols indicate independent technical replicates. All HBMECs were used between passages 5 and 8. Statistical analysis was performed using an unpaired two‐tailed Student's *t*‐test. (B) Quantitative reverse transcription‐PCR analysis of BACE1 (left) and OCLN (right) mRNA in sc‐ and siSNCA‐transfected HBMECs. Transcript levels were normalized to GAPDH and are expressed as fold change relative to the scramble control (*n* = 6 per group). (C) Western blot of occludin detected with the C‐terminal antibody, BACE1 and α‐Tubulin in HBMECs transfected with empty vector (EV) or a BACE1 expression plasmid (BACE1‐OE) (*n* = 3 per condition). The occludin membrane is shown over its full molecular weight range (20–220 kDa). BACE1 overexpression increased BACE1 and reduced full‐length occludin, and no discrete occludin cleavage fragment was resolved. Densitometric quantification of full‐length occludin normalized to α‐Tubulin and expressed as fold change relative to EV is shown on the right. The plasmid used is listed in Table S2. (D) Quantitative reverse transcription‐PCR analysis of BACE1 (left) and OCLN (right) mRNA in EV‐ and BACE1‐OE‐transfected HBMECs. Transcript levels were normalized to GAPDH and are expressed as fold change relative to EV (*n* = 3 per group). For (C) and (D), bars represent mean ± SEM; individual symbols indicate independent technical replicates, and statistical analysis was performed using an unpaired two‐tailed Student's *t*‐test.
**Figure S3:** Quality control and sample‐level reproducibility of the RNA‐sequencing dataset from scramble‐control and SNCA‐silenced HBMECs. (A) Principal component analysis (PCA) of RNA‐sequencing samples from human brain microvascular endothelial cells transfected with scramble‐control siRNA (sc) or SNCA siRNA (siSNCA; *n* = 3 independent samples per condition). PCA was performed using log₂‐transformed expression values [log₂(expression +1)] of the 500 most variable genes across all six samples, selected without reference to treatment group. PC1 and PC2 explained 70.1% and 8.6% of the total variance, respectively, and clearly separated sc from siSNCA samples along PC1. (B) Sample‐to‐sample Pearson correlation heatmap generated from the same 500 most variable genes used for PCA. Values within each cell indicate the Pearson correlation coefficient between pairs of samples. Replicates within each condition showed higher correlations with one another than with samples from the other condition, supporting the reproducibility of the transcriptomic profiles and the distinction between sc and siSNCA groups.
**Figure S4:** Concordance between transcriptomic and proteomic responses to SNCA silencing in HBMECs. (A) Gene‐level comparison of RNA sequencing and proteomic changes after SNCA silencing. Each point represents a gene quantified in both datasets (*n* = 8241). The *x*‐axis shows the RNA sequencing log2 fold change for siSNCA versus scramble control (*n* = 3 per group), and the *y*‐axis shows the corresponding proteomic log2 fold change from one pooled sample per group (*n* = 1). The dashed line is the identity line. Gray points show all genes quantified in both datasets. Colored points show genes with *p*_adj_ < 0.05 and |log2FC| > 1 in the transcriptomic dataset (red, increased; blue, decreased). Selected genes are circled and labeled. Pearson correlation was *r* = 0.68 across all shared genes and *r* = 0.88 among the 331 significantly changed transcripts that were also quantified in the proteome; 96% of these genes changed in the same direction in both datasets. Because the proteomic dataset contains one pooled sample per group, this panel assesses concordance in direction and magnitude rather than statistical agreement. (B) Median log2 fold change for each of the eight predefined mechanism modules (M1‐M8, defined as in Figure 4K) in the RNA sequencing and proteomic datasets. Dark bars represent RNA sequencing (*n* = 3 per group), and light bars represent proteomics (*n* = 1 per group). The numbers at the right indicate the number of genes and proteins contributing to each module estimate. Bars extending beyond the axis range are truncated and labeled with their values. The proteomic estimate for M3 is based on one detected protein and is shown as a hatched bar; BACE1‐AS is a long non‐coding RNA and therefore is not represented in the proteomic dataset.
**Figure S5:** Endogenous SNCA is essentially restricted to the endothelium among the barrier‐forming and barrier‐supporting cells of the neurovascular unit. Western blot analysis of SNCA in human brain microvascular endothelial cells (HBMEC), normal human astrocytes (NHA) and human brain vascular pericytes. Equal amounts of protein were loaded in each lane, all three cell types were resolved on the same gel, and α‐Tubulin was used as the loading control (*n* = 3 independent lysates per cell type). Representative immunoblots are shown. SNCA was readily detected in HBMECs but was not detectable in NHA or in pericytes.
**Figure S6:** Young SNCA knockout mice already show blood–brain barrier leakage, loss of tight‐junction proteins in brain microvessels and induction of endothelial p53. (A) Representative immunofluorescence images of DAPI (cyan), CD31 (red), endogenous mouse IgG (blue) and the merged signal in the cortex, hippocampus and striatum of 9‐week‐old wild‐type (WT, 9w) and SNCA knockout (SNCA KO, 9w) mice, with quantification of mean IgG fluorescence intensity within the 10‐μm perivascular annulus after exclusion of the CD31‐positive vessel mask, expressed relative to the wild‐type mean. (B) Representative immunofluorescence images of the tight‐junction proteins ZO‐1, occludin and claudin‐5 (blue) co‐stained with CD31 (red) and DAPI (cyan) in the cortex of the same animals, with quantification of the mean gray value expressed as fold change relative to wild type. (C, D) Western blot analysis of ZO‐1, occludin, claudin‐5 and SNCA in whole‐brain lysates (C) and in isolated brain microvessel fractions (D; the enrichment of this fraction is shown in Figure S10), with the corresponding densitometric quantification. (E, F) Western blot analysis of p53 and SNCA in whole‐brain lysates (E) and in isolated brain microvessel fractions (F), with the corresponding densitometric quantification. (G) Representative immunofluorescence images of DAPI (cyan), CD31 (red), p53 (blue) and the merged signal in the cortex, hippocampus, striatum, white matter and cerebellum of the same animals, with quantification of the p53 mean gray value in each region expressed as fold change relative to wild type. α‐Tubulin was used as the loading control for all western blots, and representative immunoblots are shown. Bars represent mean ± SEM (*n* = 6 mice per genotype); individual symbols indicate individual mice. Statistical analysis was performed using an unpaired two‐tailed Student's *t*‐test. Scale bars, 100 μm.
**Figure S7:** Effect of SNCA silencing on tight‐junction proteins in HBMECs exposed to 1 μM Aβ42 oligomer. (A) Upper panel: western blot analysis of ZO‐1, occludin and claudin‐5 in HBMECs transfected with scramble control siRNA (sc) or SNCA siRNA (siSNCA) and treated with DMSO or 1 μM Aβ42 oligomer for 24 h (*n* = 3 per condition). α‐Tubulin was used as the loading control. Representative immunoblots are shown. Lower panels: corresponding densitometric analysis for ZO‐1, claudin‐5 and occludin. Bars represent mean ± SEM; individual symbols indicate independent technical replicates. All HBMECs were used between passages 5 and 8. Statistical analysis was performed using two‐way ANOVA followed by Tukey's multiple‐comparisons test. (B) Left: schematic of the in vitro blood–brain barrier permeability assay, in which HBMEC monolayers cultured on Transwell inserts were treated with DMSO or Aβ oligomer and FITC‐dextran added to the apical medium was measured in the basolateral compartment. Right: quantification of FITC‐dextran permeability in sc‐ and siSNCA‐transfected HBMEC monolayers treated with DMSO or 1 μM Aβ42 oligomer. Bars represent mean ± SEM; individual symbols indicate independent technical replicates. Statistical analysis was performed using two‐way ANOVA followed by Tukey's multiple‐comparisons test.
**Figure S8:** Effect of SNCA silencing on tight‐junction proteins in HBMECs exposed to 5 μM Aβ42 oligomer. (A) Upper panel: western blot analysis of ZO‐1, occludin and claudin‐5 in HBMECs transfected with scramble control siRNA (sc) or SNCA siRNA (siSNCA) and treated with DMSO or 5 μM Aβ42 oligomer for 24 h (*n* = 3 per condition). α‐Tubulin was used as the loading control. Representative immunoblots are shown. Lower panels: corresponding densitometric analysis for ZO‐1, claudin‐5 and occludin. Bars represent mean ± SEM; individual symbols indicate independent technical replicates. All HBMECs were used between passages 5 and 8. Statistical analysis was performed using two‐way ANOVA followed by Tukey's multiple‐comparisons test. (B) Left: schematic of the in vitro blood–brain barrier permeability assay, in which HBMEC monolayers cultured on Transwell inserts were treated with DMSO or Aβ oligomer and FITC‐dextran added to the apical medium was measured in the basolateral compartment. Right: quantification of FITC‐dextran permeability in sc‐ and siSNCA‐transfected HBMEC monolayers treated with DMSO or 5 μM Aβ42 oligomer. Bars represent mean ± SEM; individual symbols indicate independent technical replicates. Statistical analysis was performed using two‐way ANOVA followed by Tukey's multiple‐comparisons test.
**Figure S9:** CD31 immunoblotting confirms enrichment of brain microvessels in aged and young wild‐type mice. (A) Western blot of CD31 in the whole‐brain lysate and in the parenchymal and microvessel fractions prepared from young and aged wild‐type mice. (B) α‐Tubulin in the same lysates, used as the loading control. Within each panel all three fractions of both groups were run together and imaged in a single exposure, so that the signals can be compared directly. CD31 was enriched in the microvessel fraction relative to the whole‐brain lysate and was lowest in the parenchymal fraction, confirming that the fractionation separates brain microvessels from the surrounding parenchyma. Equal amounts of protein were loaded in each lane. Molecular weight markers (kDa) are indicated to the right of each blot. *n* = 7 mice per group. These are the fractions analyzed in Figure 6J–M.
**Figure S10:** CD31 immunoblotting confirms enrichment of brain microvessels in 9‐week‐old wild‐type and SNCA knockout mice. (A) Western blot of CD31 in the whole‐brain lysate and in the parenchymal and microvessel fractions prepared from 9‐week‐old wild‐type and SNCA knockout mice. (B) α‐Tubulin in the same lysates, used as the loading control. Within each panel all three fractions of both groups were run together and imaged in a single exposure, so that the signals can be compared directly. CD31 was enriched in the microvessel fraction relative to the whole‐brain lysate and was lowest in the parenchymal fraction, confirming that the fractionation separates brain microvessels from the surrounding parenchyma. Equal amounts of protein were loaded in each lane. Molecular weight markers (kDa) are indicated to the right of each blot. *n* = 6 mice per genotype. These are the fractions analyzed in Figure S6.

## Data Availability

The datasets used and/or analyzed in the current study are available from the corresponding author upon reasonable request. The RNA‐sequencing data have been deposited in the DDBJ Sequence Read Archive under BioProject accession PRJDB45894 and Run accession numbers DRR1213168–DRR1213173. The proteomic data have been deposited in the jPOST Repository under accession JPST004988 (https://repository.jpostdb.org/entry/JPST004988).
