# Supplementary material for: Senescence‐Associated Loss of α‐Synuclein in Brain Microvascular Endothelium Compromises Tight Junctions and Disrupts Blood–Brain Barrier Integrity
Source: Aging Cell. 2026 Nov 9;25(11):e70770. doi: 10.1111/acel.70770 (PMC13746884; doi:10.1111/acel.70770)

<u>**Supplementary Figures**</u>

Supplemental Figure 1

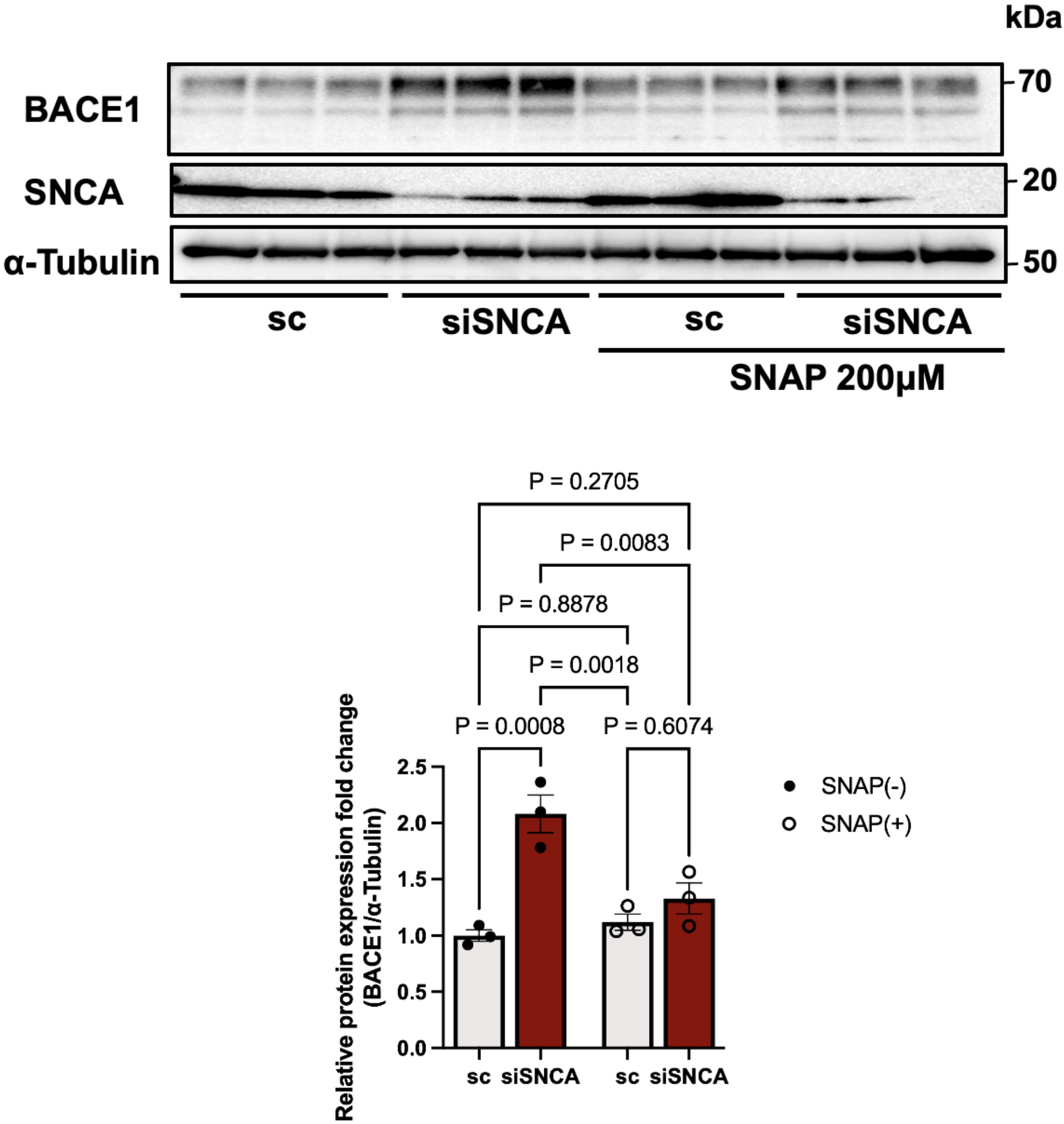

Supplemental Figure 2

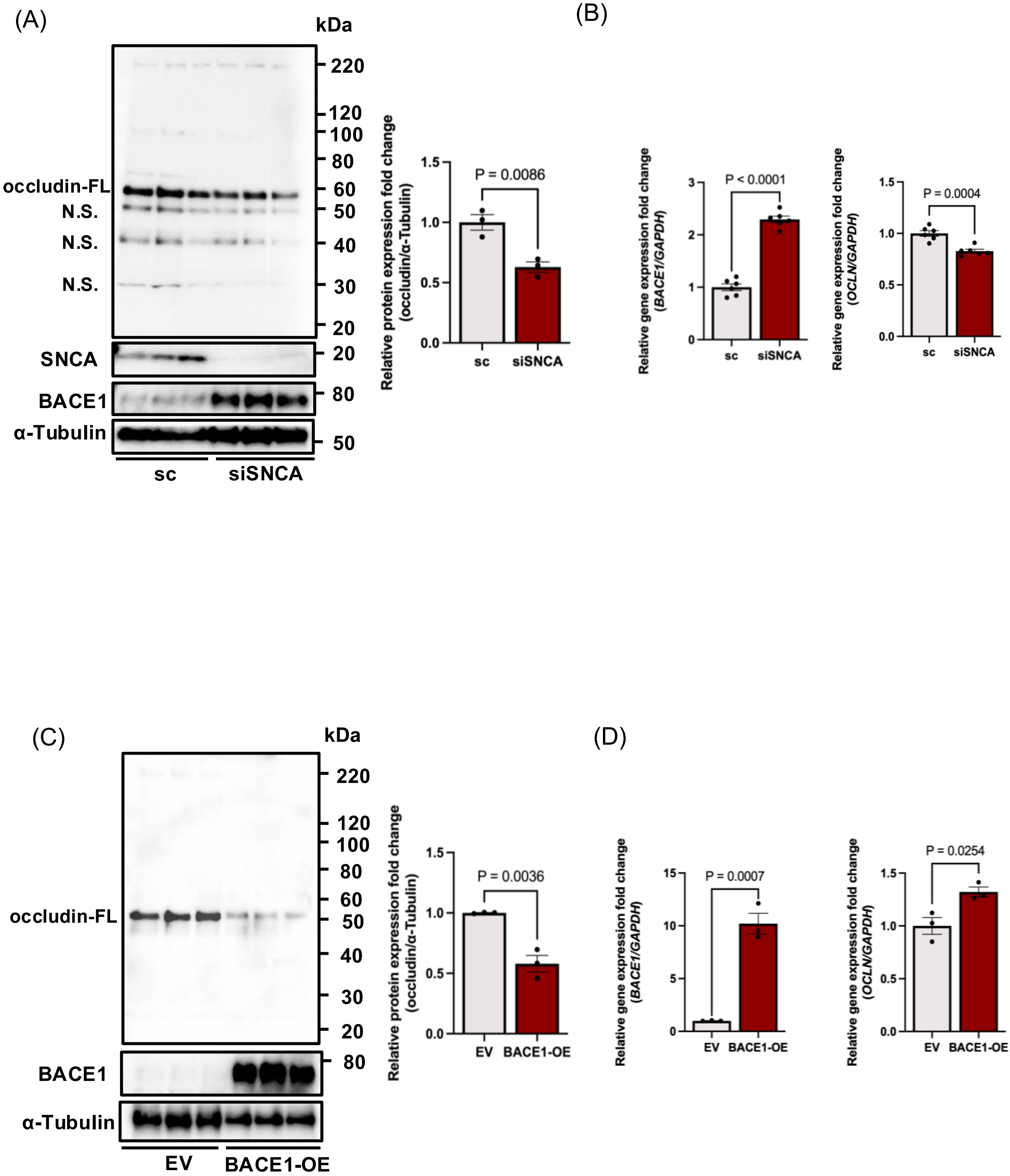

Supplemental Figure 3

(A)

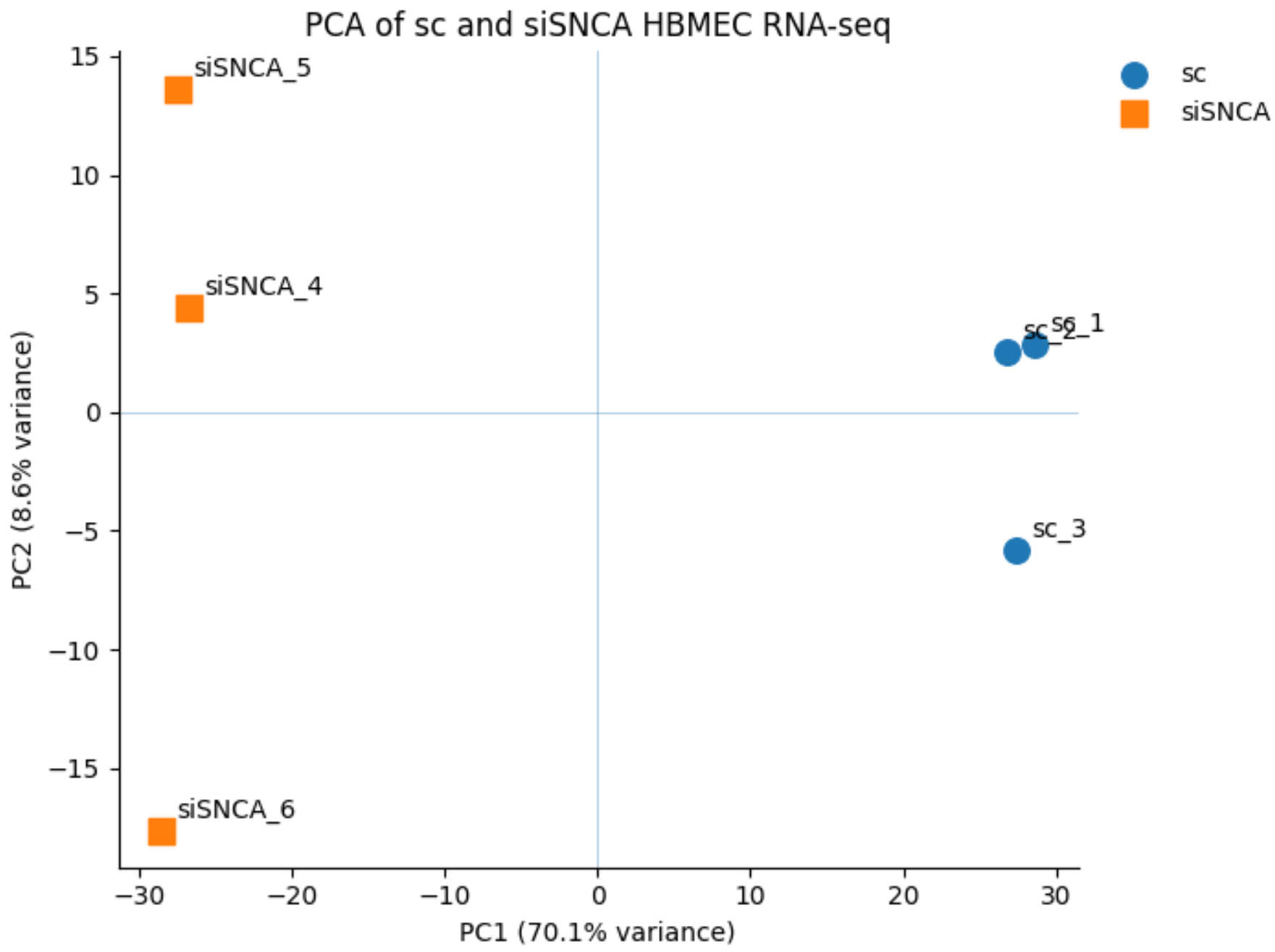

(B)

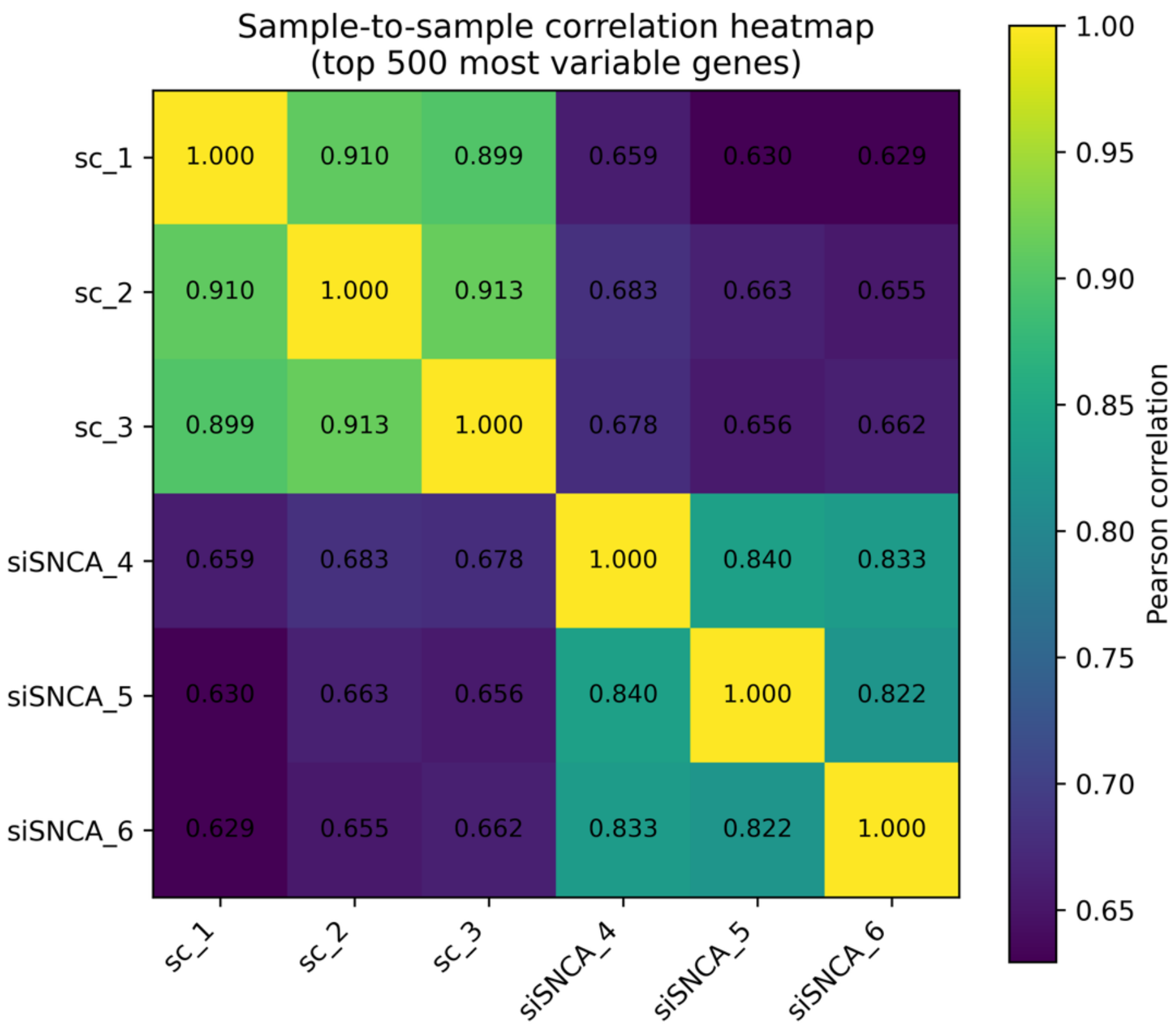

Supplemental Figure 4

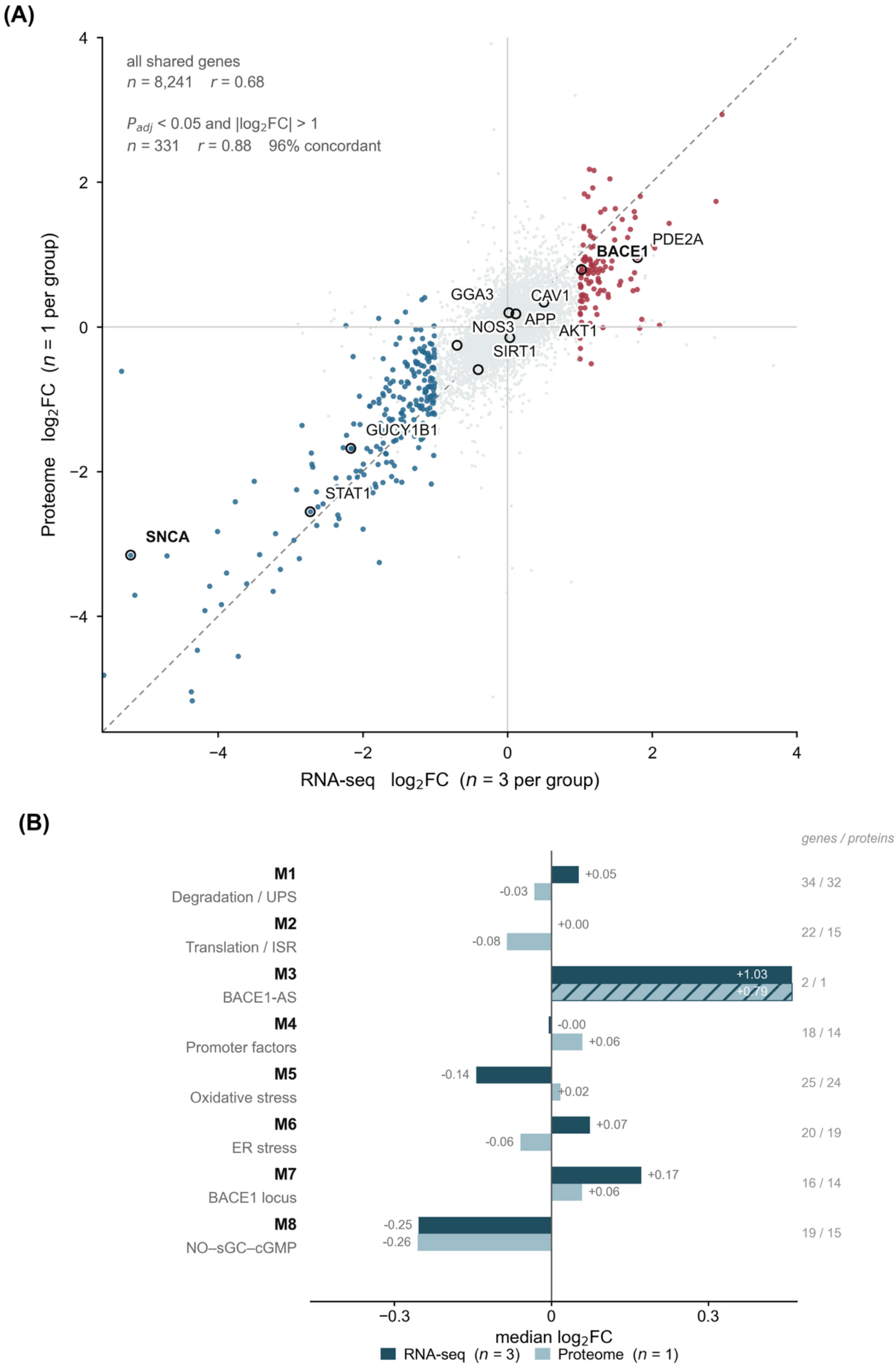

Supplemental Figure 5

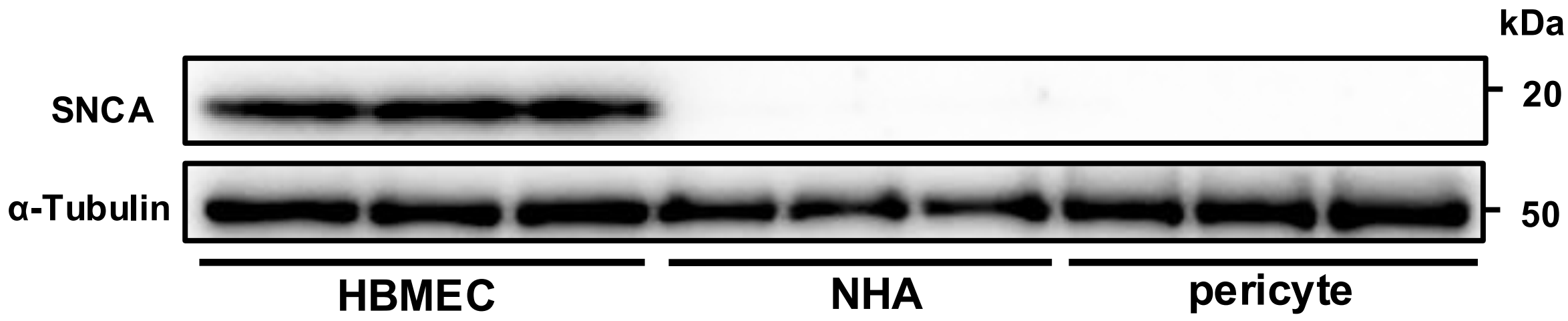

Supplemental Figure 6

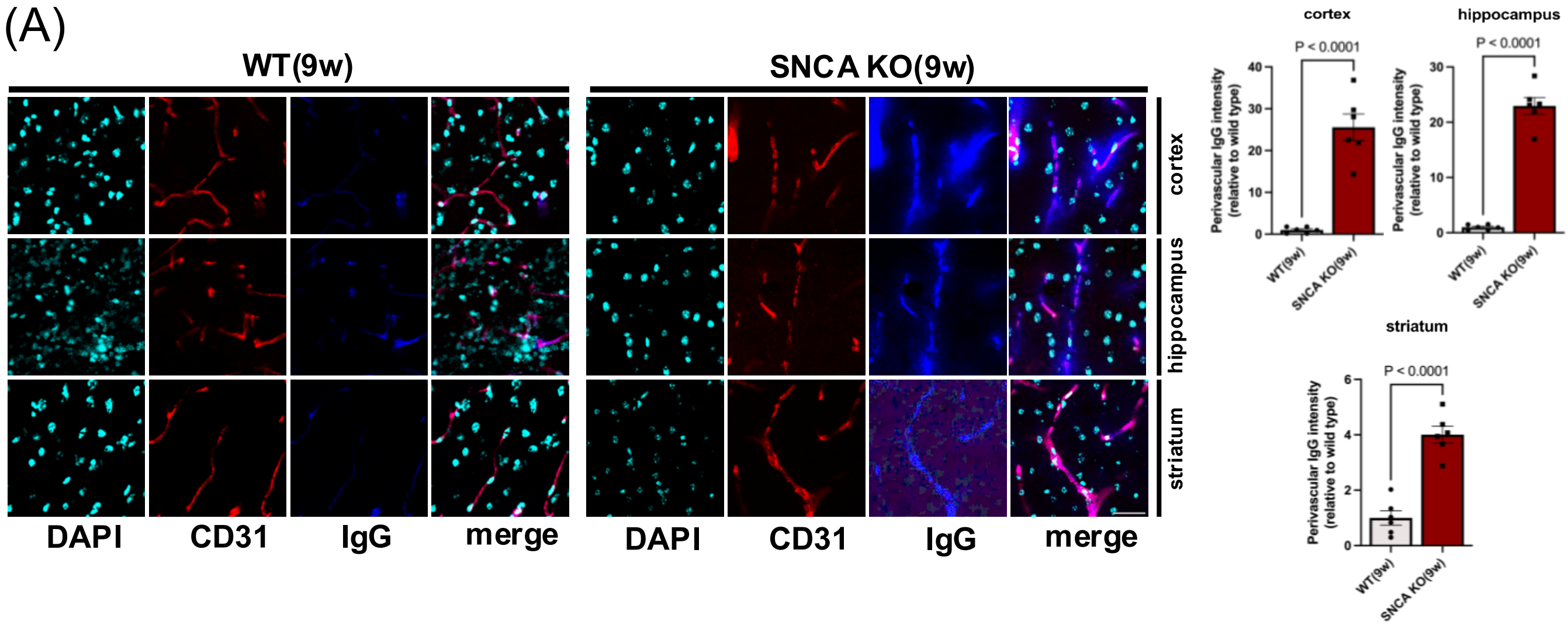

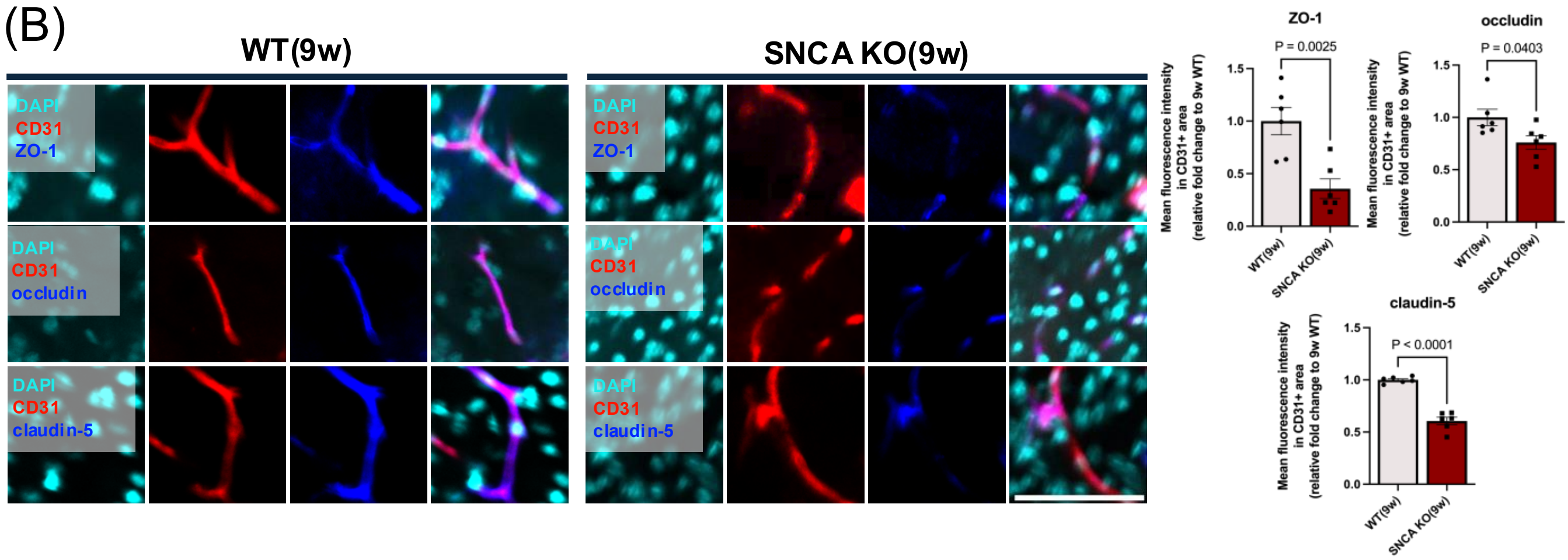

Supplemental Figure 6

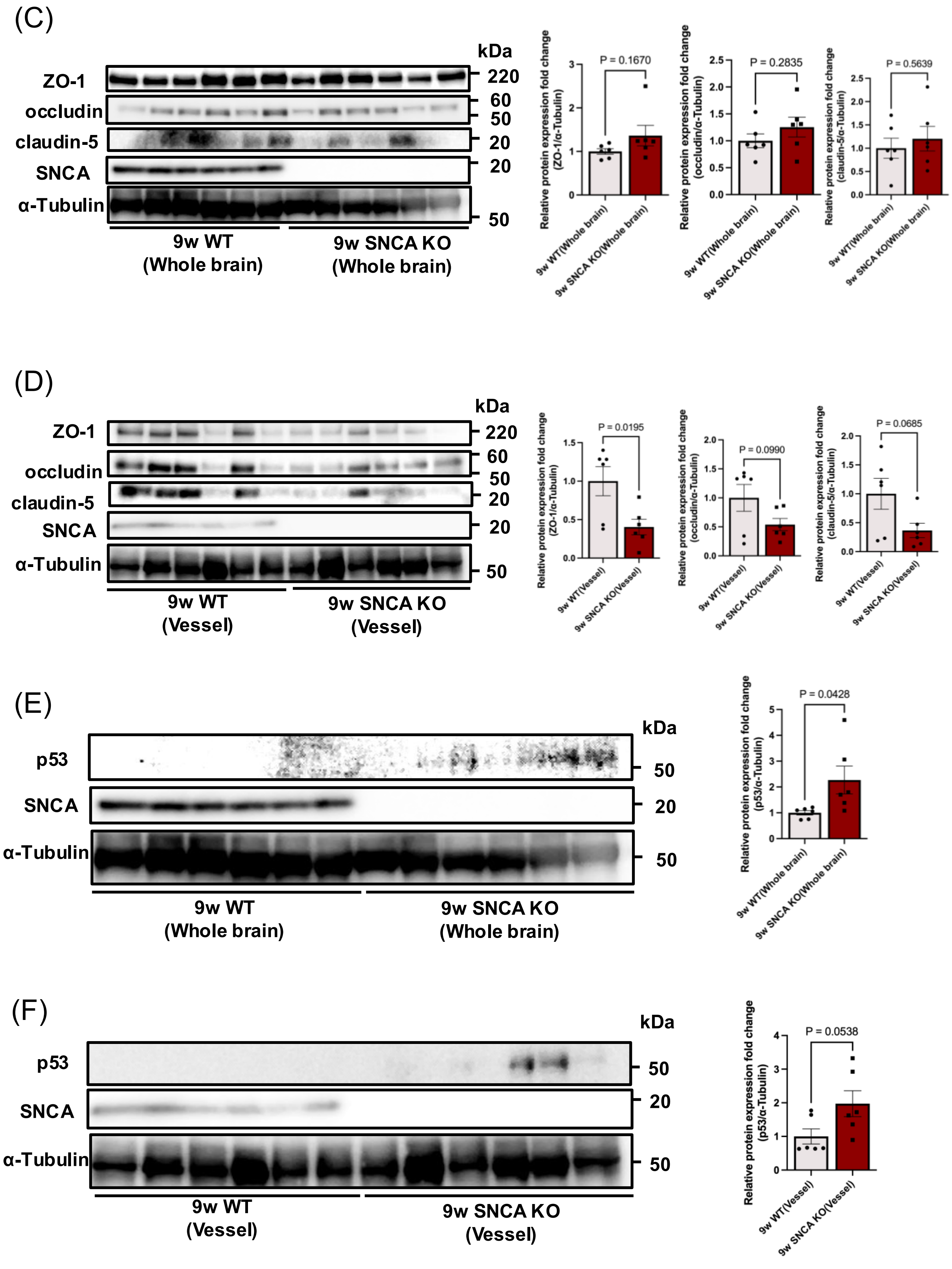

Supplemental Figure 6

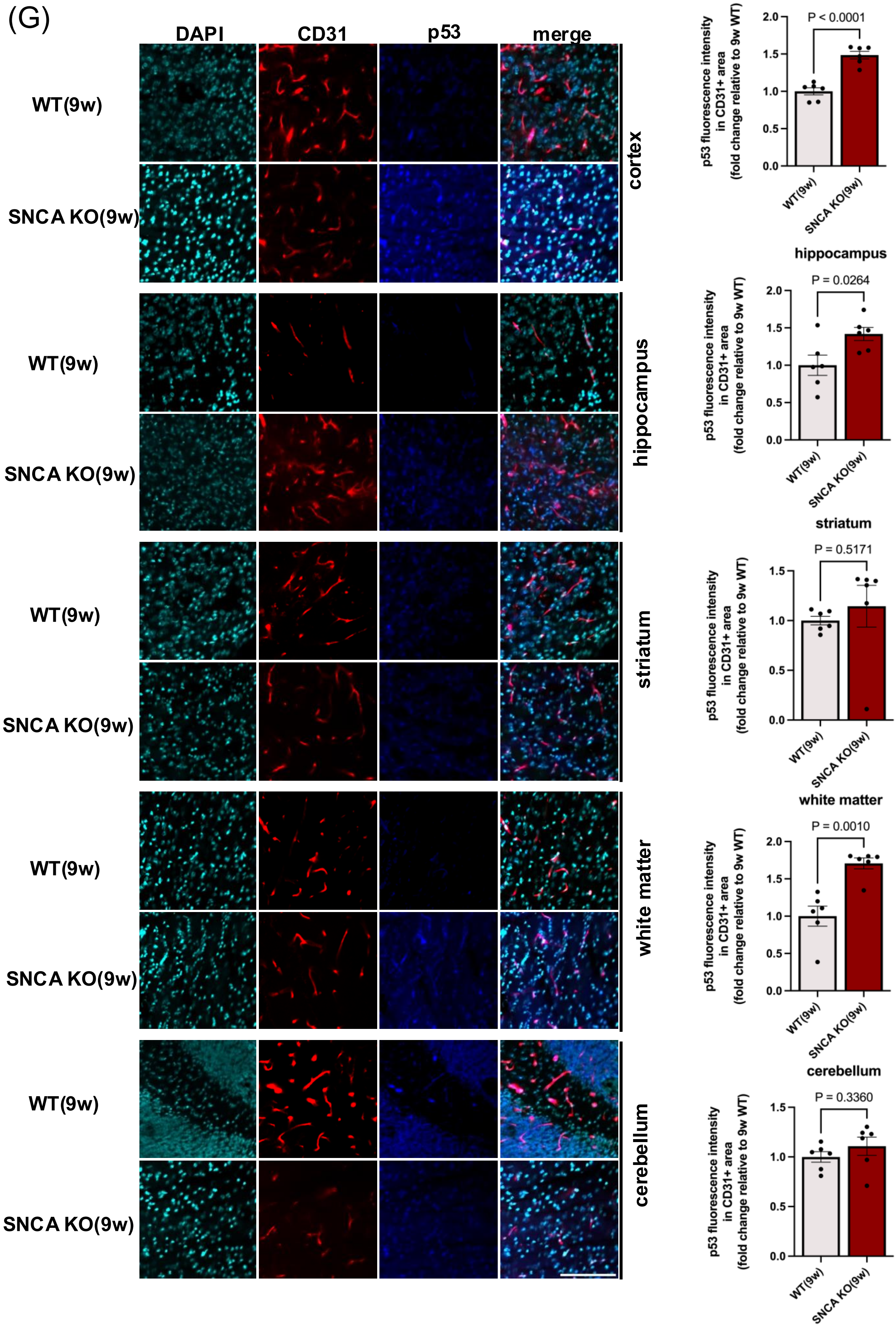

Supplemental Figure 7

(A)

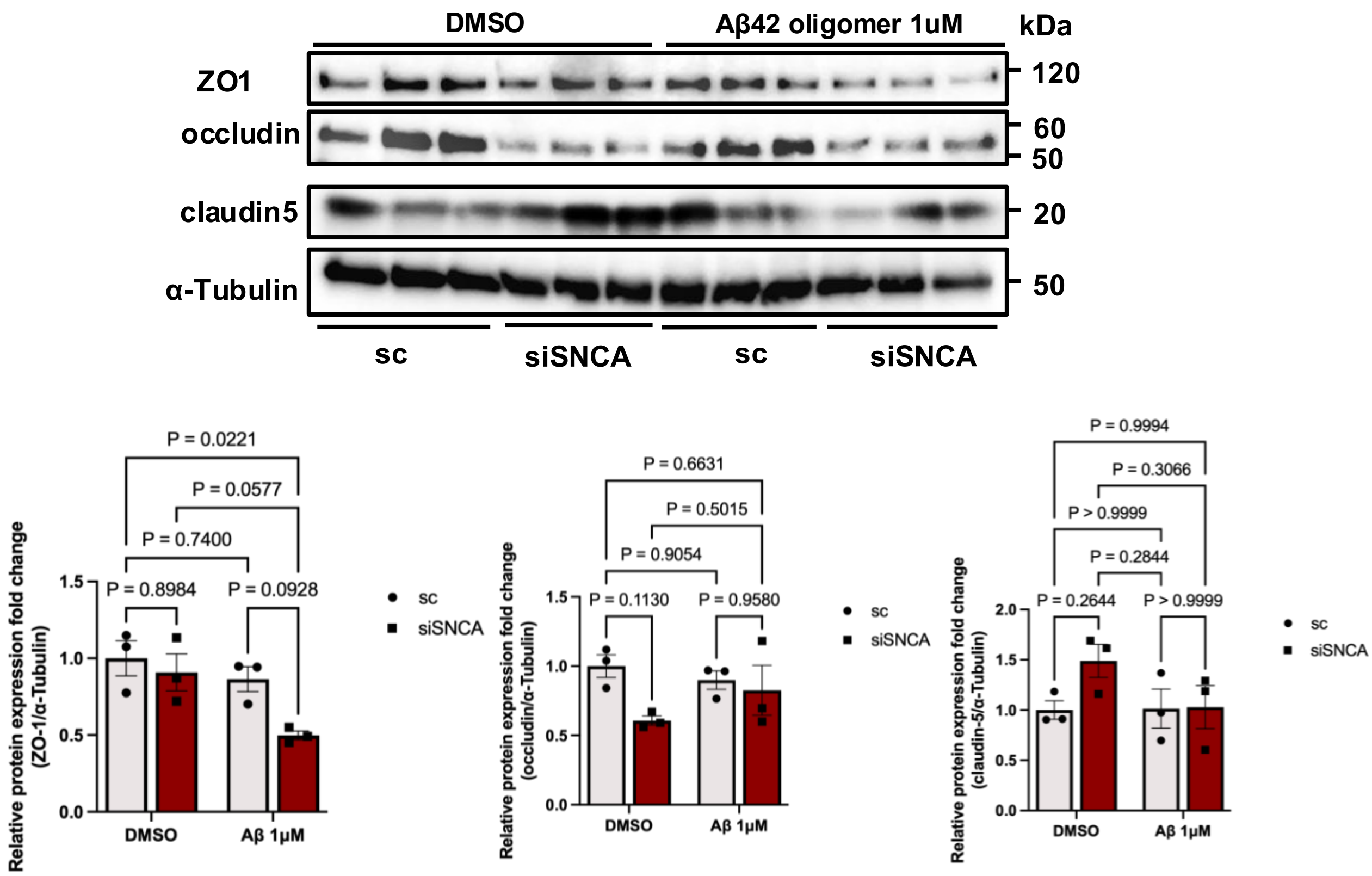

(B)

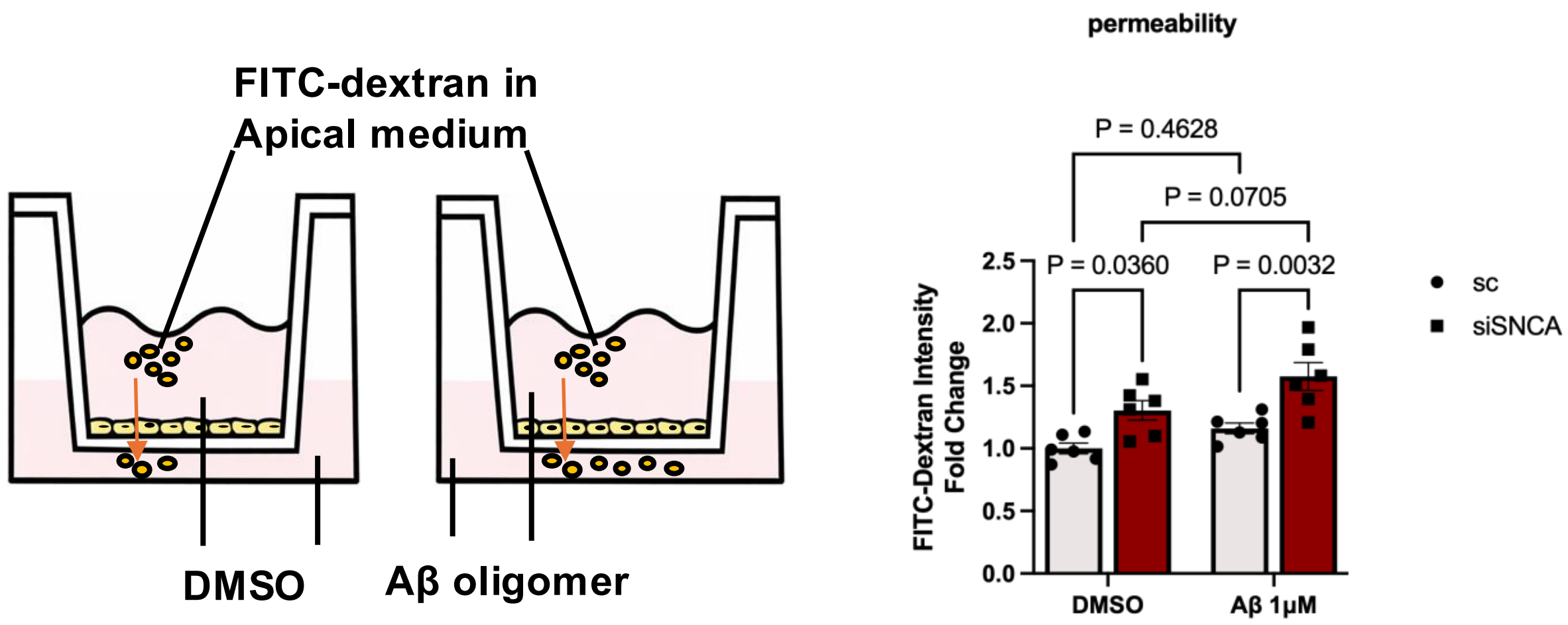

Supplemental Figure 8

(A)

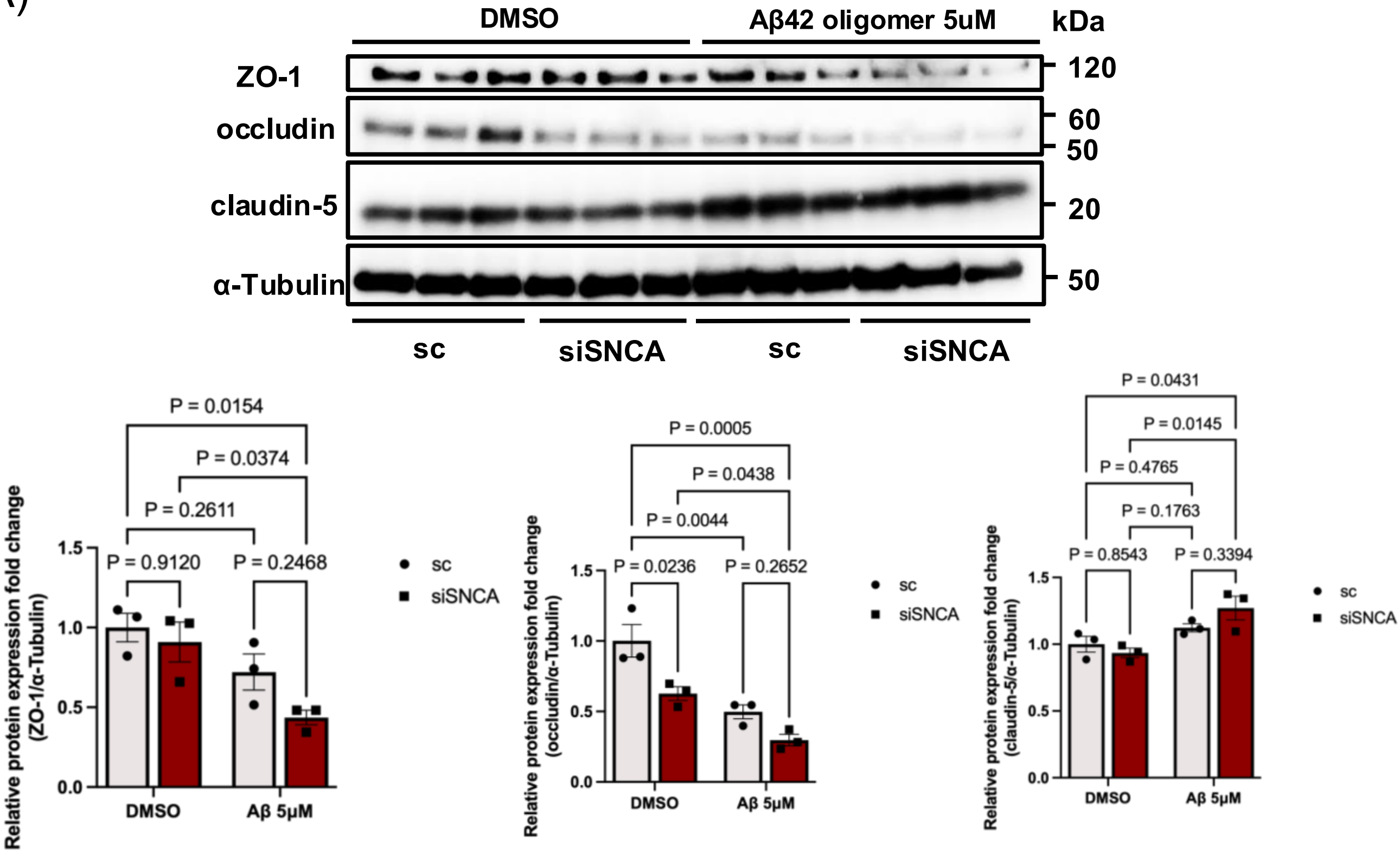

(B)

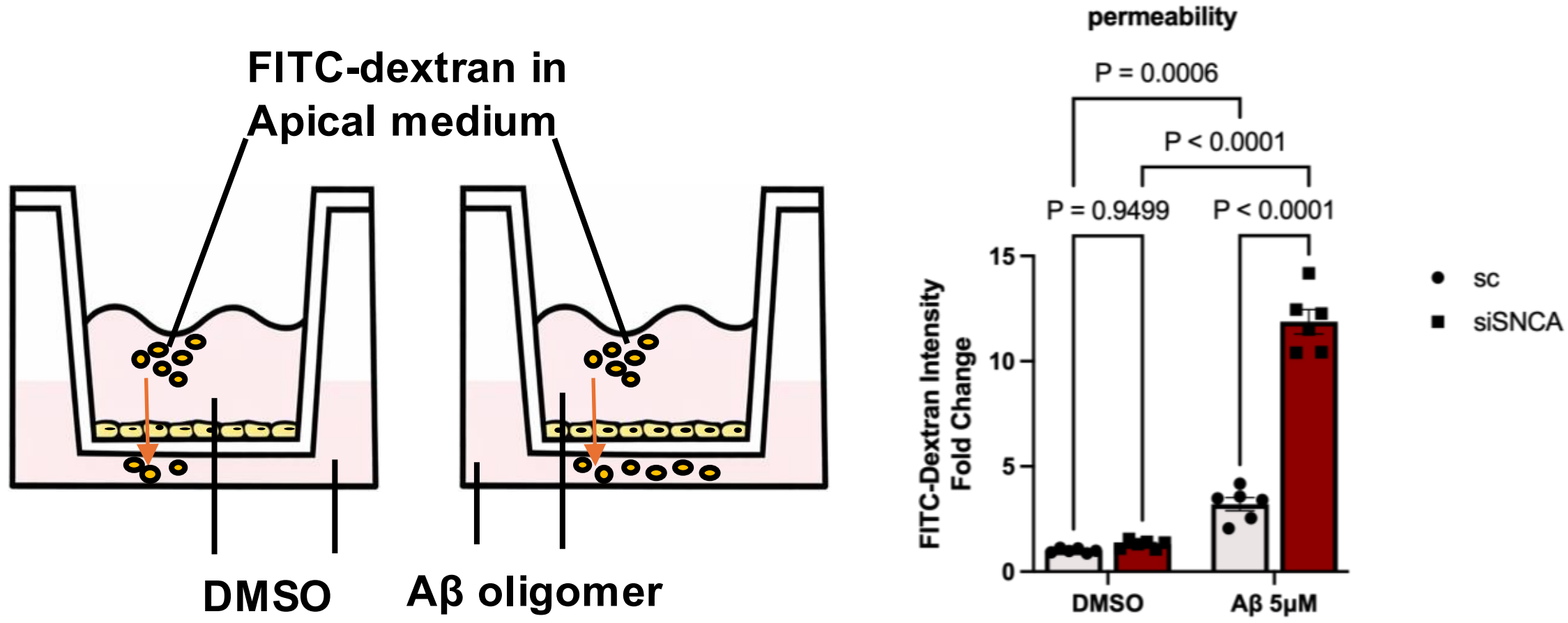

Supplemental Figure 9

(A)

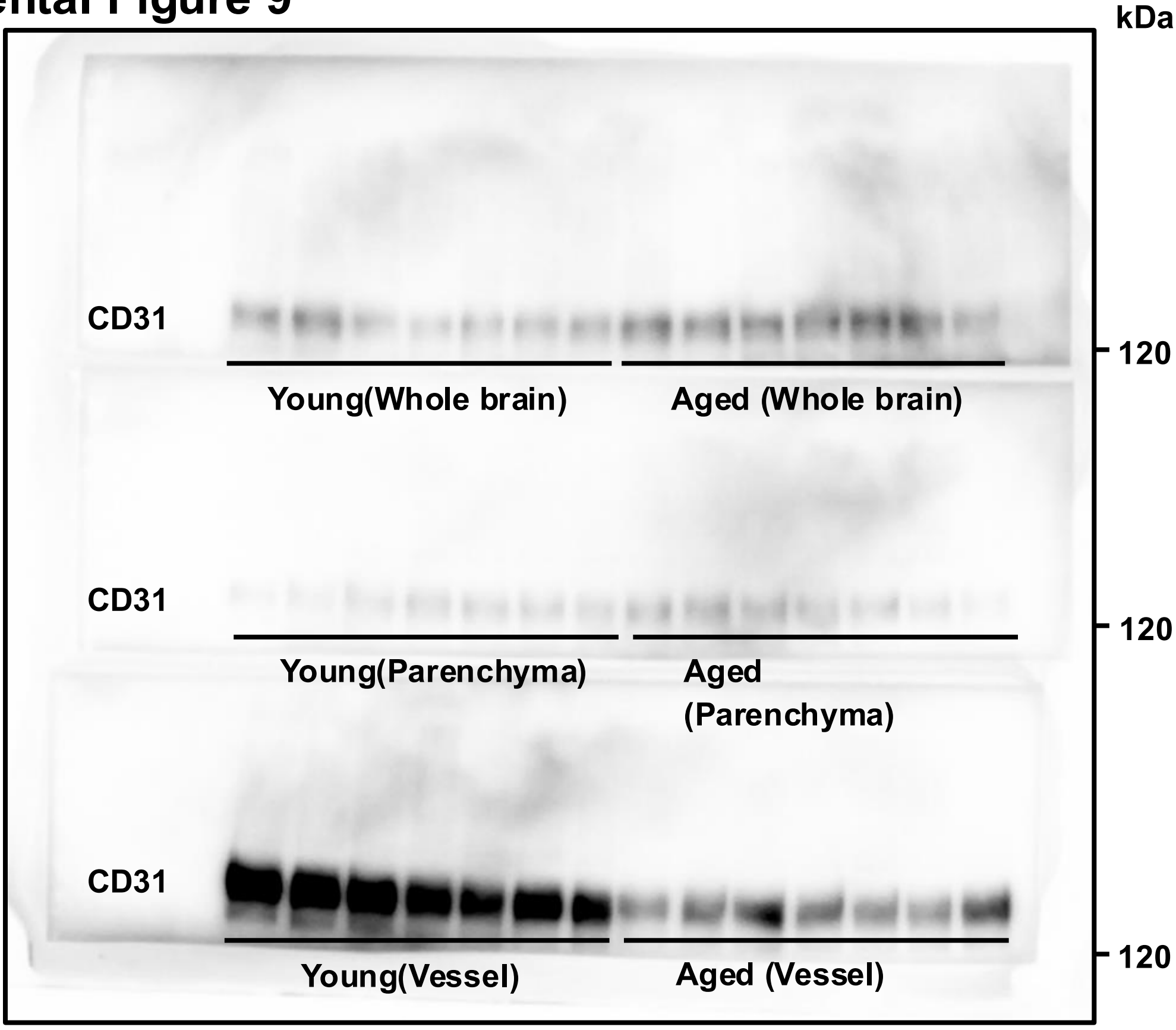

(B)

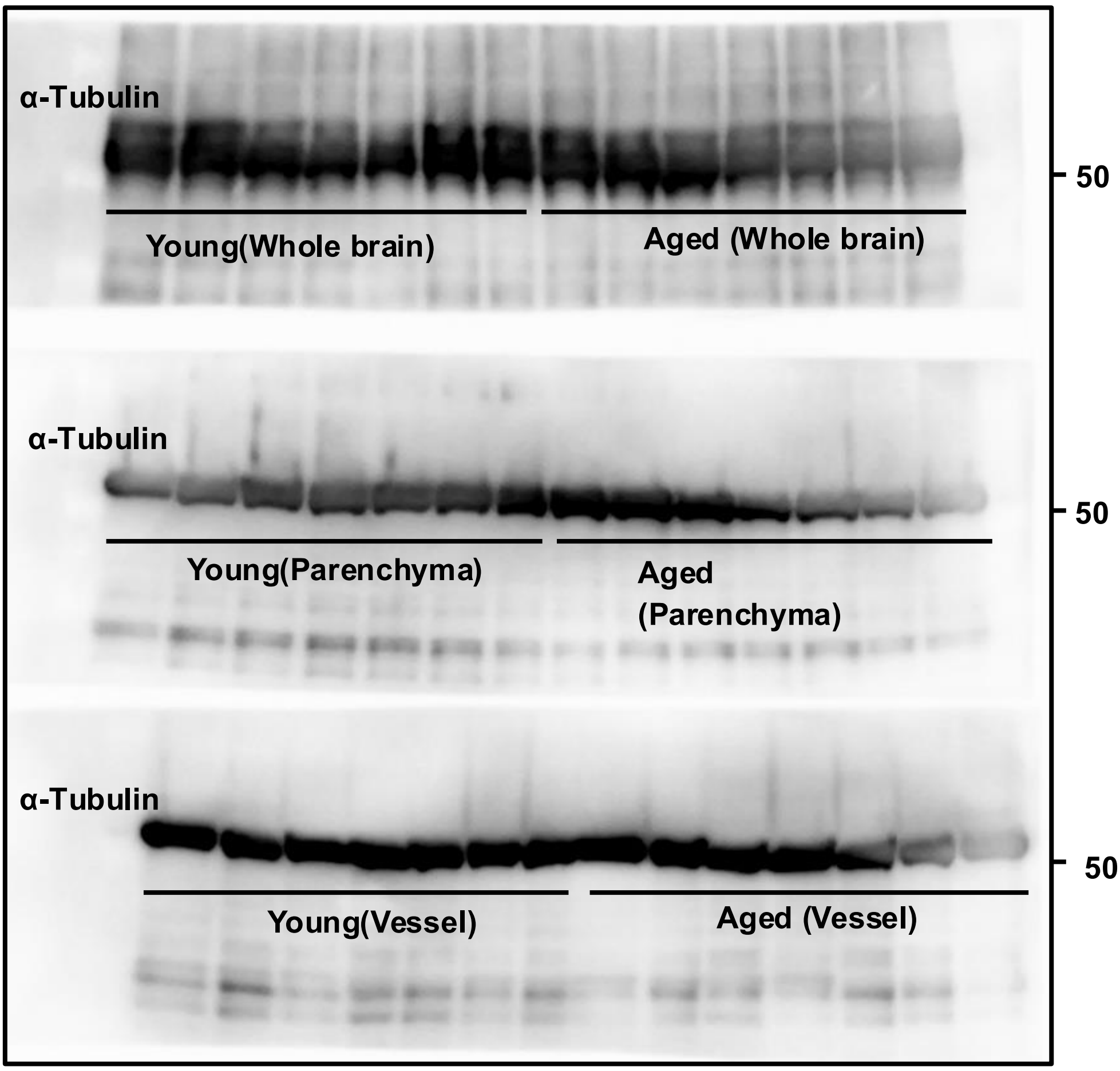

Supplemental Figure 10

(A)

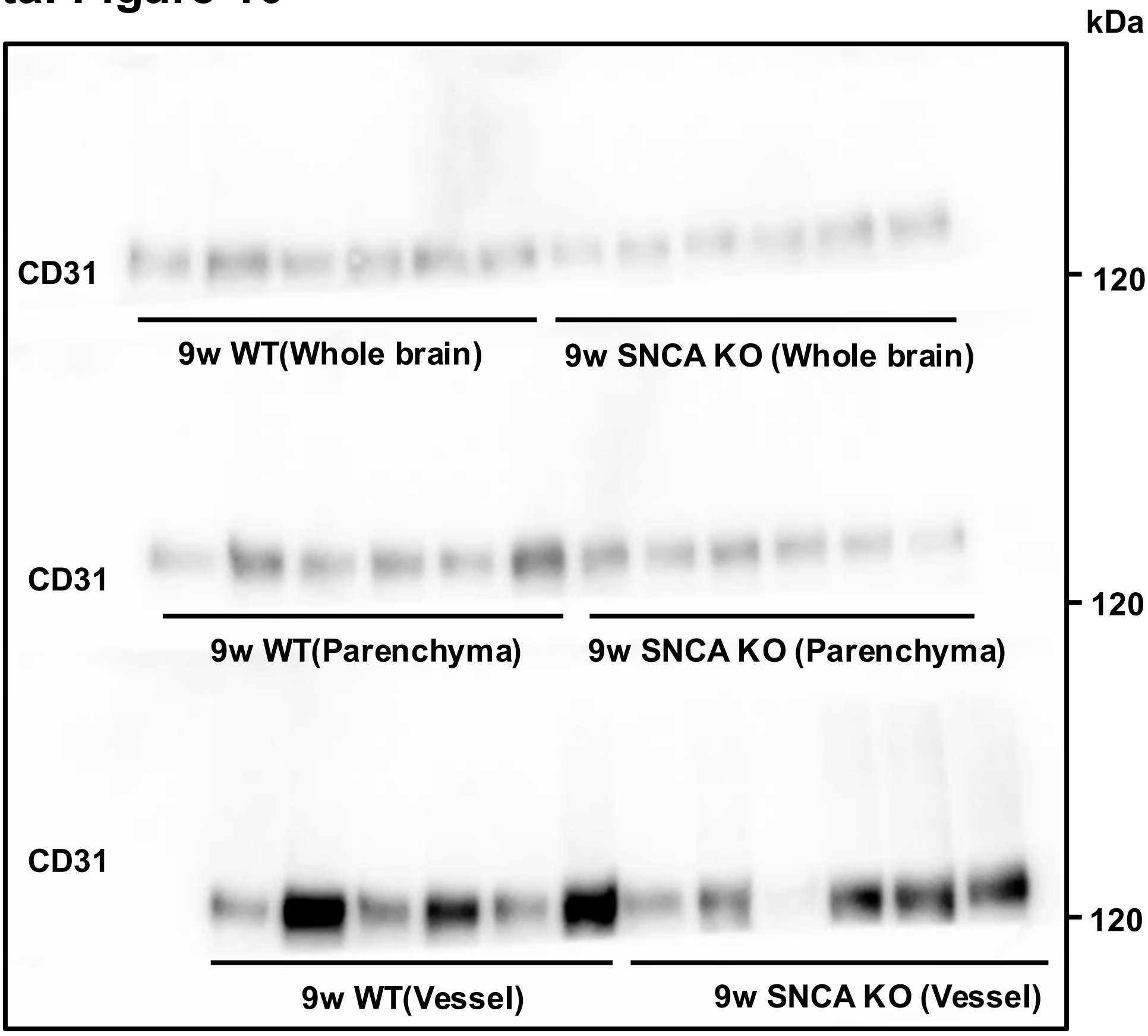

(B)

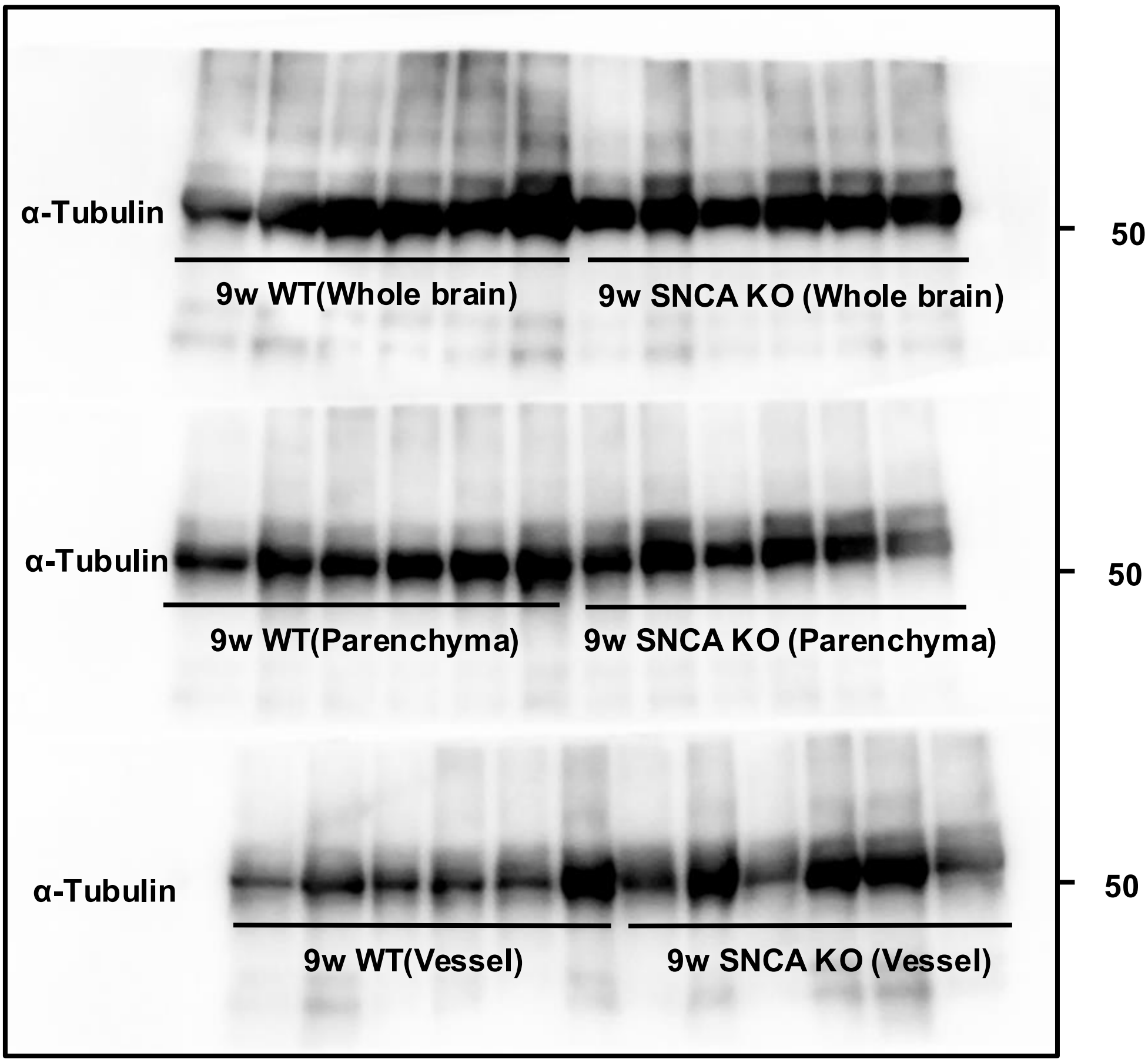

Supplement: Supplementary file 1 [file ACEL-25-e70770-s002.pdf]
