# Supplementary material for: Senescence‐Associated Loss of α‐Synuclein in Brain Microvascular Endothelium Compromises Tight Junctions and Disrupts Blood–Brain Barrier Integrity
Source: Aging Cell. 2026 Nov 9;25(11):e70770. doi: 10.1111/acel.70770 (PMC13746884; doi:10.1111/acel.70770)

Unedited full blot for Figure 1C.

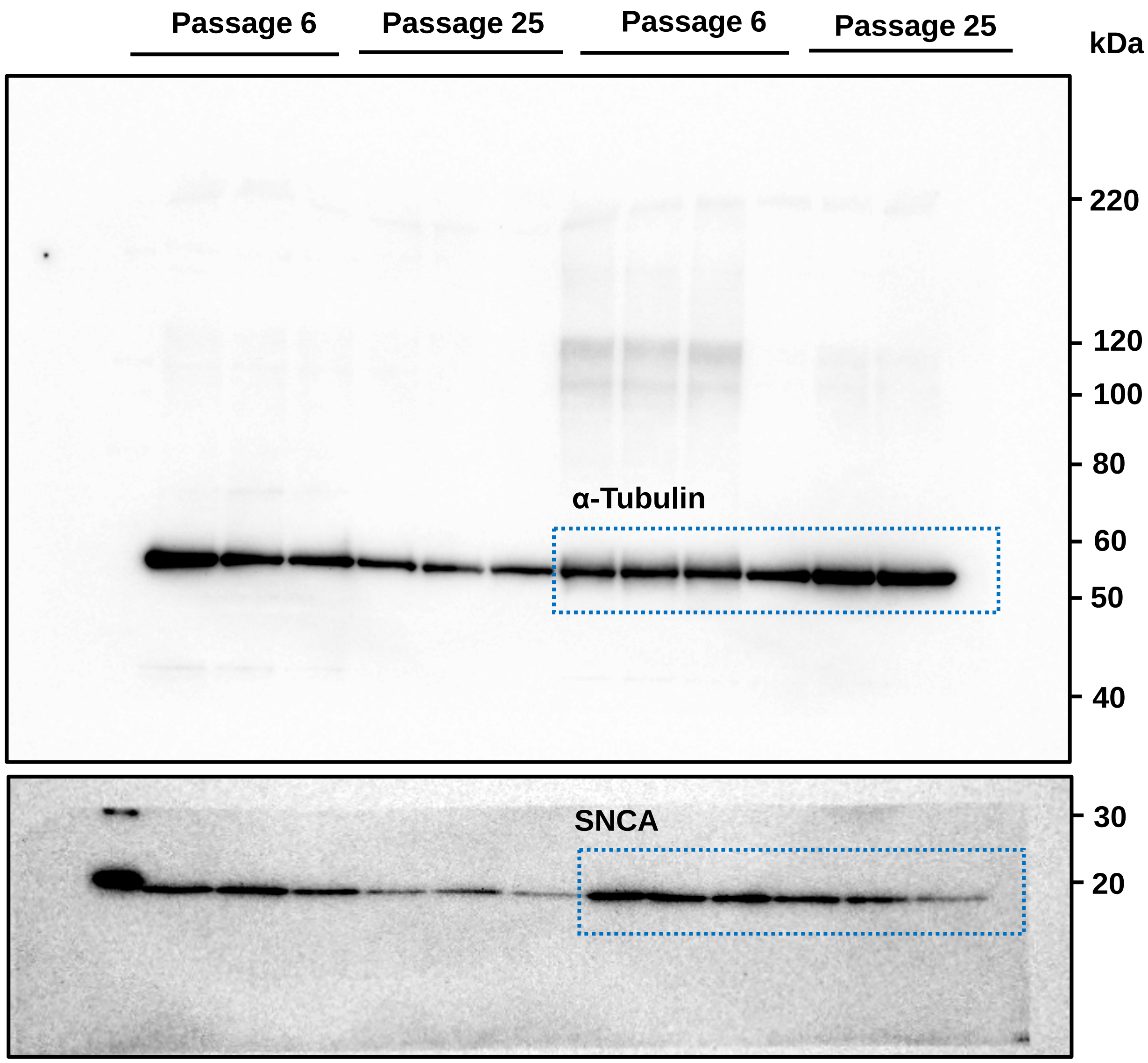

Unedited full blot for Figure 2D-1.

(for representative figure and densitometric analysis).

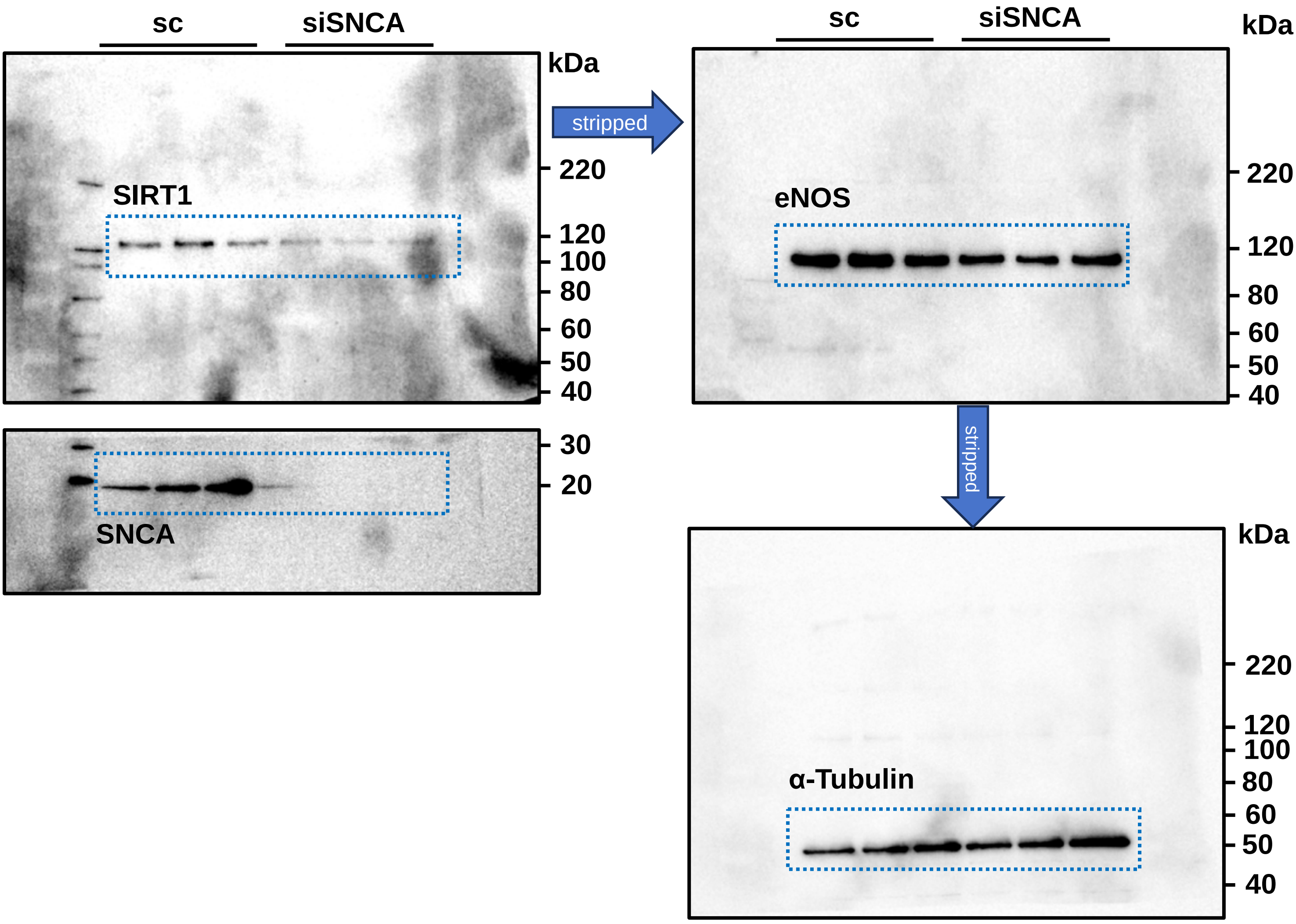

Unedited full blot for Figure 2D-2, 2D-3.

(for densitometric analysis).

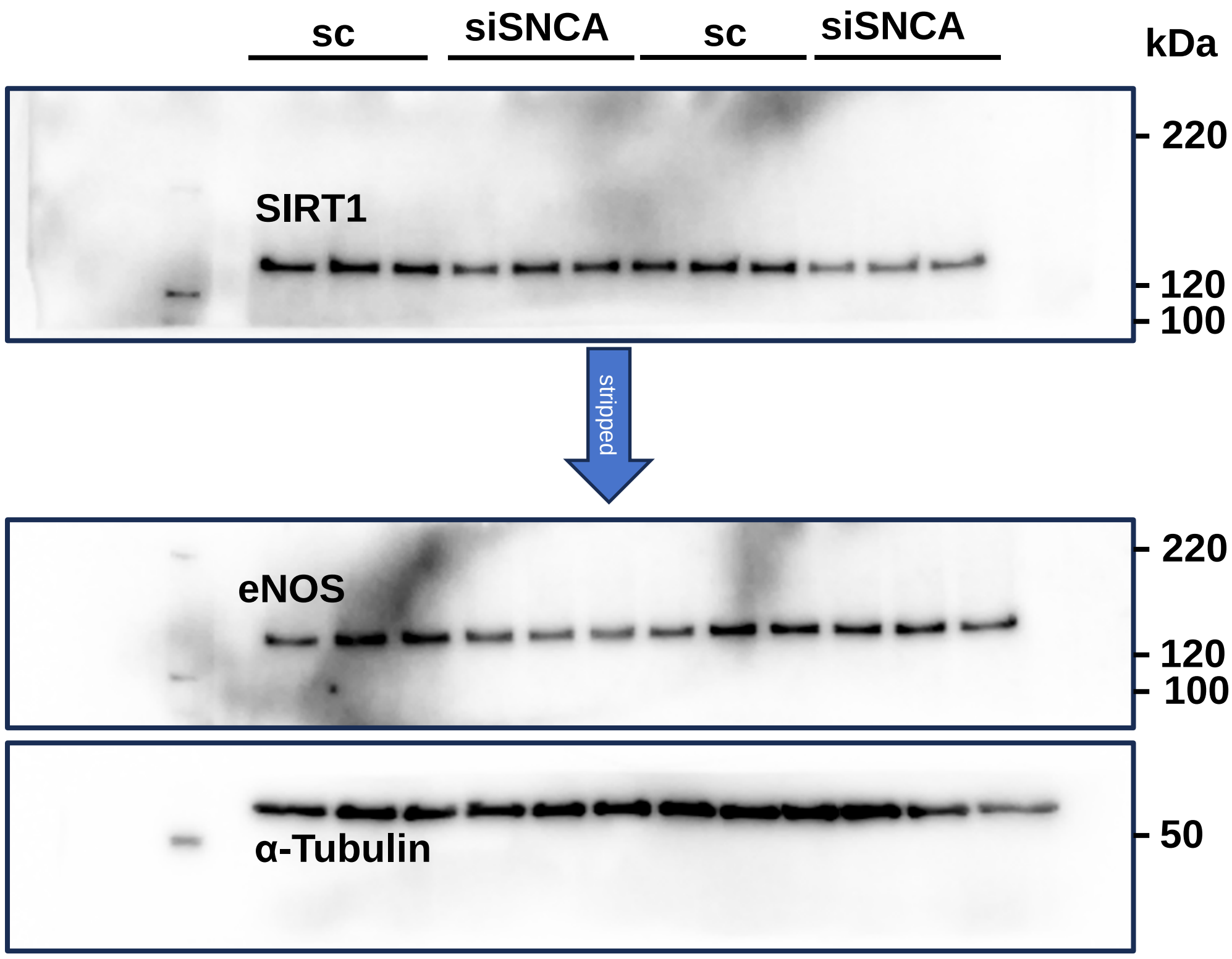

Unedited full blot for Figure 2E-1，2E-2.

(for representative figure and densitometric analysis).

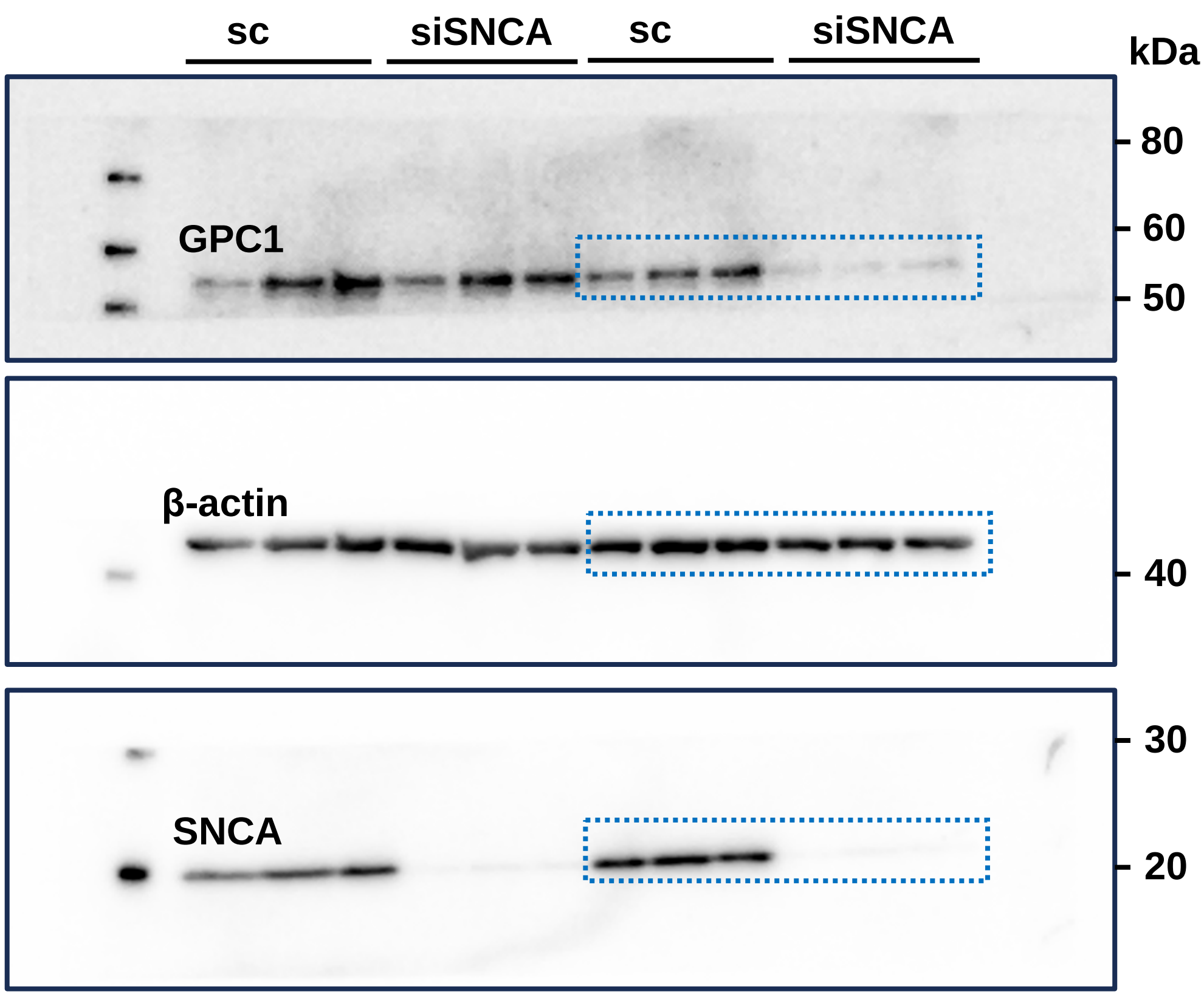

Unedited full blot for Figure 2E-3.

(for densitometric analysis).

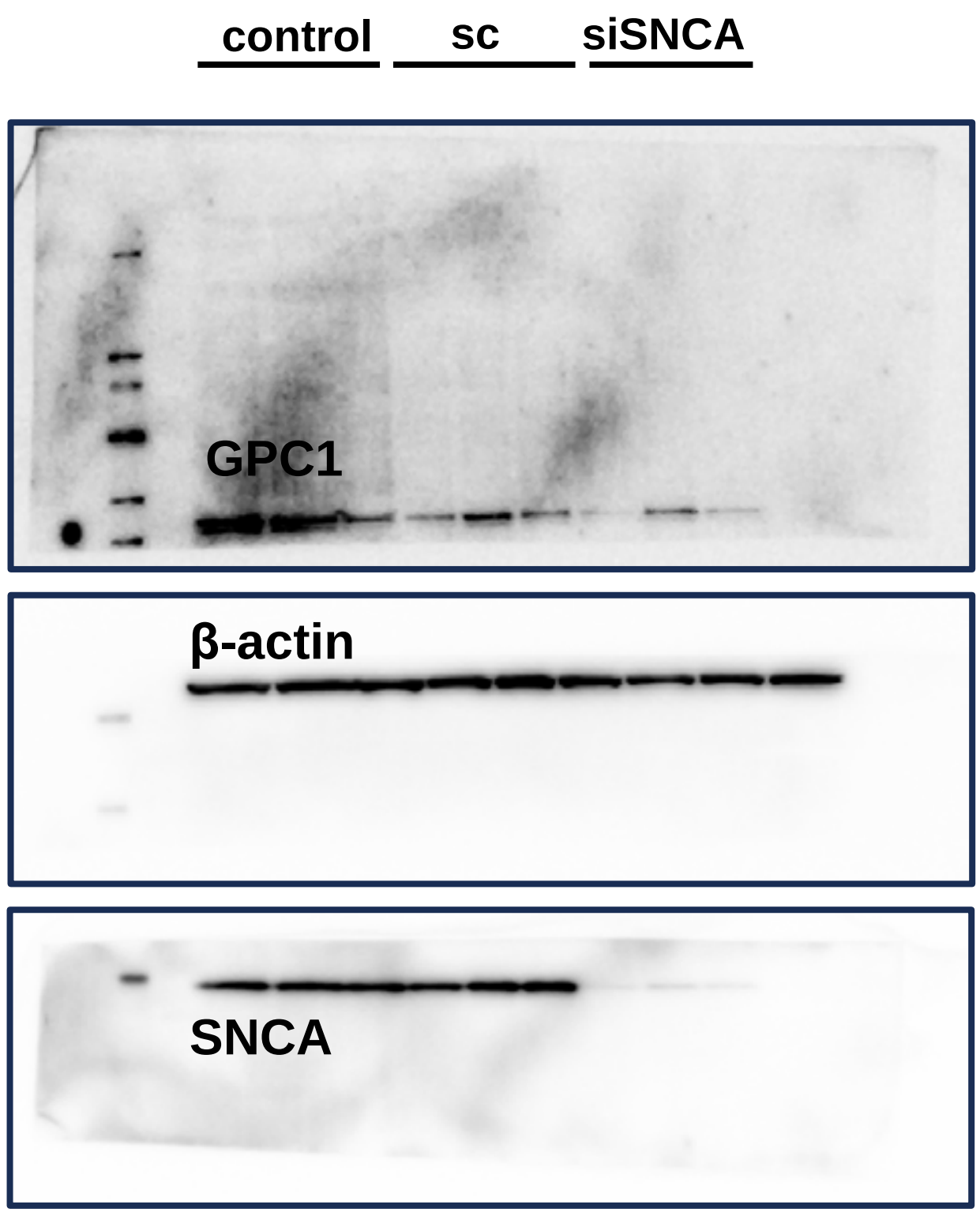

Unedited full blot for Figure 4A.

(for representative figure and densitometric analysis).

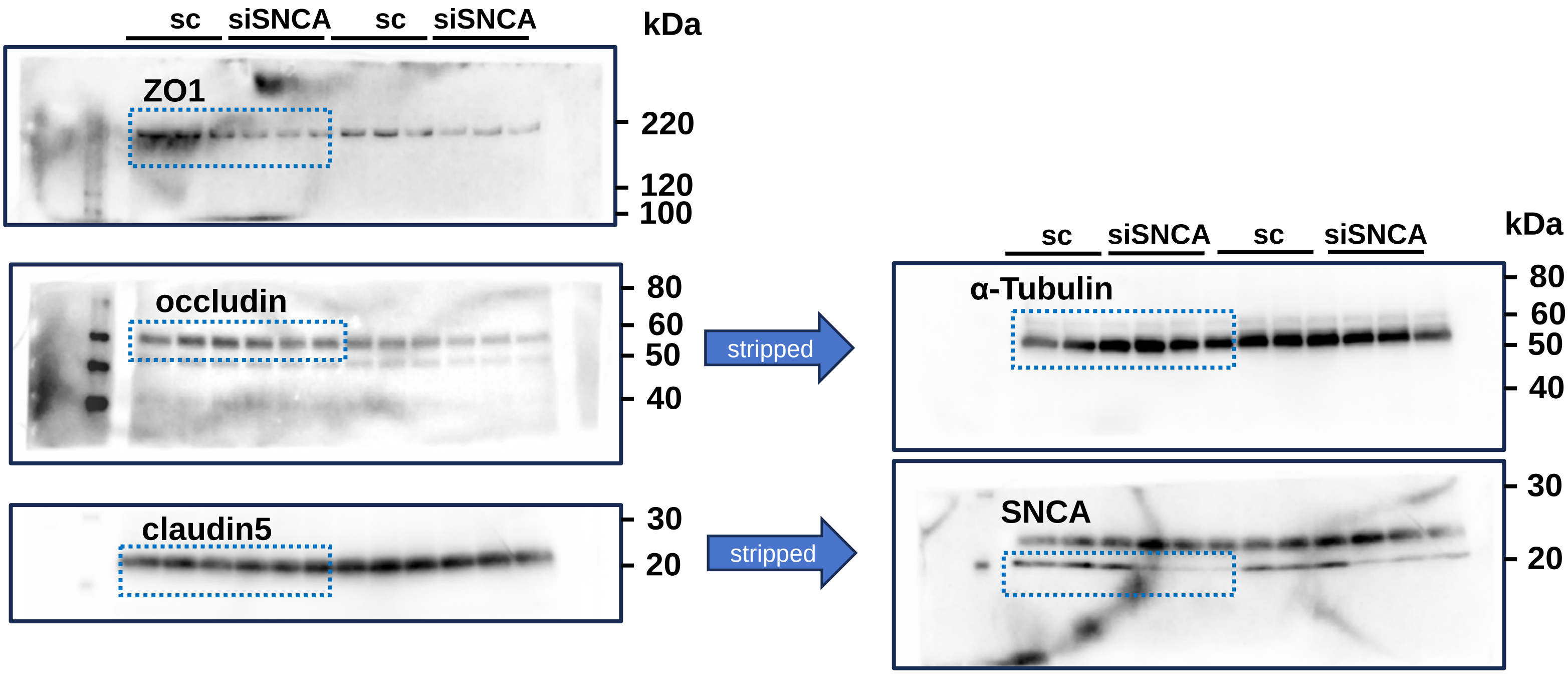

Unedited full blot for Figure 4D.

(for representative figure and densitometric analysis).

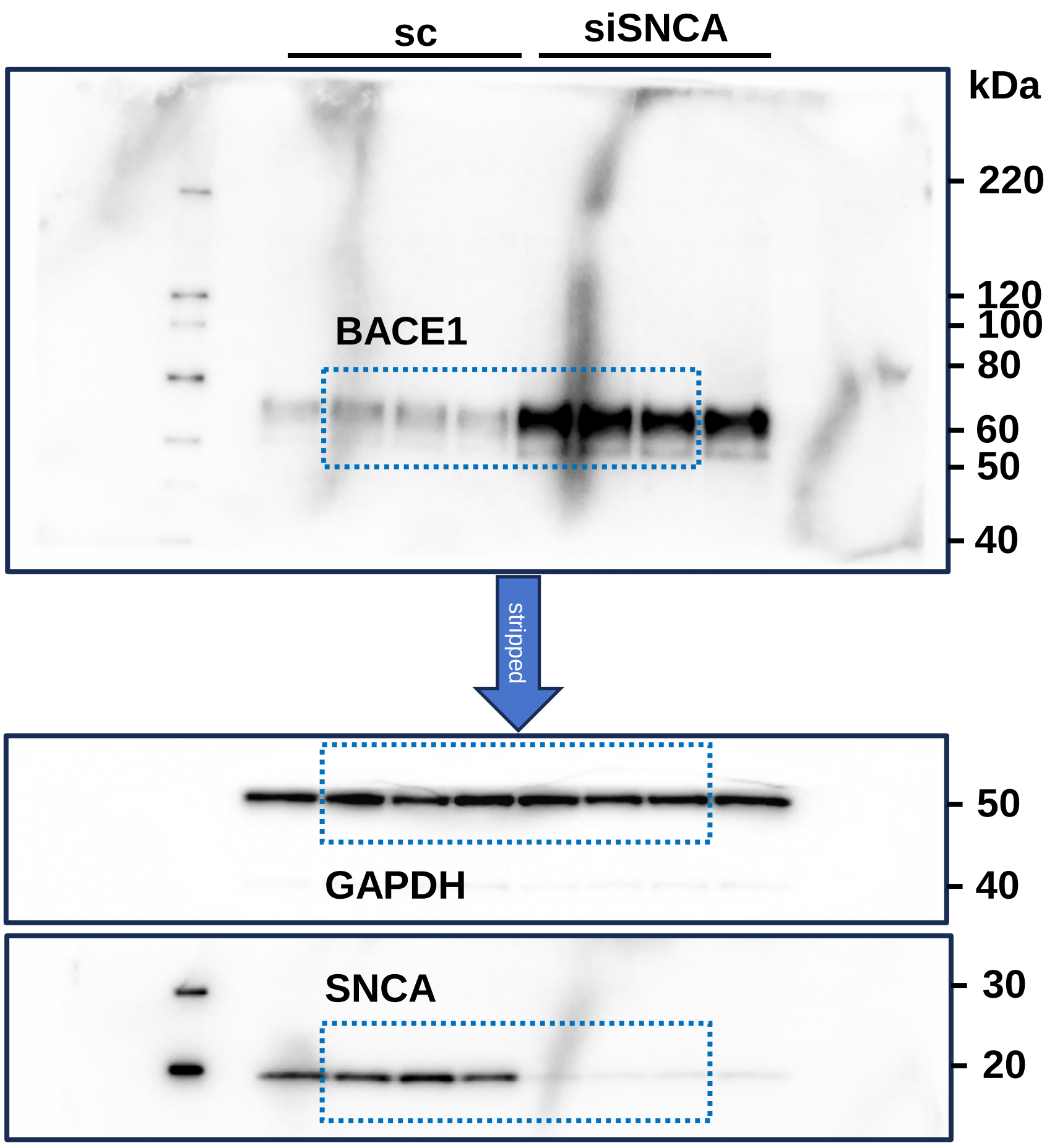

Unedited full blot for Figure 4G-1.
(for representative figure and densitometric analysis).

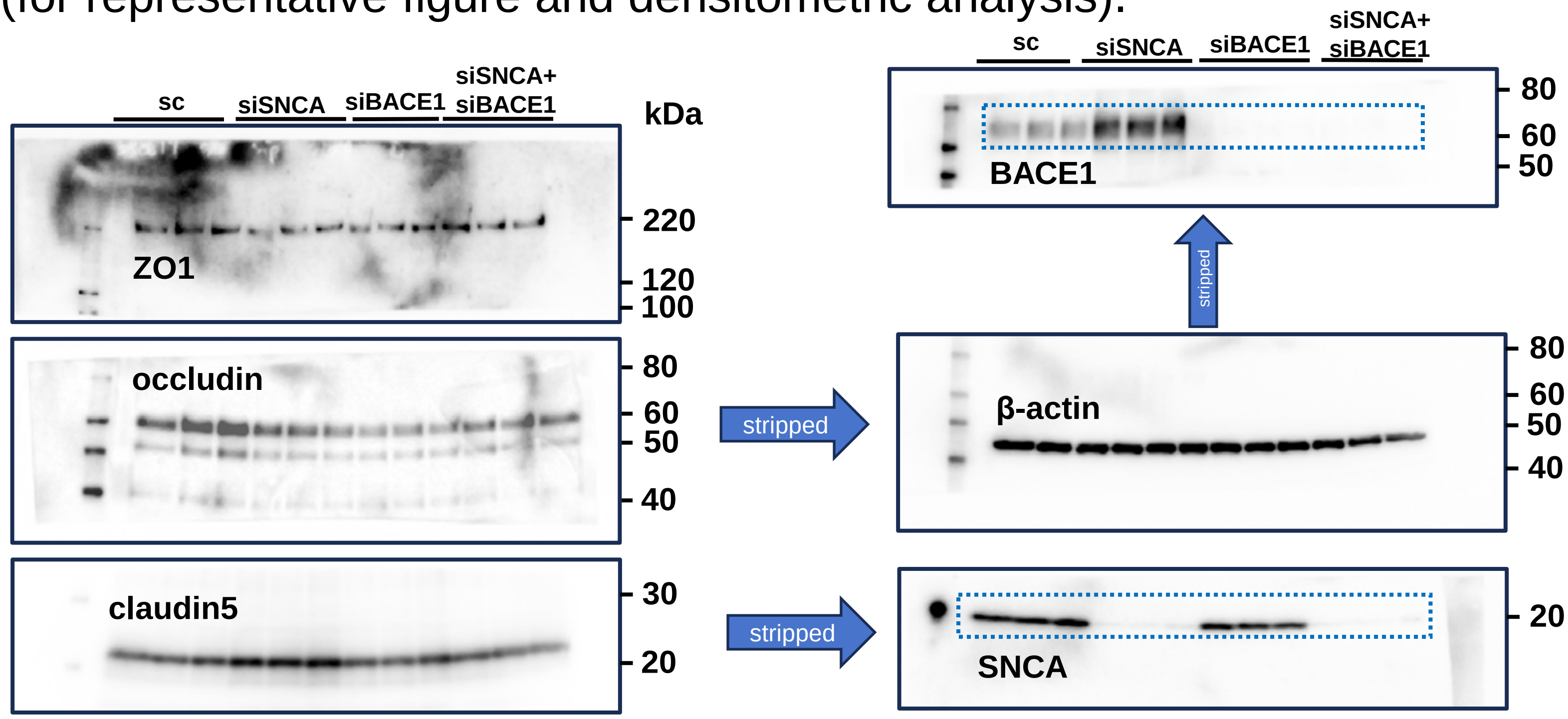

Unedited full blot for Figure 4G-2.
(for densitometric analysis).

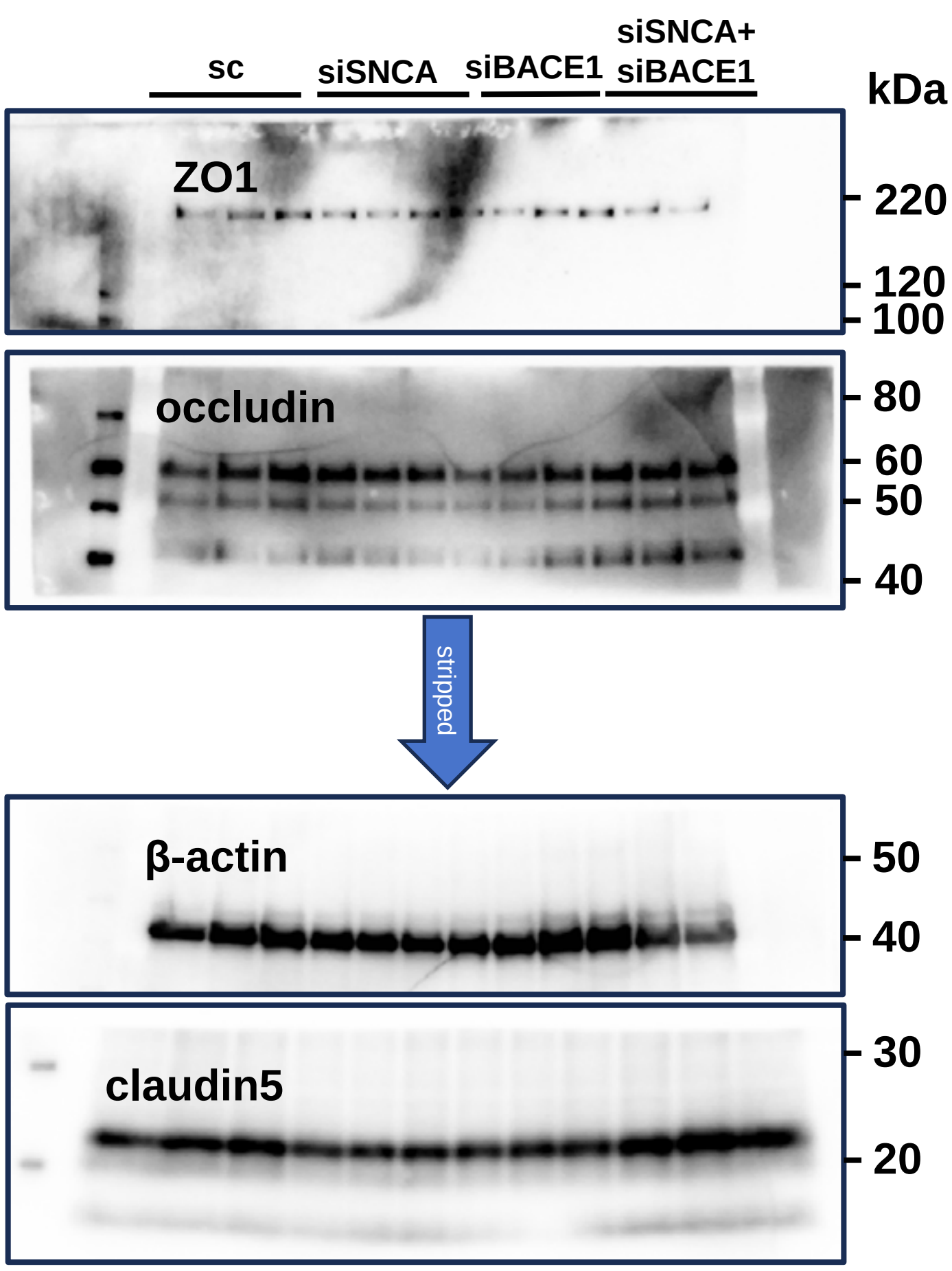

Unedited full blot for Figure 4G-3.
(for representative figure and densitometric analysis).

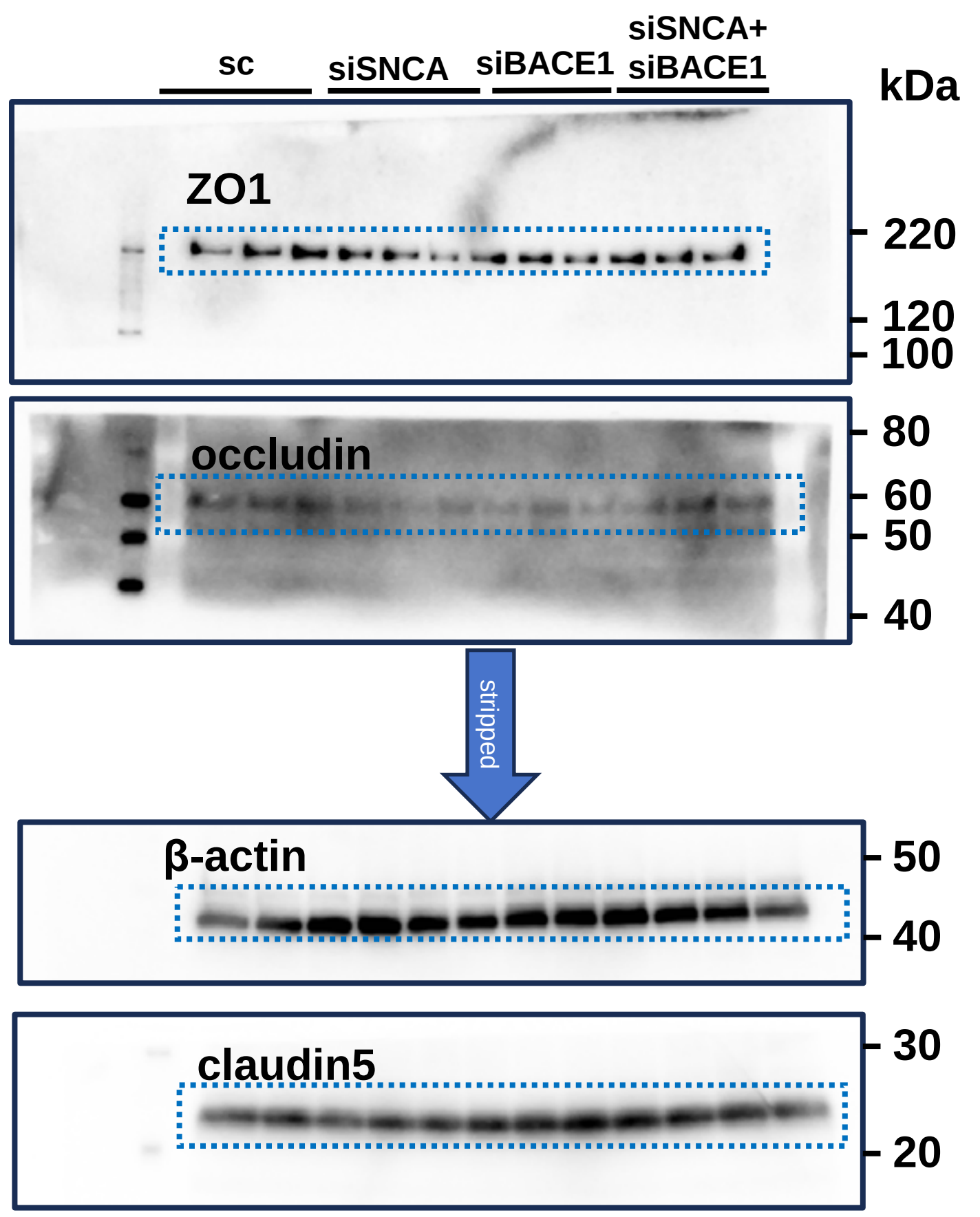

Unedited full blot for Figure 5A. (for representative figure and densitometric analysis).

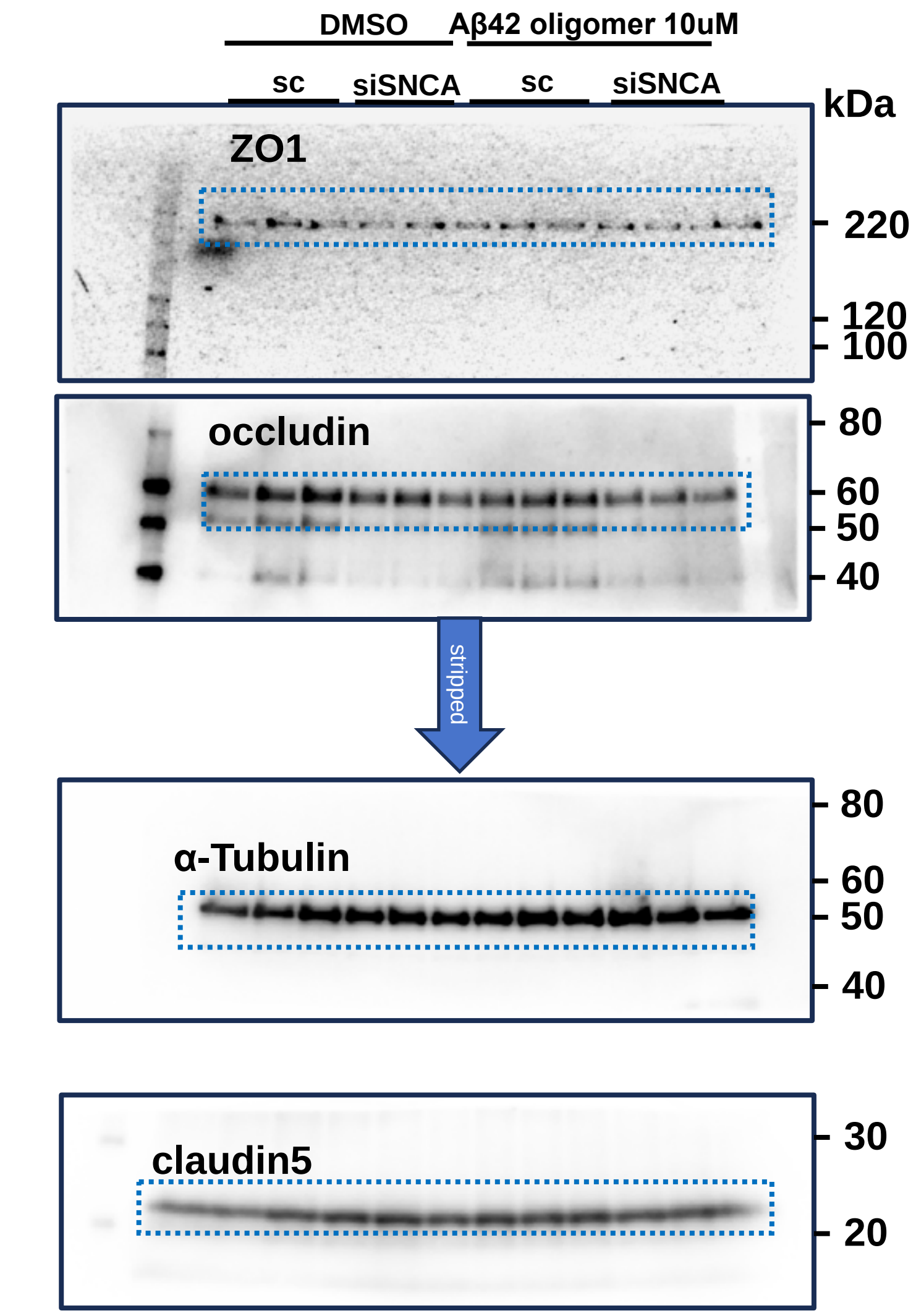

Unedited full blot for Figure 6D. (for representative figure and densitometric analysis).

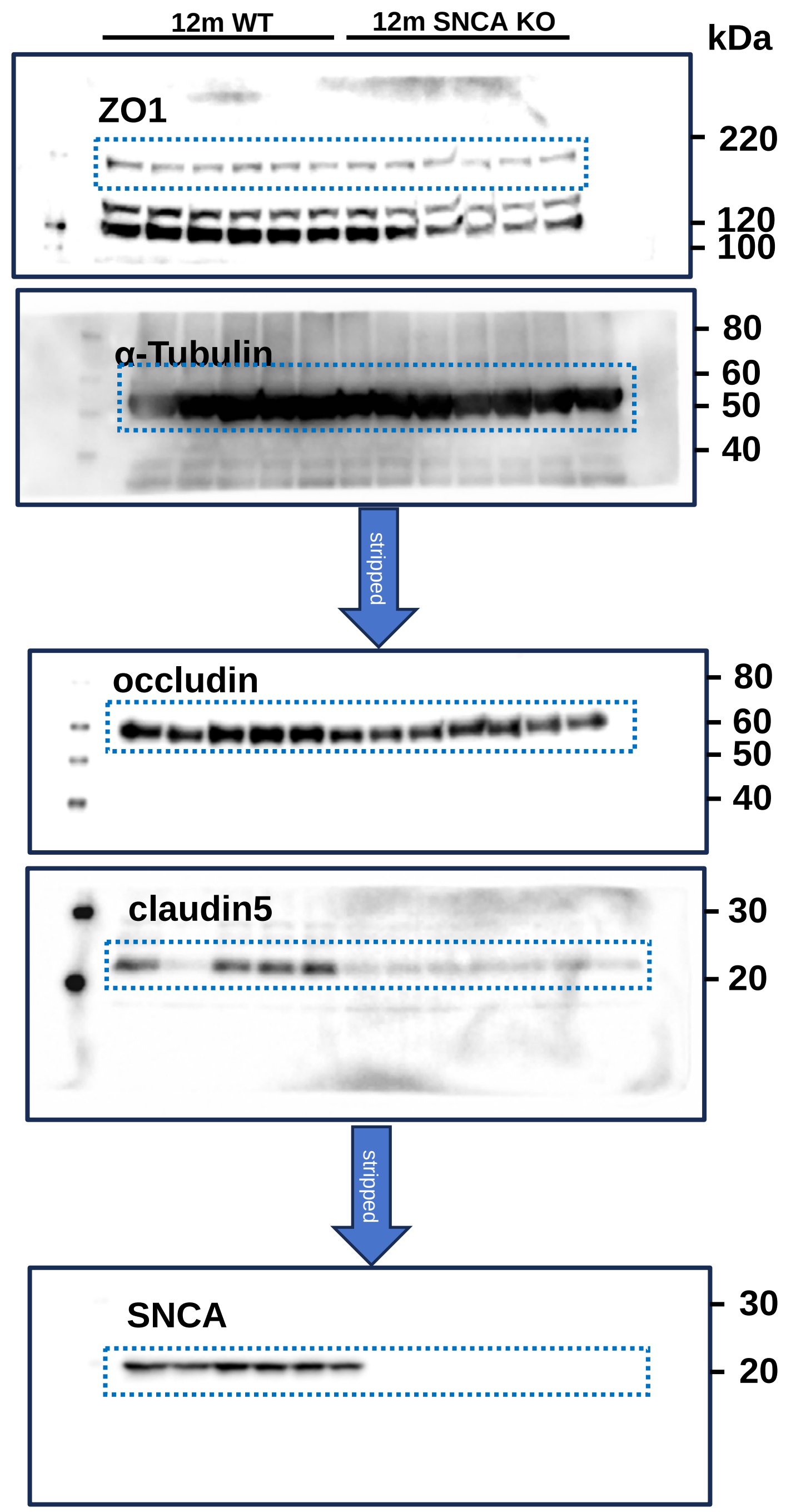

Unedited full blot for Figure 6E. (for representative figure and densitometric analysis).

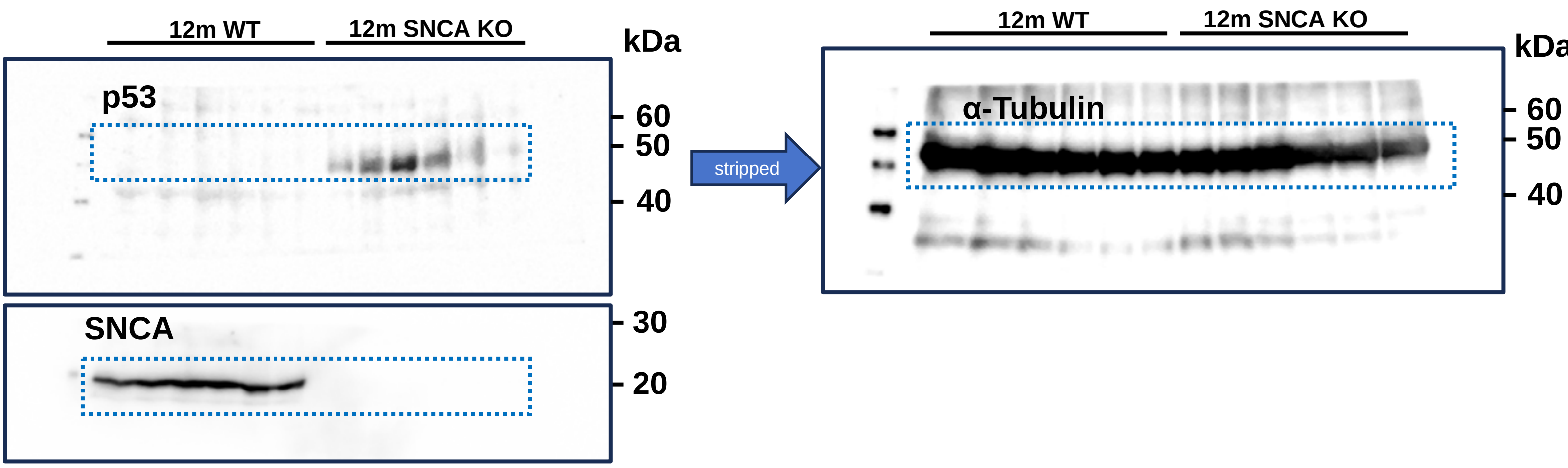

Unedited full blot for Figure 6J, 6L.
(for representative figure and densitometric analysis).

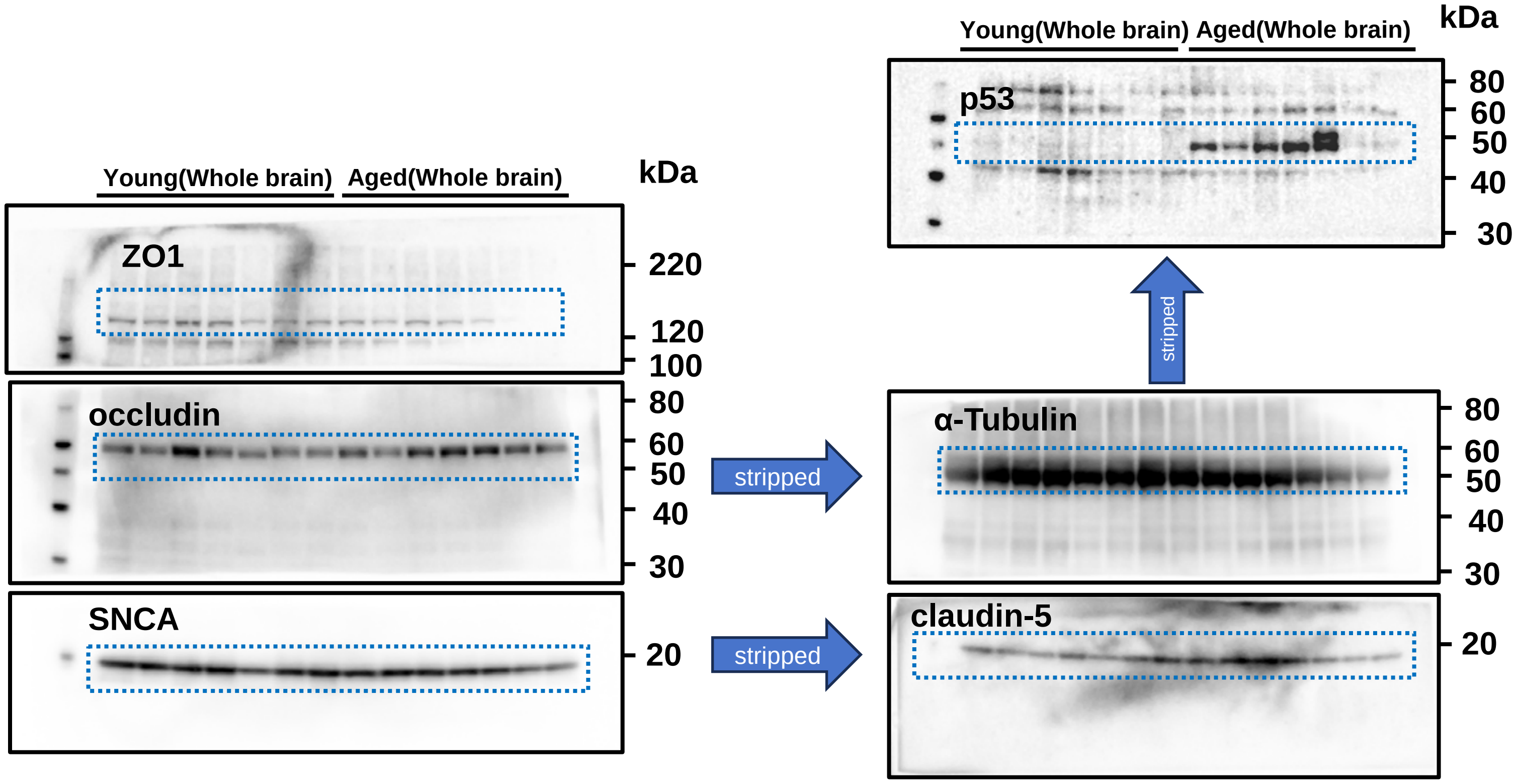

Unedited full blot for Figure 6K, 6M.
(for representative figure and densitometric analysis).

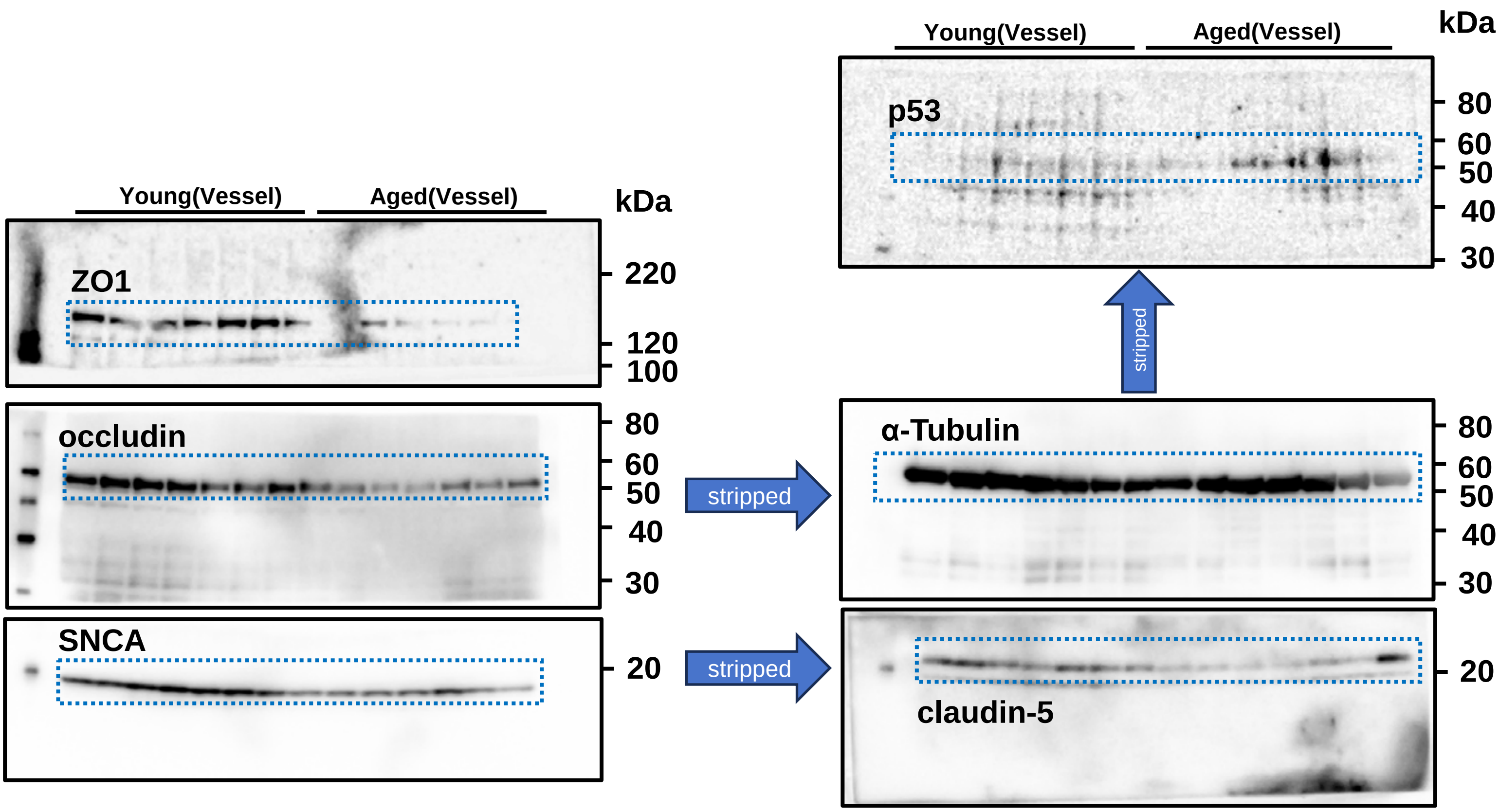

Unedited full blot for Supplementary Figure 1A.
(for representative figure and densitometric analysis).

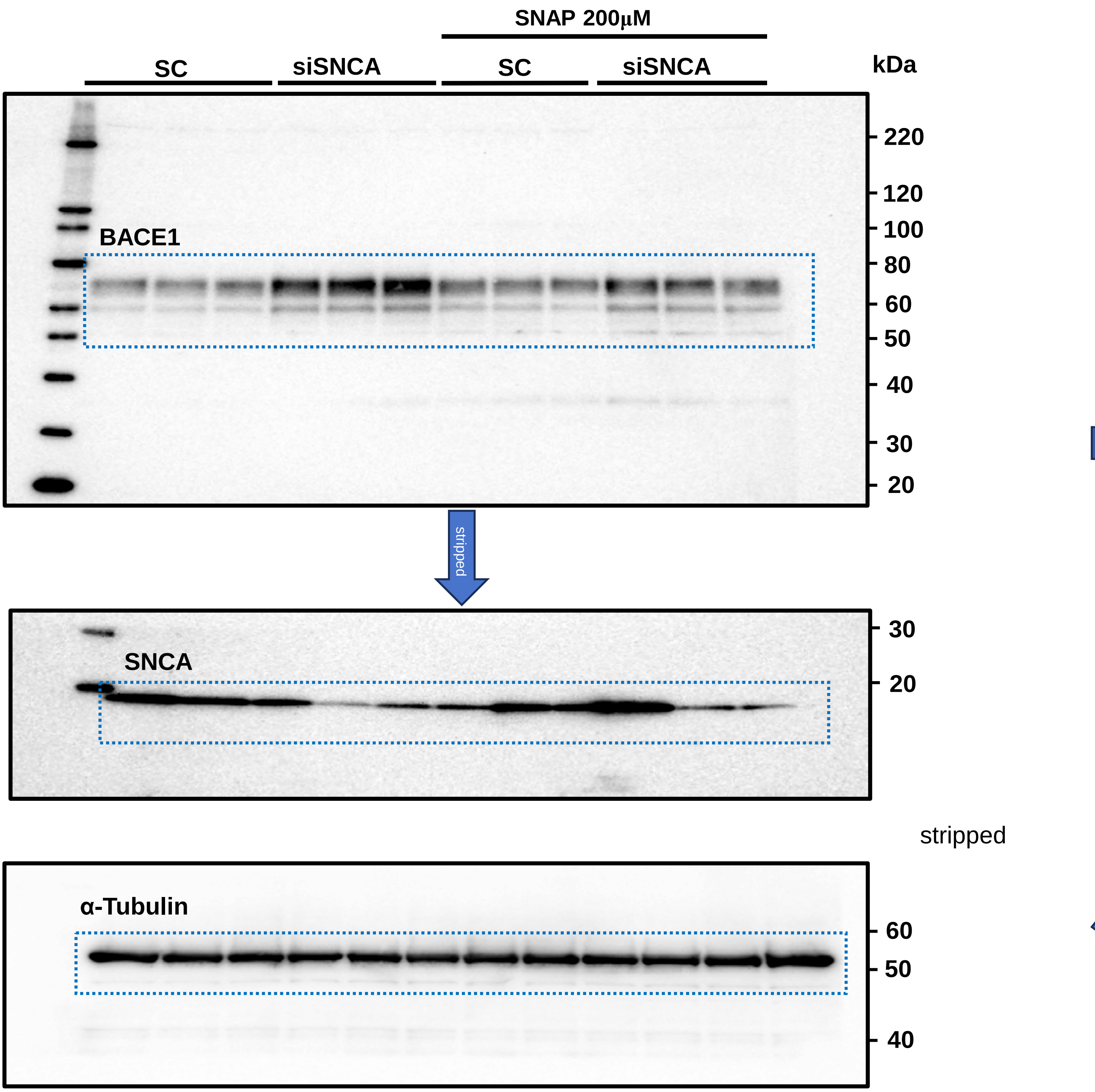

Unedited full blot for Supplementary Figure 2A.
(for representative figure and densitometric analysis).

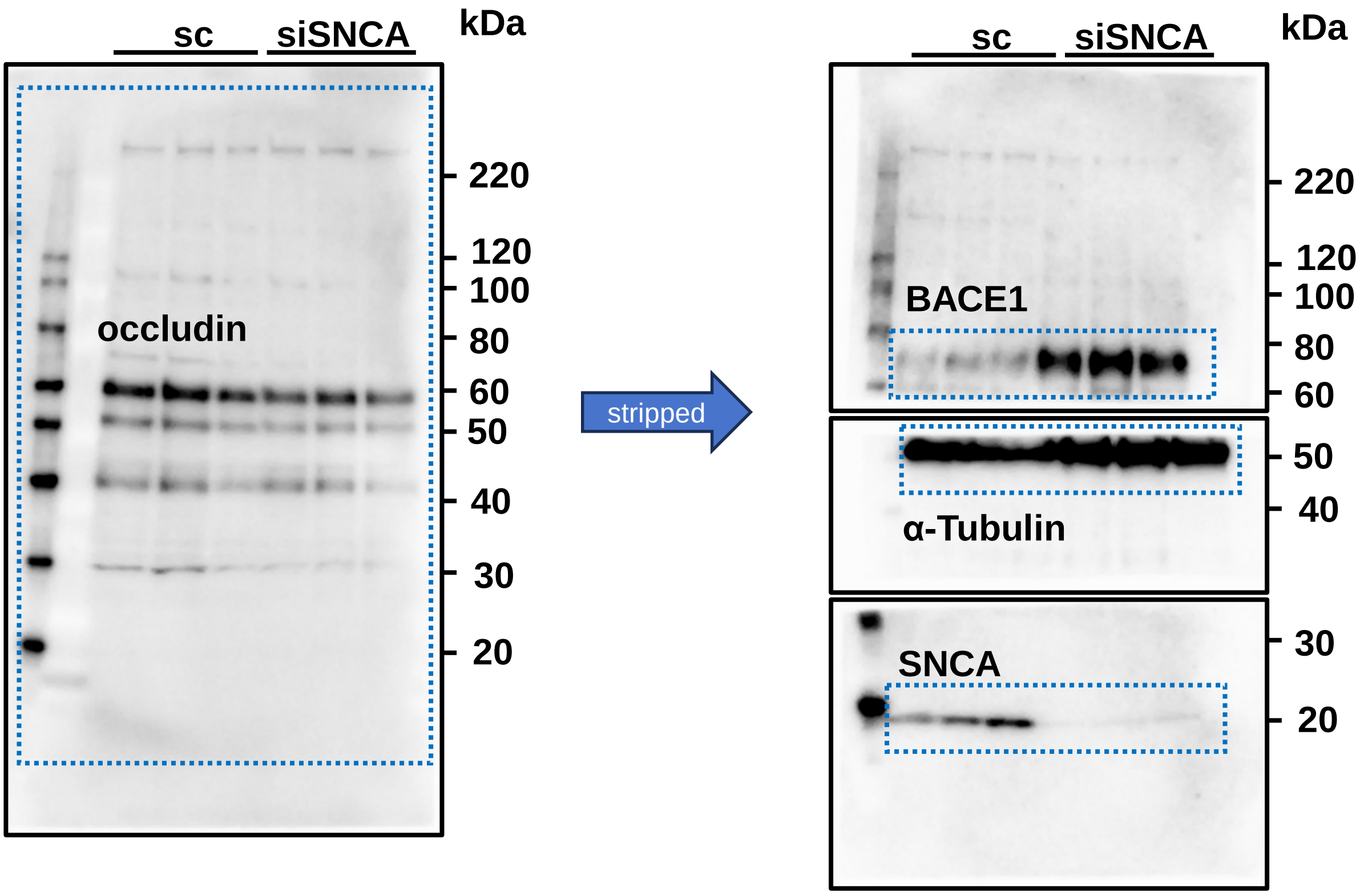

Unedited full blot for Supplementary Figure 2C.
(for representative figure and densitometric analysis).

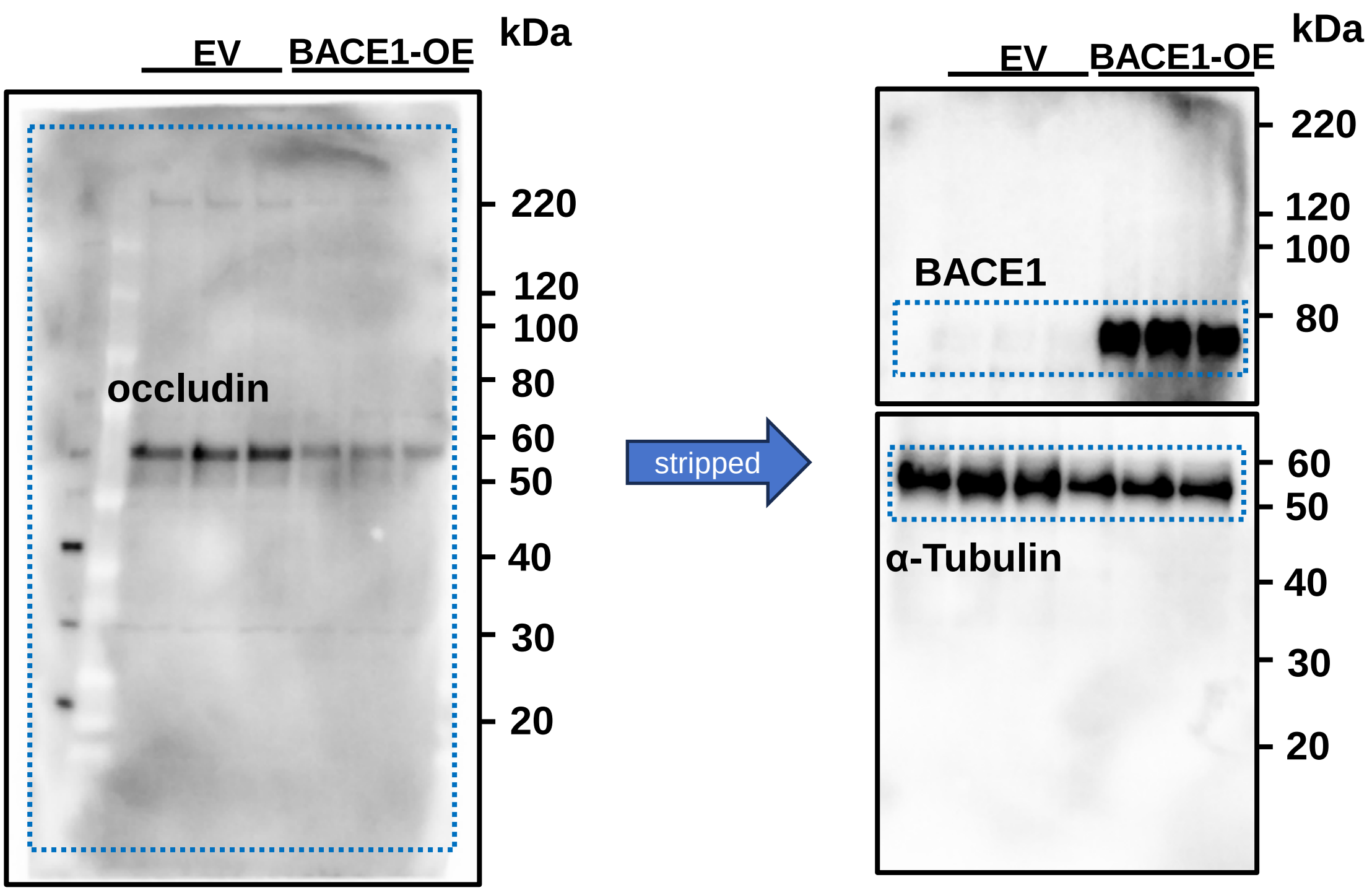

Unedited full blot for Supplementary Figure 5A.
(for representative figure).

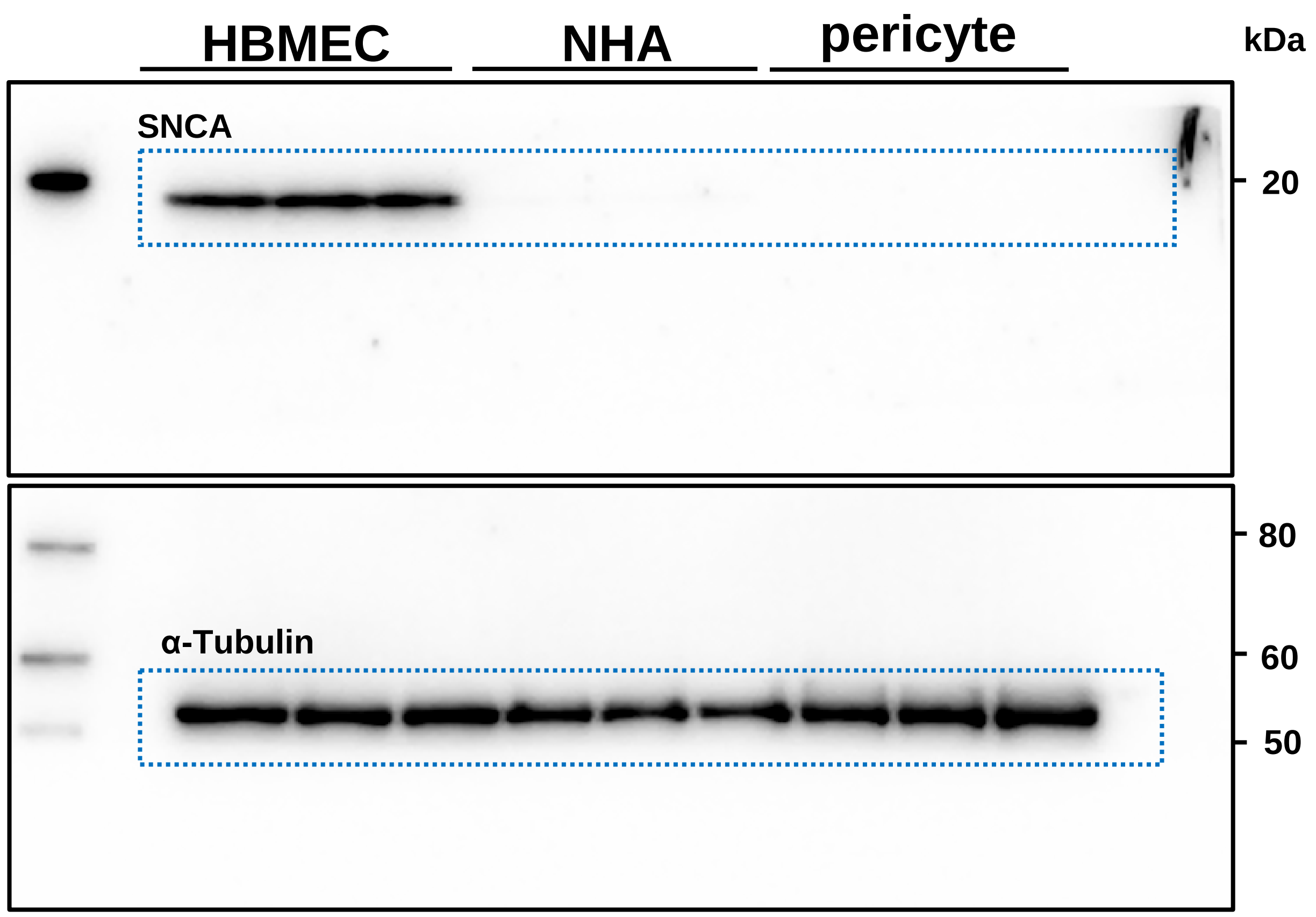

Unedited full blot for Supplementary Figure 6C, 6E.
(for representative figure and densitometric analysis).

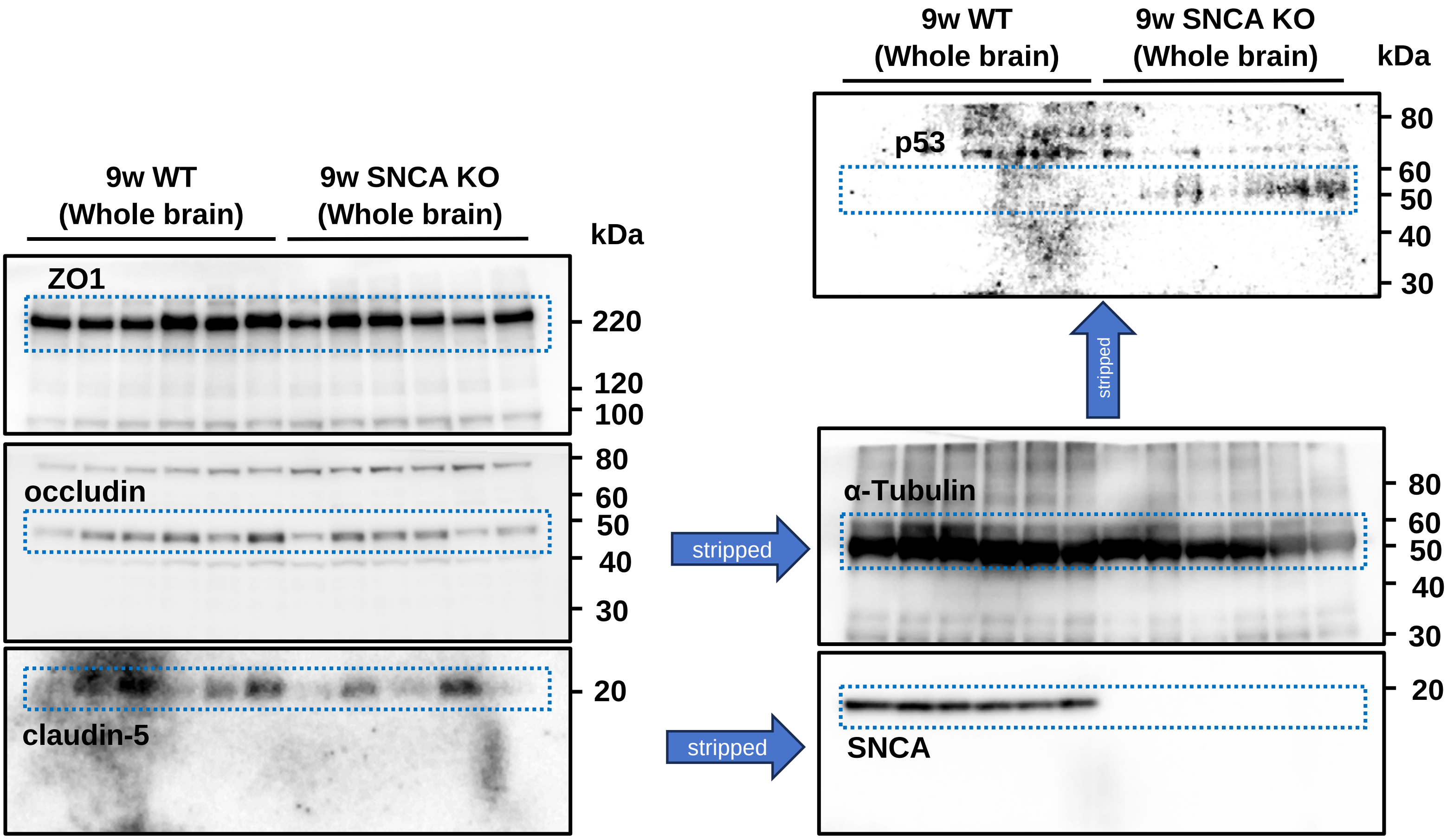

Unedited full blot for Supplementary Figure 6D, 6F.
(for representative figure and densitometric analysis).

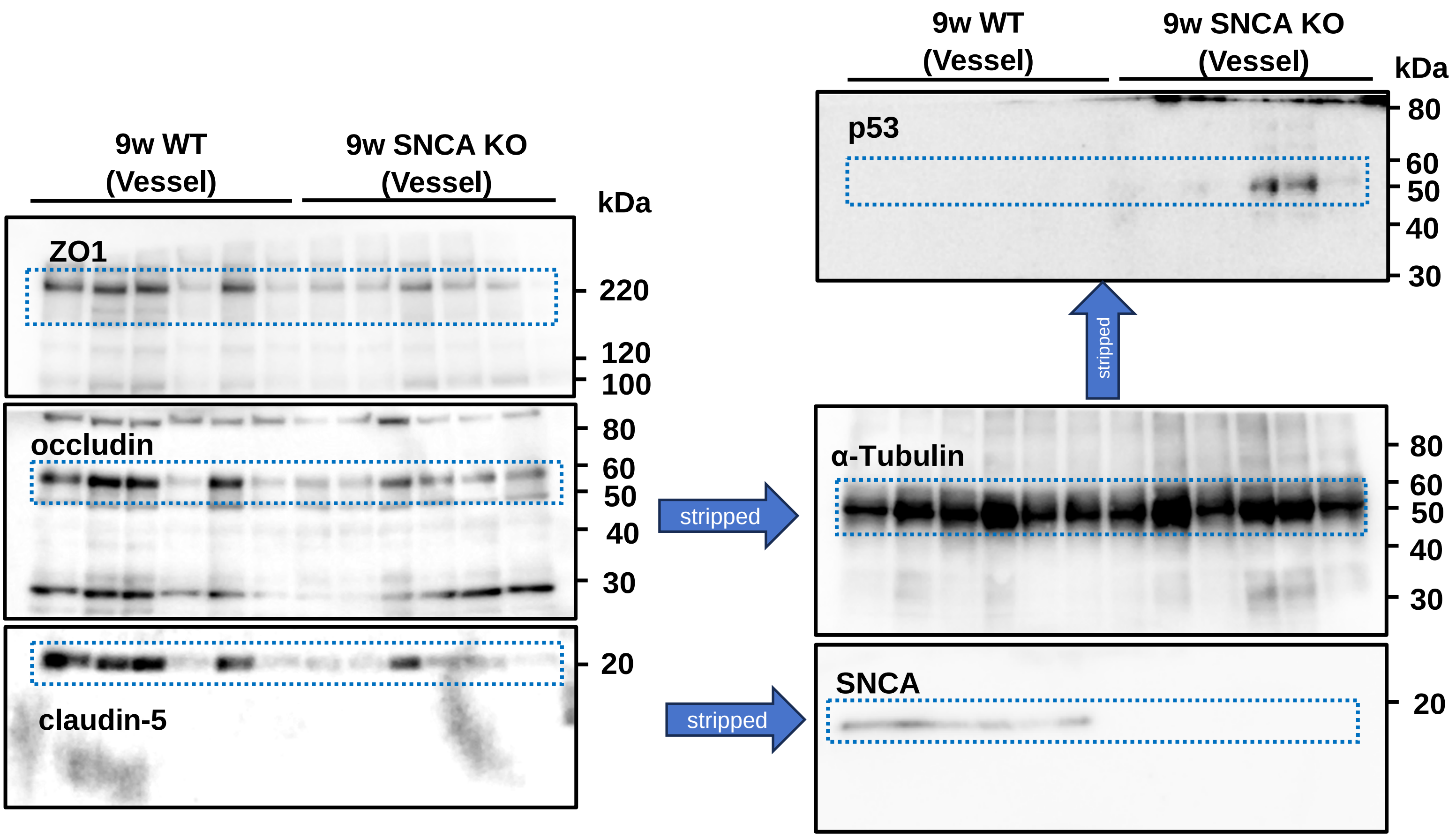

Unedited full blot for Supplementary Figure 7A.
(for representative figure and densitometric analysis).

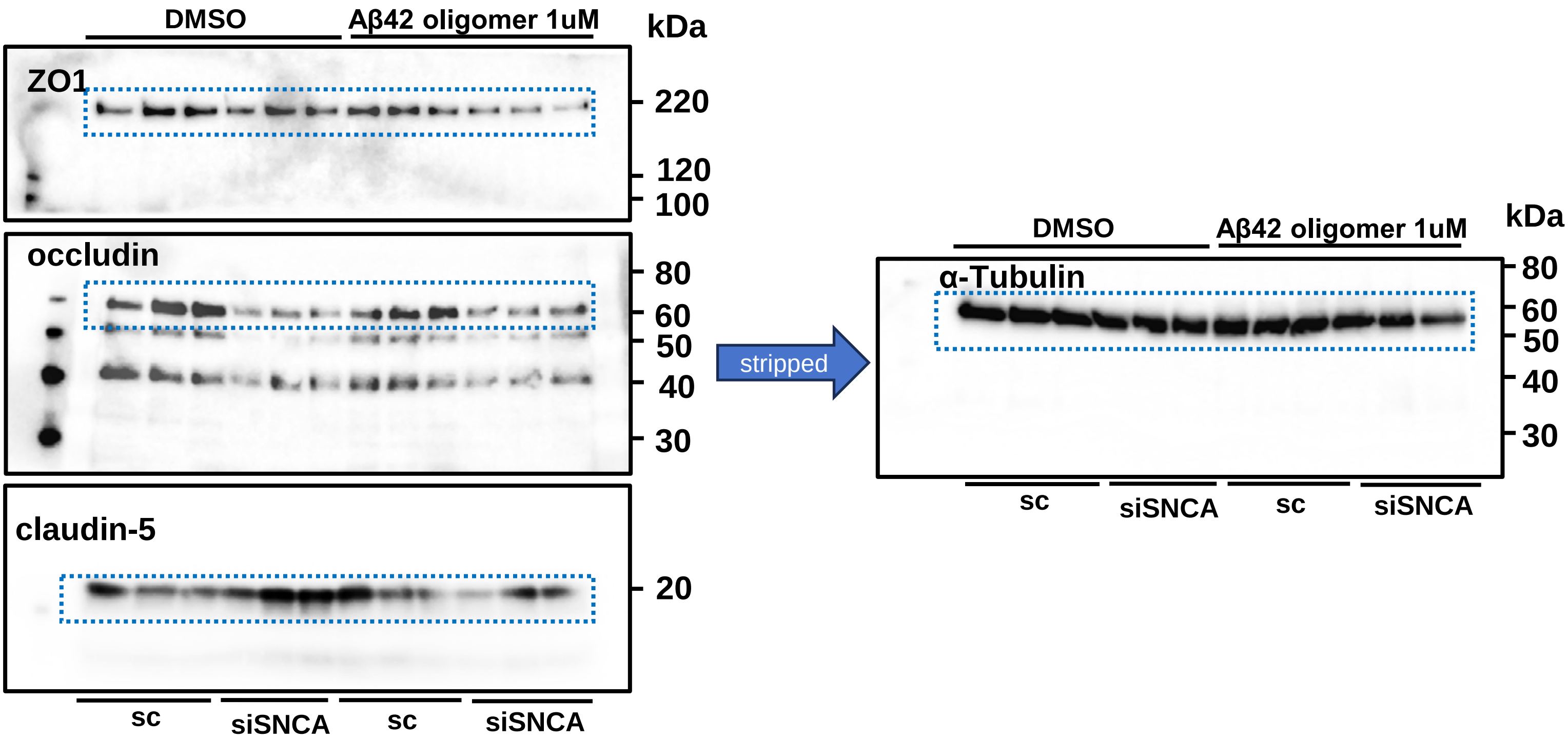

Unedited full blot for Supplementary Figure 8A.
(for representative figure and densitometric analysis).

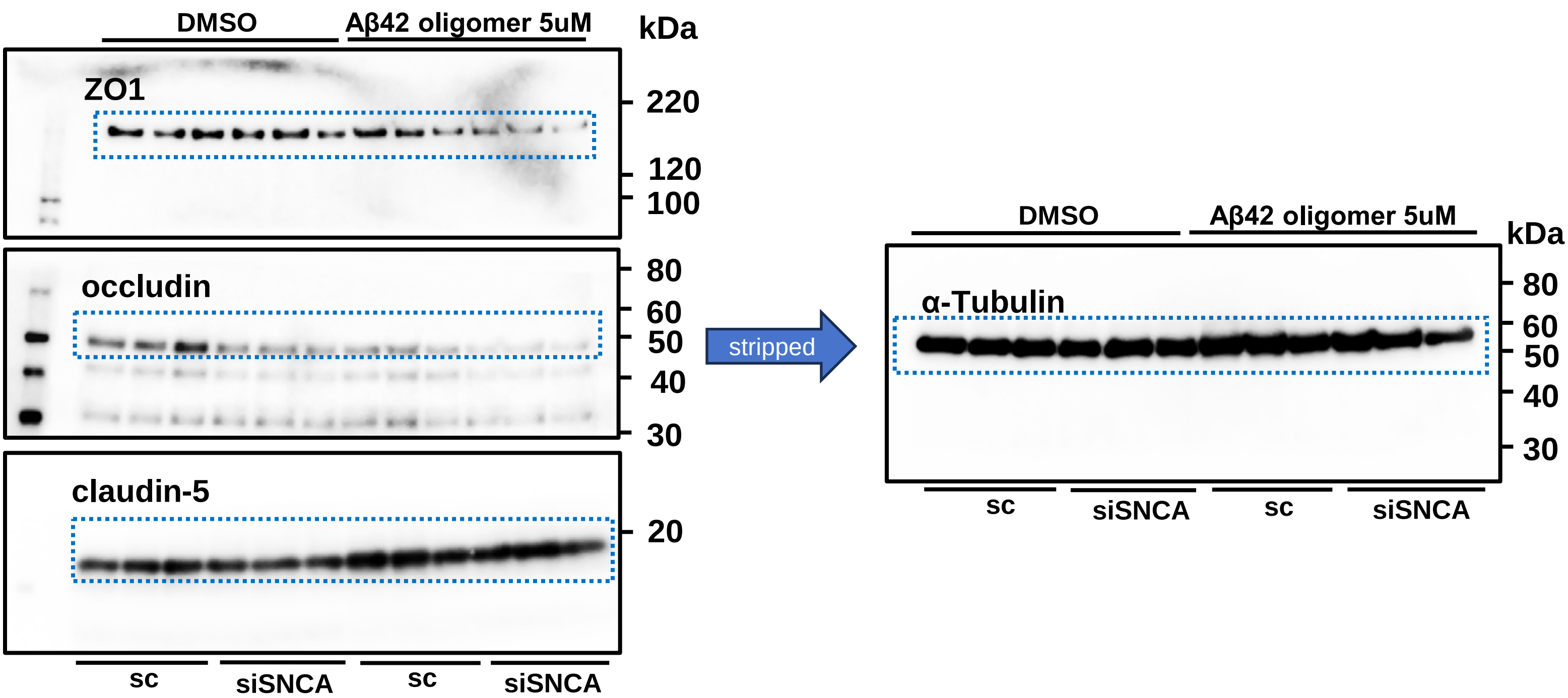

Supplement: Supplementary file 2 [file ACEL-25-e70770-s003.pdf]
