# Supplementary material for: Senescence‐Associated Loss of α‐Synuclein in Brain Microvascular Endothelium Compromises Tight Junctions and Disrupts Blood–Brain Barrier Integrity
Source: Aging Cell. 2026 Nov 9;25(11):e70770. doi: 10.1111/acel.70770 (PMC13746884; doi:10.1111/acel.70770)
Supplement: Supplementary file 3 [file ACEL-25-e70770-s001.docx]

**Table S1. siRNAs used in this study**

| **Target Gene** | **Company** | **Catalog/siRNA ID** |
| --- | --- | --- |
| α-synuclein siRNA(h) | Santa Cruz Biotechnology | sc-29619 |
| Control siRNA-A | Santa Cruz Biotechnology | sc-37007 |
| BACE siRNA(h) | Santa Cruz Biotechnology | sc-372245 |

**Table S2. Plasmid used in this study**

| Plasmid/Vector | **Company** | Catalog/Plasmid ID |
| --- | --- | --- |
| pcDNA3.1/Zeo(+) Mammalian Expression Vector | Thermo Fisher | V86020 |
| pcDNA3.1-Zeo_BACE1 | Addgene | Plasmid#227105 |

**Table S3. Primers used in this study**

| **Name** | **Forward Primer sequence** | **Reverse Primer sequence** |
| --- | --- | --- |
| human SNCA | TCAAAAAGGACCAGTTGGGCAAG | ACCGAAATGCTGAGTGGGGG |
| human GAPDH | CGGAGTCAACGGATTTGGTCGTAT | AGCCTTCTCCATGGTGGTGAAGAC |
| human BACE1 | TACCAACCAGTCCTTCCGC | CTCCCATAACAGTGCCCGT |
| human OCLN | TGCATGTTCGACCAATGC | AAGCCACTTCCTCCATAAGG |
| human TJP1 | CAGCCGGTCACGATCTCCT | TCCGGAGACTGCCATTGC |
| human CLDN5 | CTGCTGGTTCGCCAACATT | TGCGACACGGGCACAG |
| human CDKN2A (p16) | CGTACCCCGATTCAGGTGAT | TTGAGCAGAAGAGCTGCTACGT |
| human CDKN1A (p21) | AGCAGAGGAAGACCATGTGGA | AATCTGTCATGCTGGTCTGCC |
| human TP53 (p53) | CACGAGCTGCCCCCAGG | TCAGTCGACGTCTGAGT |

**Table S4. Antibodies used in this study**

| **Target** | **Antibody** | **Company** | **Catalog #** | **Dilution** |
| --- | --- | --- | --- | --- |
| α-Synuclein | Rabbit polyclonal | Cell Signaling Technology | #2642 | 1:1000 |
| eNOS | Rabbit polyclonal | Cell Signaling Technology | #9572 | 1:1000 |
| α-tubulin | Rabbit monoclonal | Cell Signaling Technology | #2125 | 1:1000 |
| β-actin | Rabbit monoclonal | Cell Signaling Technology | #4970 | 1:1000 |
| GAPDH | Rabbit monoclonal | Cell Signaling Technology | #2118 | 1:1000 |
| SIRT1 | Rabbit polyclonal | Cell Signaling Technology | #2493 | 1:1000 |
| glypican-1 | Mouse monoclonal | Santa Cruz Biotechnology | sc-101827 | 1:500 |
| BACE1 | Rabbit monoclonal | Cell Signaling Technology | #5606 | 1:1000 |
| ZO-1 (WB) | Rabbit monoclonal | Cell Signaling Technology | #13663 | 1:1000 |
| Occludin (WB) | Rabbit monoclonal | Cell Signaling Technology | #91131 | 1:1000 |
| Claudin-5 (WB) | Rabbit monoclonal | Cell Signaling Technology | #49564 | 1:1000 |
| Occludin (IF/WB) | Rabbit polyclonal | Proteintech | 13409-1-AP | 1:100 |
| ZO-1 (IF) | Rabbit polyclonal | Proteintech | 21773-1-AP | 1:100 |
| Claudin-5 (IF) | Rabbit polyclonal | Proteintech | 29767-1-AP | 1:100 |
| CD31 (IF) | Rat monoclonal | BD Bioscience | 55074 | 1:200 |
| CD31 (WB) | Rabbit monoclonal | Cell Signaling Technology | #77699 | 1:1000 |
| P53 (WB/IF) | Mouse monoclonal | Cell Signaling Technology | #2524 | 1:1000(WB)/1:100(IF) |
| donkey anti-rat IgG (H+L), highly cross-adsorbed, Alexa Fluor Plus 555 | Donkey polyclonal | Invitrogen | #  A48270 | 1:200 |
| donkey anti-rabbit IgG (H+L), highly cross-adsorbed, Alexa Fluor 647 | Donkey polyclonal | Invitrogen | # A-31573 | 1:200 |
| goat anti-mouse IgG (H+L), highly cross-adsorbed secondary antibodies, Alexa Fluor 647 | Goat polyclonal | Invitrogen | # A-21236 | 1:200 |
